# Design, synthesis, and evaluation of curcumin analogues as potential inhibitors of bacterial sialidase

**DOI:** 10.1080/14756366.2018.1488695

**Published:** 2018-08-21

**Authors:** Bo Ram Kim, Ji-Young Park, Hyung Jae Jeong, Hyung-Jun Kwon, Su-Jin Park, In-Chul Lee, Young Bae Ryu, Woo Song Lee

**Affiliations:** a Bio-processing Technology Development and Support Team, Korea Research Institute of Bioscience and Biotechnology, Jeongeup, Republic of Korea;; b Natural Product Material Research Center, Korea Research Institute of Bioscience and Biotechnology, Jeongeup, Republic of Korea

**Keywords:** Curcumin, sepsis, sialidase, Nan A

## Abstract

Sialidases are key virulence factors that remove sialic acid from the host cell surface glycan, unmasking receptors that facilitate bacterial adherence and colonisation. In this study, we developed potential agents for treating bacterial infections caused by *Streptococcus pneumoniae* Nan A that inhibit bacterial sialidase using *Turmeric* and curcumin analogues. Design, synthesis, and structure analysis relationship (SAR) studies have been also described. Evaluation of the synthesised derivatives demonstrated that compound **5e** was the most potent inhibitor of *S. pneumoniae* sialidase (IC_50_ = 0.2 ± 0.1 µM). This compound exhibited a 3.0-fold improvement in inhibitory activity over that of curcumin and displayed competitive inhibition. These results warrant further studies confirming the antipneumococcal activity **5e** and indicated that curcumin derivatives could be potentially used to treat sepsis by bacterial infections.

## 1. Introduction

Sepsis, a life-threatening organ dysfunction caused by a dysregulated host response to infection, is caused by an overwhelming immune response to an existing bacterial infection^1^. It commonly occurs in the ageing population and results in ∼20–30 million cases annually worldwide. Overall, sepsis remains one of the top five causes of death worldwide^2^, also the mortality rate is ∼20–50% for severe sepsis and 40–80% for septic shock. Especially, bacterial sepsis is a major cause of mortality of hospitalised patients, thus the development of drugs for bacterial sepsis is urgently needed and many efforts have been undertaken in the medicinal and pharmaceutical industry.

The Gram-positive bacterium, *Streptococcus pneumoniae,* is one of the causes of sepsis. It also major human pathogen and causes a variety of diseases, including bacterial meningitis, otitis media, pneumonia, conjunctivitis^3–6^. Several virulence factors contribute to colonisation and early infection processes, above all sialidases from bacteria are considered key virulence factors^7^. Sialidase removes the terminal sialic acid residues from host cell surface glycans, unmasking receptors that facilitate bacterial adherence and colonisation^8^. This process causes resistance to penicillin and other antibiotics that are used to treat *S. pneumoniae* infection^9^. According to known literature, all clinical isolates of *S. pneumoniae* have sialidases activity known to be involved in sepsis^10^. *S. pneumoniae* sialidase genes in clinical pneumococcal isolates determined that Nan A, Nan B, and Nan C are present in 100%, 96%, and 51% of these strains. Among these sialidases, Nan A has been shown to play an essential role in host–pneumococcal interactions in the respiratory tract and sepsis in mouse models^7^ ^,^ ^11^ ^,^ ^12^ ^,^ ^33^. Therefore, high-affinity inhibitors that can block Nan A are potential agents for prevention and treating sepsis. In the last few years, several studies have reported the discovery of viral or bacterial sialidase inhibitors from an isolated natural product such as flavonoids, coumarins, diplacone, mimulone, pterocarpans, and phlorotannins. However, these compounds are known inhibitors as *Clostridium perfringens* (*Cp*-Nan I) or viral sialidase^9^ ^,^ ^14–17^. Recently, some studies have reported to inhibition of *S. pneumoniae* sialidase such as diazenylaryl sulphonic acids, malabaricone C, Artocarpin, and anthraquinone glycosides^9^ ^,^ ^10^ ^,^ ^18–20^. Therefore, to develop novel bacterial sialidase inhibitors, we focused on the natural product, *Turmeric*, because it had not yet been evaluated.

*Turmeric* has been used as a traditional medicine for conditions such as liver disease^21^, indigestion^22^, rheumatoid arthritis^23^, and insect bites^24^ and is consumed daily by millions of people for the treatment of various diseases. Curcumin is the primary component of *Turmeric* and has a feruloyl methane group containing methoxy, hydroxyl, and heptadienyl with a 1,3-diketone moiety. Curcumin has been extensively studied in the past few decades as an important therapeutic compound. In addition, it still receives a lot of attention for its biological properties, including anti-inflammatory, anti-viral, anti-bacterial, anti-cancer, anti-oxidant, and anti-carcinogenic activities^25^, and its use in debilitating diseases such as Crohn’s disease, ulcerative colitis^26^, and Alzheimer’s disease^27^ ^,^ ^28^. Therefore, many studies evaluating the biological activity of curcumin have been performed and potential curcuminoids have been developed for several diseases.

**Scheme 1.**
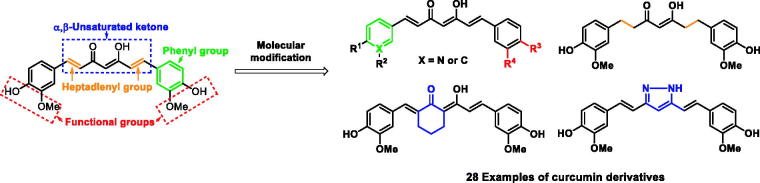
Designed strategies for the synthesis of curcumin analogues.

In this study, we report that *Turmeric* and curcumin derivatives can targeting the *S. pneumoniae* Nan A. Designed strategies for synthesis of curcumin analogues are shown Scheme 1.

## 2. Materials and methods

### 2.1 General

All the chemical reagents used in this work and curcumin (**4a**) were purchased from commercial suppliers (Aldrich, St. Louis, MO; TCI, Japan; Alfa Aesar, Haverhill, MA or Acros Organics, USA companies) and used without further purification. The ^1^H and ^13 ^C NMR spectra were recorded using a JEOL ECA-500 spectrometer, Japan at 500 MHz and 125 MHz, respectively, with chemical shift (*δ*) values reported in ppm unit. Multiplicities are describes as singlet (s), doublet (d), doublet of doublet (dd), triplet (t), multiplet (m), and broad exchangeable proton (bs). High resolution mass spectra were obtained on a GC Mate 2, JEOL. A CEM Discover system (No. 908005) fitted with a temperature controller was used for microwave reactions. Irradiation was initiated at 300 W to raise the temperature to the set point (150 °C). Reactions were monitored by thin-layer chromatography (TLC) with Merck’s DC-Fertigiplatterm Kiegel 60 GE254 plates. Visualisation was accomplished with either UV light or by immersion in a solution of phosphomolybdic acid (PMA) followed by heating on a hot plate for ∼10 s. The reaction products were purified by open column chromatography using silica gel produced by Merck (Darmstadt, Germany) (Silica gel 60; 63–200 mesh, ASTM) or Cosmosil 140 C-18 OPN produced by Nacalai Tesque, Inc., USA.

### 2.2 Expression, purification, and preparation of *S. pneumoniae* Nan A

We have synthesised and expressed the full-length genes for the *S. pneumoniae* sialidase (Nan A) in *Escherichia coli*. The gene encoding Nan A (Figure S28, GenBank accession no. COT45929.1, PDB: 2VVZ) of *S. pneumoniae* TIGR4 was synthesised (Thermo Fisher Scientific GENEART GmbH, Regensburg, Germany). The synthesised gene was inserted into the cloning sites of a pET151/d-TOPO vector (Invitrogen, Carlsbad, CA) containing a 6x His-tag at the C-terminus. *S. pneumoniae* sialidase was expressed and purified from *E. coli* BL21 (DE3) (HIT; Real biotech Co., Taipei, Taiwan). The purified Nan A was detected at ∼56.6 kDa with greater than 90% purity using sodium dodecyl sulphate-polyacrylamide gel electrophoresis (SDS-PAGE) (Figure S29). The purified sialidase had specific activities (*K*_m_ values) of 43.9 µM using 4-methylumbelli-feryl-α-*D*-*N*-acetylneuraminic acid sodium salt hydrate (MUNANA; Catalogue No. M8639; Sigma) as substrate (Figure S30).

### 2.3 Enzyme inhibition activity

As described, the inhibitory effects of compounds on *S. pneumoniae* Nan A were measured using a fluorescence (FL)-based assay. In this assay, the 4-methylumbelliferyl-α-*D*-*N*-acetylneuraminic acid (Sigma Chemical Co., St. Louis, MO) was used as a substrate, and the enzyme activity was determined by measuring the increase in fluorescence by continuously monitoring the reactions at 450/40 nm with excitation at 365 nm using a SpectraMax M2e Multimode Reader (Molecular Devices Co.). The IC_50_ values of the synthesised compounds were measured in a reaction mixture containing enzyme (final concentration of Nan A, 2.2 nM), the test compounds (from 0 to 200 µM), and 50 µM of substrate in 20 mM Sodium phosphate buffer (pH 7.5, containing 300 mM NaCl). To determine the enzyme activity, the experimental data were fit to a logistic curve with Equation (1), a time-drive protocol was used and the initial velocity was recorded over a range of concentrations, and the data were analysed using a nonlinear regression program (Sigma Plot; SPCC Inc., Chicago, IL).

(1) $$\text{Inhibition}\;\text{activity}\;\left(\%\right)\;=100\;–\;[\left(S\right)\;–(S_{0})/(C\;-C_{0})]*100,$$

where *C* is the fluorescence of the control (enzyme, buffer, and substrate) after 60 min of incubation, *C*_0_ is the fluorescence of the control at 0 min, *S* is the fluorescence of the tested samples (enzyme, sample solution, and substrate) after incubation, and *S*_0_ is the fluorescence of the tested samples at 0 min.

### 2.4 Other sialidases assay

*Clostridium perfringens* sialidase (Catalogue No. 2876; Sigma) and *Vibrio cholerae* sialidase (Catalogue No. 72197; Sigma) activities were evaluated according to a method described by Lee et al.^9^ using 4-MUNANA as the substrate, where 10 µL of enzyme solution was mixed with 20 µL of 0.4 mM MUNANA in 20 mM sodium acetate buffer at pH 5.5 with 4 mM CaCl_2_ 150 mM NaCl. For the inhibition studies, *C. perfringens* sialidase and 0–200 µM of the individual compounds were mixed with MUNANA at 37 °C. The production of 4-methylumbelliferone was measured by monitoring the fluorometric determination at excitation wavelength 365 nm/emission wavelength 450 nm.

### 2.5. Enzyme kinetic study

The inhibition mechanism was determined, and the apparent inhibition constants (*K*_i_) for the respective sialidase (Nan A) were performed on the test compounds, for which the IC_50_ values were below 25 µM. The test compounds were studied at three different concentrations that were chosen based on the IC_50_ values obtained with each sialidases (∼1/2 × IC_50_, IC_50_, 2 × IC_50_). The concentrations of marker substrates were chosen (∼1/4 *K*_m_, 1/2 *K*_m_, *K*_m_) with regard to their Michaelis–Menten kinetics. The *K*_i_ values were calculated by nonlinear regression analysis by fitting different models of enzyme inhibition to the kinetic data using SigmaPlot Enzyme Kinetics Module 1.3 (SPSS Inc., Chicago, IL). The inhibition mechanism of the compounds was determined by comparing the statistical results, including the Akaike’s information criterion values, of different inhibition models and selecting the one with the best fit.

### 2.6 General procedures for the synthesis of curcumins and the characterisation of synthesised compounds

#### 2.6.1 Synthesis of curcumin derivatives using Pabon’s reaction (compounds 3, 4b, 4e, 5a–5p, 5r, 5s)

Boron trioxide (43.44 mmol) was added to a solution of 2,4-petadione (65.16 mmol for **3**, **4**) or monophenyl intermediate (**3**, 21.7 mmol) in ethyl acetate (100 ml) at ambient temperature. After stirring for 1 h at 90 °C, the corresponding benzaldehydes (21.7 mmol) and triethyl borate (21.7 mmol) in ethyl acetate were added to the reaction mixture. The mixture was stirred for 2 h at 90 °C, then *n*-BuNH_2_ (21.7 mmol, 1 equiv., 7% solution in ethyl acetate) was slowly added, and the mixture was stirred at 90 °C until the aldehydes disappeared on TLC monitoring. The reaction mixture was then cooled to 50 °C and 1 M HCl (aq.) was added. After stirring the mixture for an additional 1 h and cooling to room temperature, water (30 ml) and ethyl acetate (20 ml) were added. The reaction mixture was washed with water and brine until it was neutralised. The organic phase was dried over anhydrous MgSO_4_ and evaporated under reduced pressure. The residue was purified using open-bed column chromatography on silica gel or Cosmosil 140 C–18 OPN to yielded curcumin derivatives.

**5-Hydroxy-1,7-bis(3-hydroxy-4-methoxyphenyl)hepta-1,4,6-trien-3-one (4b):** Yield: 556 mg (23%); orange solid; mp: 182–183 °C (Lit^34^. 181–183 °C); ^1^H NMR (500 MHz, Acetone-d_6_) δ 3.83 (s, 3H), 3.85 (s, 3H), 3.87 (s, 3H), 5.98 (s, 1H), 6.64 (d, 1H, *J =* 15.5 Hz), 6.73 (d, 1H, *J =* 15.0 Hz), 6.97 (d, 1H, *J =* 1.5 Hz), 6.99 (d, 1H, *J =* 1.5 Hz), 7.11 (dd, 1H, *J*_1_ *=* 8.0 Hz, *J*_2_ = 2.0 Hz), 7.17 (d, 1H, *J =* 1.5 Hz), 7.21 (dd, 1H, *J*_1_ *=* 8.5 Hz, *J*_2_ = 1.5 Hz), 7.31 (d, 1H, *J =* 2.0 Hz); ^13 ^C NMR (125 MHz, Acetone-d_6_) δ 55.28, 55.46, 101.1, 110.3, 111.5, 111.6, 113.4, 121.7, 122.0, 122.8, 128.1, 128.5, 140.2, 140.3, 146.9, 149.7, 149.8, 151.6, 100.3, 114.1, 115.5, 183.3. HRMS (EI) *m/z*: [M]^+^ calcd. for C_21_H_20_O_6_ 368.1260; Found: 368.1261.

**5-Hydroxy-1,7-bis(4-hydroxyphenyl)hepta-1,4,6-trien-3-one (4e):** Yield: 378 mg (15%); orange solid; mp: 234–235 °C (Lit^35^. 232–233 °C); ^1^H NMR (500 MHz, Acetone-d_6_) δ 5.95 (s, 1H), 6.63 (s, 1H), 6.66 (s, 1H), 6.86–6.89 (m, 4H), 7.53 (s, 2H), 7.55 (s, 2H), 7.56 (s, 1H), 7.59 (s, 1H), 8.99 (bs, 2H); ^13 ^C NMR (125 MHz, Acetone-d_6_) δ 100.9, 115.9, 121.2, 126.9, 130.1, 140.2, 159.6, 183.7. HRMS (EI) *m/z*: [M]^+^ calcd. for C_19_H_16_O_4_ 308.1049; Found: 308.1047.

**7-(3,4-Dimethoxyphenyl)-5-hydroxy-1-(4-hydroxy-3-methoxyphenyl)hepta-1,4,6-trien-3-one (5a):** Yield: 245 mg (50%); dark orange solid; mp: 136–137 °C; ^1^H NMR (500 MHz, Acetone-d_6_) δ 3.83 (s, 3H), 3.85 (s, 3H), 3.89 (s, 3H), 5.95 (s, 1H), 6.70 (t, 2H, *J =* 6.7 Hz), 6.86 (d, 1H, *J =* 8.0 Hz), 6.97 (d, 1H, *J =* 8.0 Hz), 7.15 (dd, 1H, *J*_1_ *=* 8.0 Hz, *J*_2_ = 2.0 Hz), 7.20 (dd, 1H, *J*_1_ *=* 8.5 Hz, *J*_2_ = 1.5 Hz), 7.30 (dd, 2H, *J*_1_ *=* 4.7 Hz, *J*_2_ = 1.2 Hz), 7.57 (d, 2H, *J =* 16.0 Hz), 8.25 (bs, 1H); ^13 ^C NMR (125 MHz, Acetone-d_6_) δ 55.27, 55.45, 100.9, 110.3, 110.6, 111.6, 122.0, 122.7, 123.0, 128.1, 140.2, 140.7, 147.9, 149.2, 149.7, 151.6, 183.3, 183.9. HRMS (EI) *m/z*: [M]^+^ calcd. for C_22_H_22_O_6_ 382.1416; Found: 382.1416.

**7-(3,4-Dimethoxyphenyl)-5-hydroxy-1-(3-hydroxy-4-methoxyphenyl)hepta-1,4,6-trien-3-one (5b):** Yield: 274 mg (56%); dark orange solid; mp: 155–158 °C (Lit^34^. 157–158 °C); ^1^H NMR (500 MHz, Acetone-d_6_) δ 3.83 (s, 3H), 3.85 (s, 3H), 3.87 (s, 3H), 5.98 (s, 1H), 6.64 (d, 1H, *J =* 15.5 Hz), 6.73 (d, 1H, *J =* 15.0 Hz), 6.98 (dd, 2H, *J*_1_ *=* 8.2 Hz, *J*_2_ = 1.2 Hz), 7.11 (dd, 1H, *J*_1_ *=* 8.0 Hz, *J*_2_ = 2.0 Hz), 7.17 (d, 1H, *J =* 1.5 Hz), 7.21 (dd, 1H, *J*_1_ *=* 8.5 Hz, *J*_2_ = 1.5 Hz), 7.31 (d, 1H, *J =* 2.0 Hz), 7.54 (d, 1H, *J =* 16.0 Hz), 7.58 (d, 1H, *J =* 16.0 Hz), 7.86 (bs, 1H); ^13 ^C NMR (125 MHz, Acetone-d_6_) δ 55.20, 55.46, 101.1, 110.3, 111.5, 111.6, 113.4, 121.7, 122.0, 122.8, 128.1, 128.5, 140.2, 140.3, 146.9, 149.7, 149.8, 151.6, 183.3. HRMS (EI) *m/z*: [M]^+^ calcd. for C_22_H_22_O_6_ 382.1416; Found: 382.1412.

**5-Hydroxy-7-(4-hydroxy-3-methoxyphenyl)-1-(3-hydroxy-4-methoxyphenyl)hepta-1,4,6-trien-3-one (5c):** Yield: 212 mg (45%); red orange solid; mp: 137–140 °C; ^1^H NMR (500 MHz, Acetone-d_6_) δ 3.87 (s, 3H), 3.89 (s, 3H), 5.96 (s, 1H), 6.63 (d, 1H, *J =* 16.0 Hz), 6.69 (d, 1H, *J =* 15.0 Hz), 6.85 (d, 1H, *J =* 8.0 Hz), 6.97 (d, 1H, *J =* 8.5 Hz), 7.10 (dd, 1H, *J*_1_ *=* 8.5 Hz, *J*_2_ = 2.5 Hz), 7.15 (dd, 1H, *J*_1_ *=* 8.0 Hz, *J*_2_ = 2.0 Hz), 7.17 (d, 1H, *J =* 2.5 Hz), 7.32 (d, 1H, *J =* 1.5 Hz), 7.53 (d, 1H, *J =* 16.0 Hz), 7.57 (d, 1H, *J =* 16.0 Hz), 7.83 (bs, 1H), 8.18 (bs, 1H); ^13 ^C NMR (125 MHz, Acetone-d_6_) δ 55.44, 101.0, 110.9, 111.4, 113.5, 115.3, 121.4, 121.6, 122.0, 123.1, 140.1, 140.7, 146.8, 146.9, 147.9, 149.1, 149.2, 149.7, 183.3, 183.9. HRMS (EI) *m/z*: [M]^+^ calcd. for C_21_H_20_O_6_ 368.1260; Found: 368.1262.

**1-(3,4-Dihydroxyphenyl)-5-hydroxy-7-(4-hydroxy-3-methoxyphenyl)hepta-1,4,6-trien-3-one (5d):** Yield: 100 mg (20%); orange solid; mp: 160 °C (decomposed); ^1^H NMR (500 MHz, Acetone-d_6_) δ 3.89 (s, 3H), 5.94 (s, 1H), 6.58 (d, 1H, *J =* 13.0 Hz), 6.68 (d, 1H, *J =* 15.5 Hz), 6.86 (s, 2H), 7.03 (s, 1H), 7.15 (d, 1H, *J =* 4.0 Hz), 7.31 (s, 1H), 7.51 (d, 1H, *J =* 12.0 Hz), 7.56 (d, 1H, *J =* 16.0 Hz); ^13 ^C NMR (125 MHz, Acetone-d_6_) δ 55.48, 100.9, 110.6, 114.3, 115.3, 115.6, 121.2, 121.4, 121.8, 123.0, 127.3, 127.5, 140.4, 140.5, 145.5, 147.8, 147.9, 149.1, 183.0, 183.2. HRMS (EI) *m/z*: [M]^+^ calcd. for C_20_H_18_O_16_ 354.1103; Found: 354.1105.

**1-(3,4-Dihydroxyphenyl)-5-hydroxy-7-(3-hydroxy-4-methoxyphenyl)hepta-1,4,6-trien-3-one (5e):** Yield: 127 mg (28%); brown solid; mp: 203–204 °C; ^1^H NMR (500 MHz, Acetone-d_6_) δ 3.87 (s, 3H), 5.97 (s, 1H), 6.58 (d, 1H, *J =* 15.5 Hz), 6.64 (d, 1H, *J =* 16.0 Hz), 6.85 (s, 1H), 6.97 (d, 1H, *J =* 7.5 Hz), 7.04 (s, 1H), 7.11 (d, 1H, *J =* 7.0 Hz), 7.18 (s, 2H), 7.51 (d, 1H, *J =* 15.0 Hz), 7.52 (d, 1H, *J =* 16.0 Hz); ^13 ^C NMR (125 MHz, Acetone-d_6_) δ 55.46, 101.0, 111.5, 113.4, 115.5, 121.2, 121.6, 121.8, 122.0, 127.4, 128.5, 140.0, 140.6, 145.5, 146.9, 147.8, 149.7, 182.7, 183.6. HRMS (EI) *m/z*: [M]^+^ calcd. for C_20_H_18_O_6_ 354.1103; Found: 354.1104.

**1-(3-Ethoxy-4-hydroxyphenyl)-5-hydroxy-7-(4-hydroxy-3-methoxyphenyl)hepta-1,4,6-trien-3-one (5f):** Yield: 284 mg (58%); yellow solid; mp: 182–183 °C; ^1^H NMR (500 MHz, Acetone-d_6_) δ 1.37 (t, 3H, *J =* 7.0 Hz) , 3.89 (s, 3H), 4.15 (m, 2H), 5.94 (s, 1H), 6.66 (d, 1H, *J =* 2.5 Hz), 6.69 (d, 1H, *J =* 3.0 Hz), 6.85 (d, 2H, *J =* 8.5 Hz), 7.15 (dd, 2H, *J*_1_ *=* 10.0 Hz, *J*_2_ = 2.5 Hz), 7.30 (dd, 2H, *J*_1_ *=* 7.0 Hz, *J*_2_ = 1.5 Hz), 7.54 (d, 1H, *J =* 3.0 Hz), 7.58 (d, 1H, *J =* 3.0 Hz), 8.21 (d, 1H, *J =* 10.5 Hz); ^13 ^C NMR (125 MHz, Acetone-d_6_) δ 14.64, 55.93, 64.77, 101.3, 111.1, 112.1, 115.8, 121.9, 121.8, 123.3(4), 123.3(6), 123.4(3), 123.4(6), 127.7(4), 127.7(9), 141.0(0), 141.0(8), 147.5, 148.3, 149.5, 149.7, 184.0. HRMS (EI) *m/z*: [M]^+^ calcd. for C_22_H_22_O_6_ 382.1416; Found: 382.1417.

**5-Hydroxy-7-(4-hydroxy-3-methoxyphenyl)-1-(pyridin-3-yl)hepta-1,4,6-trien-3-one (5g):** Yield: 33 mg (8%); orange solid; mp: 185 °C (decomposed); ^1^H NMR (500 MHz, DMSO-d_6_) δ 3.80 (s, 3H), 6.12 (s, 1H), 6.81–6.84 (m, 2H), 7.15 (d, 1H, *J =* 8.0 Hz), 7.22 (d, 1H, *J =* 16.0 Hz), 7.32 (s, 1H), 7.59 (d, 1H, *J =* 16.0 Hz), 7.66 (d, 1H, *J =* 16.0 Hz), 7.89 (d, 1H, *J =* 4.5 Hz), 8.62–8.69 (m, 1H), 8.77 (d, 1H, *J =* 3.5 Hz), 9.13 (s, 1H), 9.80 (bs, 1H); ^13 ^C NMR (125 MHz, DMSO-d_6_) δ 56.23, 102.9, 111.9, 116.2, 121.6, 124.3, 126.5, 126.7, 129.2, 133.8, 141.0, 143.1, 144.4, 144.8, 148.5, 150.4, 179.2, 187.2. HRMS (EI) *m/z*: [M]^+^ calcd. for C_19_H_17_NO_4_ 323.1158; Found: 323.1156.

**5-Hydroxy-7-(4-hydroxy-3-methoxyphenyl)-1-phenylhepta-1,4,6-trien-3-one (5h):** Yield: 45 mg (11%); red brown solid; mp: 135–137 °C (Lit^36^. 137–140); ^1^H NMR (500 MHz, Acetone-d_6_) δ 3.90 (s, 3H), 6.02 (s, 1H), 6.83 (d, 1H, *J =* 21.5 Hz), 6.86 (d, 1H, *J =* 13.0 Hz), 7.17 (dd, 1H, *J*_1_ *=* 8.2 Hz, *J*_2_ = 1.7 Hz), 7.33 (d, 1H, *J =* 2.5 Hz), 7.38–7.43 (m, 3H), 7.52 (d, 1H, *J =* 16.0 Hz), 7.63 (d, 1H, *J =* 8.5 Hz), 7.65 (d, 1H, *J =* 1.5 Hz), 7.67 (s, 1H), 8.18 (bs, 1H); ^13 ^C NMR (125 MHz, Acetone-d_6_) δ 55.48, 101.3, 110.7, 115.4, 121.4, 123.2, 124.3, 127.2, 128.1, 129.0, 130.0, 135.3, 139.6, 141.2, 147.9, 149.3, 182.1. HRMS (EI) *m/z*: [M]^+^ calcd. for C_20_H_18_O_4_ 322.1205; Found: 322.1205.

**Methyl 4-(4-(5-hydroxy-7–(4-hydroxy-3-methoxyphenyl)-3-oxohepta-1,4,6-trien-1-yl)-2-methoxyphenoxy)-butanoate (5i):** Yield: 48 mg (8%); yellow solid; mp: 100–101 °C; ^1^H NMR (500 MHz, Acetone-d_6_) δ 2.04–2.09 (m, 2H), 2.51 (t, 2H, *J =* 7.2 Hz), 3.61 (s, 3H), 3.85 (s, 3H), 3.88 (s, 3H), 4.07 (t, 2H, *J =* 6.0 Hz), 5.96 (s, 1H), 6.70 (t, 2H, *J =* 15.7 Hz), 6.85 (d, 1H, *J =* 8.0 Hz), 6.97 (d, 1H, *J =* 7.5 Hz), 7.15 (d, 1H, *J =* 9.0 Hz), 7.19 (d, 1H, *J =* 8.0 Hz), 7.30 (s, 2H), 7.57 (d, 1H, *J =* 16.0 Hz), 8.29 (bs, 1H); ^13 ^C NMR (125 MHz, Acetone-d_6_) δ 24.58, 50.87, 55.37, 55.46, 67.58, 100.9, 110.6(6), 110.6(9), 112.9, 115.4, 121.4, 122.0, 122.7, 123.0, 127.2, 128.3, 140.2, 140.7, 147.9, 149.3, 149.9, 150.7, 172.9, 183.2, 184.0. HRMS (EI) *m/z*: [M]^+^ calcd. for C_26_H_28_O_8_ 468.1784; Found: 468.1784.

**5-Hydroxy-7-(4-hydroxy-3-methoxyphenyl)-1-(4-nitrophenyl)hepta-1,4,6-trien-3-one (5j):** Yield: 249 mg (53%); orange solid; mp: 204–205 °C; ^1^H NMR (500 MHz, Acetone-d_6_) δ 3.89 (s, 3H), 6.10 (s, 1H), 6.76 (d, 1H, *J =* 16.0 Hz), 6.87 (d, 1H, *J =* 8.5 Hz), 7.05 (d, 1H, *J =* 16.0 Hz), 7.19 (dd, 2H, *J*_1_ *=* 8.2 Hz, *J*_2_ = 2.2 Hz), 7.35 (d, 1H, *J =* 1.5 Hz), 7.65 (d, 1H, *J =* 16.0 Hz), 7.69 (d, 1H, *J =* 16.0 Hz), 7.93–7.96 (m, 2H), 8.25–8.28 (m, 2H), 8.31 (bs, 1H); ^13 ^C NMR (125 MHz, Acetone-d_6_) δ 55.48, 102.2, 110.7, 115.3, 121.6, 123.5, 124.0, 127.0, 128.2, 128.9, 136.3, 141.7, 142.1, 149.9, 148.2, 149.5, 178.8, 186.6. HRMS (EI) *m/z*: [M]^+^ calcd. for C_20_H_17_NO_6_ 367.1056; Found: 367.1056.

**5-Hydroxy-7-(4-hydroxy-3-methoxyphenyl)-1-(4-(trifluoromethyl)phenyl)hepta-1,4,6-trien-3-one (5k):** Yield: 114 mg (23%); yellow solid; mp: 158–160 °C; ^1^H NMR (500 MHz, Acetone-d_6_) δ 3.89 (s, 3H), 6.07 (s, 1H), 6.75 (d, 1H, *J =* 16.5 Hz), 6.86 (d, 1H, *J =* 7.5 Hz), 6.98 (d, 1H, *J =* 16.0 Hz), 7.18 (dd, 1H, *J*_1_ *=* 8.0 Hz, *J*_2_ = 1.5 Hz), 7.34 (d, 1H, *J =* 1.5 Hz), 7.63 (d, 1H, *J =* 10.5 Hz), 7.66 (d, 1H, *J =* 10.5 Hz), 7.75 (d, 1H, *J =* 7.5 Hz), 7.89 (d, 1H, *J =* 7.5 Hz), 8.35 (bs, 1H); ^13 ^C NMR (125 MHz, Acetone-d_6_) δ 55.51, 101.8, 110.8, 115.3, 121.5, 123.4, 125.8, 126.9, 127.1, 128.5, 130.4, 137.3, 139.2, 141.8, 147.9, 149.4, 179.7, 185.9. HRMS (EI) *m/z*: [M]^+^ calcd. for C_21_H_17_F_3_O_4_ 390.1079; Found: 390.1080.

**1-(4-Fluorophenyl)-5-hydroxy-7-(4-hydroxy-3-methoxyphenyl)hepta-1,4,6-trien-3-one (5l):** Yield: 231 mg (53%); orange solid; mp: 146–147 °C; ^1^H NMR (500 MHz, Acetone-d_6_) δ 3.89 (s, 3H), 6.00 (s, 1H), 6.72 (d, 1H, *J =* 16.0 Hz), 6.79 (d, 1H, *J =* 16.0 Hz), 6.86 (d, 1H, *J =* 8.5 Hz), 7.16–7.20 (m, 3H), 7.33 (d, 1H, *J =* 2.5 Hz), 7.59 (d, 1H, *J =* 3.0 Hz), 7.62 (d, 1H, *J =* 3.5 Hz), 7.73 (d, 1H, *J =* 5.0 Hz), 7.74 (d, 1H, *J =* 5.0 Hz), 8.27 (bs, 1H); ^13 ^C NMR (125 MHz, Acetone-d_6_) δ 55.48, 110.7, 115.3, 115.8, 116.0, 121.4, 123.2, 124.2, 127.2, 130.1, 130.2, 130.3, 131.8, 138.2, 141.2, 147.9, 149.2, 162.7, 164.6, 181.4, 184.5. HRMS (EI) *m/z*: [M]^+^ calcd. for C_20_H_17_FO_4_ 340.1111; Found: 340.1115.

**1-(4-Bromophenyl)-5-hydroxy-7-(4-hydroxy-3-methoxyphenyl)hepta-1,4,6-trien-3-one (5m):** Yield: 164 mg (32%); yellow solid; mp: 148–149 °C; ^1^H NMR (500 MHz, Acetone-d_6_) δ 3.89 (s, 3H), 6.02 (s, 1H), 6.73 (dd, 1H, *J*_1_ *=* 16.2 Hz, *J*_2_ = 2.2 Hz), 6.85–6.88 (m, 2H), 7.17 (d, 1H, *J =* 7.5 Hz), 7.33 (s, 1H), 7.56–7.63 (m, 6H), 8.28 (bs, 1H); ^13 ^C NMR (125 MHz, Acetone-d_6_) δ 55.48, 110.7, 115.3, 110.7, 115.3, 121.5, 123.3, 123.5, 125.1, 127.2, 129.8, 132.1, 134.6, 138.0, 141.4, 147.9, 149.3, 180.7, 185.1. HRMS (EI) *m/z*: [M]^+^ calcd. for C_20_H_17_BrO_4_ 400.0310; Found: 430.0309.

***N*-(4–(5-Hydroxy-7–(4-hydroxy-3-methoxyphenyl)-3-oxohepta-1,4,6-trien-1-yl)phenyl)acetamide (5n):** Yield: 281 mg (58%); yellow solid; mp: 183–185 °C; ^1^H NMR (500 MHz, Acetone-d_6_) δ 2.07 (s, 3H), 3.89 (s, 3H), 5.98 (s, 1H), 6.69 (d, 1H, *J =* 10.5 Hz), 6.73 (d, 1H, *J =* 10.5 Hz), 6.86 (d, 1H, *J =* 8.5 Hz), 7.16 (dd, 1H, *J*_1_ *=* 7.7 Hz, *J*_2_ = 1.7 Hz), 7.32 (d, 1H, *J =* 2.5 Hz), 7.57–7.61 (m, 4H), 7.68 (s, 1H), 7.70 (s, 1H), 8.15 (bs, 1H); ^13 ^C NMR (125 MHz, Acetone-d_6_) δ 23.58, 23.62, 55.56, 101.2, 110.7, 115.4, 119.1, 119.2, 121.5, 122.7, 123.2, 127.3, 129.0, 130.1, 139.6, 141.0, 141.4, 141.5, 148.0, 149.3, 168.3, 168.4, 182.8, 184.3. HRMS (EI) *m/z*: [M]^+^ calcd. for C_22_H_21_NO_5_ 379.1420; Found: 379.1419.

**5-Hydroxy-7-(4-hydroxy-3-methoxyphenyl)-1-(4-methoxyphenyl)hepta-1,4,6-trien-3-one (5o):** Yield: 320 mg (66%); yellow solid; mp: 143–145 °C; ^1^H NMR (500 MHz, Acetone-d_6_) δ 3.01 (s, 6H), 3.89 (s, 3H), 5.90 (s, 1H), 6.55 (d, 1H, *J =* 15.0 Hz), 6.67 (d, 1H, *J =* 16.0 Hz), 6.73 (d, 2H, *J =* 9.0 Hz), 6.85 (d, 1H, *J =* 7.5 Hz), 7.14 (dd, 1H, *J*_1_ *=* 8.5 Hz, *J*_2_ = 1.5 Hz), 7.31 (d, 1H, *J =* 2.0 Hz), 7.50 (d, 1H, *J =* 9.0 Hz), 7.55 (d, 1H, *J =* 1.5 Hz), 7.58 (s, 1H), 8.19 (bs, 1H); ^13 ^C NMR (125 MHz, Acetone-d_6_) δ 54.96, 55.46, 101.0, 110.6, 114.4, 115.4, 121.4, 121.9, 123.1, 127.3, 127.8, 129.9, 139.8, 140.7, 147.9, 149.2, 161.5, 183.3, 184.0. HRMS (EI) *m/z*: [M]^+^ calcd. for C_21_H_20_O_5_ 352.1311; Found: 352.1314.

**5-Hydroxy-7-(4-hydroxy-3-methoxyphenyl)-1-(4-hydroxyphenyl)hepta-1,4,6-trien-3-one (5p):** Yield: 281 mg (65%); orange solid; mp: 169–170 °C (Lit^37^. 170–172 °C); ^1^H NMR (500 MHz, Acetone-d_6_) δ 3.89 (s, 3H), 5.94 (s, 1H), 6.64 (d, 1H, *J =* 16.0 Hz), 6.69 (d, 1H, *J =* 15.5 Hz), 6.84–6.88 (m, 3H), 7.15 (dd, 1H, *J*_1_ *=* 8.5 Hz, *J*_2_ = 2.5 Hz), 7.31 (d, 1H, *J =* 1.5 Hz), 7.53–7.56 (m, 3H), 7.59 (d, 1H, *J =* 3.5 Hz), 8.21 (bs, 1H); ^13 ^C NMR (125 MHz, Acetone-d_6_) δ 55.45, 101.9, 110.5, 115.4, 115.9, 121.2, 121.4, 123.0, 126.8, 127.3, 130.1, 140.2, 140.5, 147.9, 149.2, 159.7, 183.6. HRMS (EI) *m/z*: [M]^+^ calcd. for C_20_H_18_O_5_ 338.1154; Found: 338.1159.

**1-(4-(Dimethylamino)phenyl)-5-hydroxy-7-(4-hydroxy-3-methoxyphenyl)hepta-1,4,6-trien-3-one (5r):** Yield: 220 mg (48%); red solid; mp: 174–175 °C; ^1^H NMR (500 MHz, Acetone-d_6_) δ 3.01 (s, 6H), 3.89 (s, 3H), 5.90 (s, 1H), 6.55 (d, 1H, *J =* 15.0 Hz), 6.67 (d, 1H, *J =* 16.0 Hz), 6.73 (d, 2H, *J =* 9.0 Hz), 6.82 (d, 1H, *J =* 7.5 Hz), 7.14 (dd, 1H, *J*_1_ *=* 8.5 Hz, *J*_2_ = 1.5 Hz), 7.31 (d, 1H, *J =* 2.0 Hz), 7.50 (d, 2H, *J =* 9.0 Hz), 7.55 (d, 1H, *J =* 1.5 Hz), 7.58 (s, 1H), 8.19 (bs, 1H); ^13 ^C NMR (125 MHz, Acetone-d_6_) δ 39.36, 55.52, 100.8, 110.6, 111.9, 115.3, 118.7, 121.5, 122.8, 127.4, 130.0, 139.8, 141.3, 147.8, 148.8, 152.0, 182.0, 184.4. HRMS (EI) *m/z*: [M]^+^ calcd. for C_22_H_23_NO_4_ 365.1627; Found: 365.1624.

**5-Hydroxy-7-(4-hydroxy-3-methoxyphenyl)-1-(4-(piperidin-1-yl)phenyl)hepta-1,4,6-trien-3-one (5s):** Yield: 56 mg (12%); red solid; mp: 204–205 °C; ^1^H NMR (500 MHz, Acetone-d_6_) δ 1.61–1.63 (m, 6H), 3.22–3.31 (m, 4H), 3.89 (s, 3H), 5.92 (s, 1H), 6.58 (d, 1H, *J =* 15.5 Hz), 6.67 (d, 1H, *J =* 15.5 Hz), 6.85 (d, 1H, *J =* 8.0 Hz), 6.93 (d, 2H, *J =* 9.5 Hz), 7.14 (dd, 1H, *J*_1_ *=* 8.2 Hz, *J*_2_ = 1.7 Hz), 7.31 (d, 1H, *J =* 2.0 Hz), 7.50 (d, 2H, *J =* 9.5 Hz), 7.54 (d, 1H, *J =* 5.0 Hz), 7.57 (d, 1H, *J =* 4.5 Hz), 8.19 (bs, 1H); ^13 ^C NMR (125 MHz, Acetone-d_6_) δ 24.22, 25.36, 48.70, 55.46, 100.81, 110.58, 114.7, 115.3, 119.7, 121.5, 122.9, 124.3, 127.4, 129.8, 140.1, 140.7, 147.9, 149.1, 153.0, 182.9, 184.3. HRMS (EI) *m/z*: [M]^+^ calcd. for C_25_H_27_NO_4_ 405.1940; Found: 405.1943.

#### 2.6.2 Synthesis of 1,7-bis(3,4-dimethoxyphenyl)hepta-1,6-diene-3,5-dione (4c)

Curcumin (1.0 g, 2.71 mmol) was dissolved in dry acetone (30 ml) then anhydrous K_2_CO_3_ (1.12 g, 8.14 mmol) and CH_3_I (1.69 ml, 27.14 mmol) were added. The reaction mixture was refluxed for 24 h and monitored by TLC. The reaction mixture was then cooled to ambient temperature and filtrated. The resulting filtrate was evaporated under reduced pressure and ethyl acetate (30 ml) and water (20 ml) were added. The organic layer was washed with water and brine, and then dried over anhydrous MgSO_4_. After evaporating under reduced pressure, the residue was purified using a Cosmosil 140 C-18 OPN column (CH_3_CN:H_2_O = 3:2 (v/v)) to yield 1,7-bis(3, 4-dimethoxyphenyl)hepta-1,6-diene-3,5-dione (**4c**, 150 mg, 14%) and 1-(3,4-dimethoxyphenyl)-7-(4-hydroxy-3-methoxyphenyl)hepta-1,6-diene-3,5-dione (**5a**, 210 mg, 20%) as orange solids.

**1,7-Bis(3,4-dimethoxyphenyl)-5-hydroxyhepta-1,4,6-trien-3-one (4c):** Yield: 150 mg (14%); orange solid; mp: 134–136 °C (Lit^38^. 132–133 °C); ^1^H NMR (500 MHz, Acetone-d_6_) δ 3.84 (s, 6H), 3.85 (s, 6H), 5.97 (s, 1H), 6.70 (s, 1H), 6.73 (s, 1H), 6.97 (s, 1H), 6.98 (s, 1H), 7.11 (s, 1H), 7.21 (s, 1H), 7.29 (s, 2H), 7.57 (s, 1H), 7.60 (s, 1H); ^13 ^C NMR (125 MHz, Acetone-d_6_) δ 55.30, 101.2, 110.4, 111.6, 122.0, 122.8, 123.0, 123.7, 128.1, 140.4, 142.8, 149.8, 151.6, 183.6. HRMS (EI) *m/z*: [M]^+^ calcd. for C_23_H_24_O_6_ 396.1573; Found: 396.1572.

#### 2.6.3 Synthesis of 1,7-bis(3,4-dihydroxyphenyl)hepta-1,6-diene-3,5-dione (4d)^34^

Curcumin (0.3 g, 0.81 mmol) was suspended in dry CH_2_Cl_2_ (30 ml), then stirred for 10 min at –20 °C under N_2_ (g) atmosphere. Tribromoborane (0.25 ml ×5 times) was slowly added. The reaction mixture was allowed to warm up to ambient temperature and stirred overnight. Therefore, the reaction mixture was slowly poured into the saturated NaHCO_3_ solution (30 ml) with ice powder, and then stirred for 2 h. The water layer was separated and acidified with 1 M HCl (aqueous), then extracted with ethyl acetate (30 ml ×3 times). The organic layer was washed with water and brine, and then dried over anhydrous MgSO_4_. After evaporating the solvent under reduced pressure, the residue was applied to the top of an open-bed silica gel column (*n*-hexane: ethyl acetate: MeOH =60:38:2 (v/v)) to yield 1,7-bis(3,4-dihydroxyphenyl)hepta-1,6-diene-3,5-dione (**4d**, 27 mg, 10%) as an orange amorphous solid.

**1,7-bis(3,4-dihydroxyphenyl)-5-hydroxyhepta-1,4,6-trien-3-one (4d):** Yield: 27 mg (10%); orange solid; mp: 300 °C (decomposed, Lit^39^. 304–305); ^1^H NMR (500 MHz, Acetone-d_6_) δ 5.95 (d, 1H, *J =* 6.0 Hz), 6.58 (dd, 2H, *J*_1_ *=* 15.5 Hz, *J*_2_ = 6.0 Hz), 6.85 (s, 2H), 7.04 (s, 2H), 7.16 (d, 2H, *J =* 3.0 Hz), 7.51 (dd, 2H, *J*_1_ *=* 15.2 Hz, *J*_2_ = 6.2 Hz), 8.39 (bs, 4H); ^13 ^C NMR (125 MHz, Acetone-d_6_) δ 100.9, 114.4, 115.6, 121.2, 121.8, 127.5, 140.5, 145.5, 147.9, 183.5. HRMS (EI) *m/z*: [M]^+^ calcd. for C_19_H_16_O_6_ 340.0947; Found: 340.0949.

#### 2.6.4 Synthesis of 2–(3-hydroxy-4-methoxybenzylidene)-6–(3-(3-hydroxy-4-methoxyphenyl)acryloyl)cyclohexa-n-1-one (4f)

2-Acetyl-cyclohexan-1-one (0.3 g, 2.14 mmol), boron trioxide (0.14 g, 2.14 mmol), morpholine (0.037 ml, 0.42 mmol), acetic acid (0.024 ml, 0.42 mmol), and vanillin (0.651 g, 4.28 mmol) were placed in a capped vial without solvent at ambient temperature. The resulting mixture was irradiated in a microwave oven (300 W output, 1378 KPa) at 150 °C for 40 min until the 2-acetyl-cyclohexan-1-one or vanillin was consumed. The reaction was monitored by TLC. After the reaction vial cooled, the product was dissolved in MeOH. The reaction mixture was evaporated under reduced pressure, then purified further by silica gel column chromatography (*n*-hexane: ethyl acetate: MeOH =60:38:2 (v/v)) to give 2-(3-hydroxy-4-methoxybenzylidene)-6-(3-(3-hydroxy-4-methoxyphenyl)acryloyl)cyclohexan-1-one (**4f**, 390 mg, 45%) as an orange red solid.

**2–(1-Hydroxy-3-(4-hydroxy-3-methoxyphenyl)allylidene)-6-(4-hydroxy-3-methoxybenzylidene)cyclohexan-1-one (4f):** Yield: 390 mg (45%); orange red solid; mp: 182–183 °C (Lit^40^. 175–176 °C); ^1^H NMR (500 MHz, MeOH-d_4_) δ 1.75**–**1.80 (m, 2H), 2.67 (t, 2H, *J =* 6.2 Hz), 2.77 (t, 2H, *J =* 5.5 Hz), 3.86 (s, 3H), 3.90 (s, 3H), 6.80 (d, 1H, *J =* 3.5 Hz), 6.82 (d, 1H, *J =* 4.0 Hz), 6.95 (dd, 1H, *J*_1_ *=* 8.5 Hz, *J*_2_ = 1.5 Hz), 7.03 (d, 1H, *J =* 1.5 Hz), 7.07 (d, 1H, *J =* 15 Hz), 7.13 (dd, 1H, *J*_1_ *=* 8.2 Hz, *J*_2_ = 2.2 Hz), 7.22 (d, 1H, *J =* 2.5 Hz), 7.58 (s, 1H), 7.63 (d, 1H, *J =* 15.5 Hz); ^13 ^C NMR (125 MHz, MeOH-d_4_) δ 22.90, 24.03, 27.08, 55.06, 108.3, 110.6, 113.5, 114.8, 115.2, 117.3, 122.9, 123.8, 127.4, 128.2, 130.6, 133.2, 142.5, 147.1, 147.4, 148.0, 149.2, 177.6, 185.7. HRMS (EI) *m/z*: [M]^+^ calcd. for C_24_H_24_O_6_ 408.1573; Found: 408.1572.

#### 2.6.5 Synthesis of 1-(4-aminophenyl)-7-(4-hydroxy-3-methoxyphenyl)hepta-1, 6-diene-3,5-dione (5q)

Compound **5n** (100 mg, 0.26 mmol) was dissolved in tetrahydrofuran (5 ml), then ethanol (20 ml) and 1 M HCl (aq., 20 ml) were added. The reaction mixture was stirred at reflux for 22 h and monitored by TLC, then cooled to ambient temperature and evaporated under reduced pressure. Ethyl acetate (20 ml) was added to the residue and the mixture was neutralised using NaHCO_3_ (aq, sat.). The organic phase was washed with water (20 ml ×3 times) and brine, dried over MgSO_4_, and the solvent was evaporated under reduced pressure. The residue was applied to the top of an open-bed silica gel column (*n*-hexane: ethyl acetate: methanol =60:35:5 (v/v)) to yield 1-(4-aminophenyl)-7-(4-hydroxy-3-methoxyphenyl)hepta-1,6-diene-3,5-dione (**5q**, 75 mg, 85%) as a red solid.

**1-(4-Aminophenyl)-5-hydroxy-7-(4-hydroxy-3-methoxyphenyl)hepta-1,4,6-trien-3-one (5q):** Yield: 75 mg (85%); red solid; mp: 99–100 °C; ^1^H NMR (500 MHz, Acetone-d_6_) δ 3.89 (s, 3H), 5.29 (bs, 2H), 5.89 (s, 1H), 6.51 (d, 1H, *J =* 15.5 Hz), 6.65–6.68 (m, 3H), 6.85 (d, 1H, *J =* 8.5 Hz), 7.13 (dd, 1H, *J*_1_ *=* 8.0 Hz, *J*_2_ = 2.0 Hz), 7.30 (d, 1H, *J =* 2.0 Hz), 7.39 (d, 2H, *J =* 9.5 Hz), 7.52 (d, 1H, *J =* 2.5 Hz), 7.55 (d, 1H, *J =* 2.5 Hz), 8.22 (bs, 1H); ^13 ^C NMR (125 MHz, Acetone-d_6_) δ 55.46, 100.6, 110.5, 114.2, 115.3, 118.6, 120.0, 121.4, 122.9, 123.4, 127.4, 129.2, 130.2, 139.9, 141.4, 147.9, 149.0, 151.3, 182.4, 184.8. HRMS (EI) *m/z*: [M]^+^ calcd. for C_20_H_19_NO_4_ 337.1314; Found: 337.1316.

#### 2.6.6 Synthesis of 1,7-bis(3-hydroxy-4-methoxyphenyl)heptane-3,5-dione (6)

Pd-C (10%, 50 mg) was added to a solution of curcumin (0.5 g, 0.27 mmol) in methanol (10 ml). After degassing, the mixture was stirred at 0 °C for 10 min, and then hydrogenated in a hydrogen atmosphere (balloon) for 12 h. The mixture was filtered through a Celite 545 and the solvent was evaporated under reduced pressure. The residue was applied to the top of an open-bed silica gel column (CH_2_Cl_2_:MeOH gradient elution) to yield 1,7-bis(3-hydroxy-4-methoxyphenyl)heptane-3,5-dione (**6**, 140 mg, 28%) as a white solid.

**1,7-Bis(4-hydroxy-3-methoxyphenyl)heptane-3,5-dione (6):** Yield: 140 mg (28%); white solid; mp: 94–96 °C (Lit^41^. 95–97 °C); ^1^H NMR (500 MHz, Acetone-d_6_) δ 2.55 (t, 2H, *J*_1_ *=* 7.5 Hz, *J*_2_ = 8.5 Hz), 2.76–2.80 (m, 6H), 3.78 (s, 6H), 5.62 (s, 1H), 6.59–6.64 (m, 2H), 6.68–6.70 (m, 2H), 6.78 (d, 1H, *J =* 1.5 Hz), 6.81 (d, 1H, *J =* 1.5 Hz); ^13 ^C NMR (125 MHz, Acetone-d_6_) δ 30.99, 40.03, 45.07, 55.34, 99.49, 111.8, 114.8, 120.6, 132.2, 144.9, 147.5, 197.6. HRMS (EI) *m/z*: [M]^+^ calcd. for C_21_H_24_O_6_ 372.1573; Found: 372.1574.

#### 2.6.7 Synthesis of (3,5-dioxohepta-1,6-diene-1,7-diyl)bis(2-methoxy-1,4-phenylene) diacetate (7)

*N*,*N*-dimethylaminopyridine (0.13 g, 1.08 mmol) was added to a solution of curcumin (2 g, 5.42 mmol) in anhydrous pyridine (10 ml) at ambient temperature. Acetic anhydride (1.02 ml, 10.8 mmol) was dropped into the reaction mixture in a 0 °C ice bath, and then stirred overnight at ambient temperature. The reaction mixture was then poured into crashed ice and extracted with EtOAc (3 × 30 ml). The organic layer was washed with water, dried over anhydrous Na_2_SO_4_, and the solvent was evaporated under reduced pressure. The crude product was purified by flash column chromatography (CH_2_Cl_2_) to yield di-(3,5-dioxohepta-1,6-diene-1,7-diyl)bis(2-methoxy-1,4-phenylene) diacetate^7^ (1.9 g, 77%) as a yellow solid.

**3-Hydroxy-5-oxohepta-1,3,6-triene-1,7-diyl)bis(2-methoxy-4,1-phenylene) diacetate (7):** Yield: 1.9 g (77%); yellow solid; mp: 158–159 °C (Lit^38^. 156–158); ^1^H NMR (500 MHz, Acetone-d_6_) δ 2.23 (s, 6H), 3.86 (s, 6H), 6.04 (s, 1H), 6.82 (s, 1H), 6.86 (s, 1H), 7.08 (s, 1H), 7.10 (s, 1H), 7.24 (d, 1H, *J =* 2.5 Hz), 7.26 (d, 1H, *J =* 1.5 Hz), 7.41 (d, 2H, *J =* 2.0 Hz); ^13 ^C NMR (125 MHz, Acetone-d_6_) δ 19.65, 55.54, 101.6, 111.7, 121.1, 123.3, 124.4, 134.0, 139.8, 141.7, 151.8, 168.0, 183.6. HRMS (EI) *m/z*: [M]^+^ calcd. for C_25_H_24_O_8_ 352.1472; Found: 352.1472.

#### 2.6.8 Synthesis of 4,4'-((1*H*-pyrazole-3,5-diyl)bis(ethene-1,2-diyl))bis(2-methoxyphenol) (8)

Hydrazine hydrate (0.07 ml, 0.81 mmol) was added to a solution of curcumin (0.2 g, 0.54 mmol) in glacial acetic acid at room temperature, then the mixture was stirred for 2 h under reflux and the solvent was evaporated under reduced pressure. EtOAc (30 ml) was added to the residue and the organic layer was washed with water and brine, and then dried over anhydrous MgSO_4_. After the solvent was evaporated under reduced pressure, the residue was applied to the top of an open-bed silica gel column (CH_2_Cl_2_: MeOH =95:5 (v/v)) to yield 4,4'-((1*H*-pyrazole-3, 5-diyl)bis(ethene-1,2-diyl))bis(2-methoxyphenol) (**8,** 130 mg, 68%) as an ivory solid.

**4,4'-(1*H*-Pyrazole-3,5-diyl)bis(ethene-1,2-diyl))bis(2-methoxyphenol) (8):** Yield: 130 mg (68%); ivory solid; mp: 220–222 °C (Lit. 218 °C); ^1^H NMR (500 MHz, Acetone-d_6_) δ 3.86 (s, 6H), 6.65 (s, 1H), 6.79 (s, 1H), 6.81 (s, 1H), 6.96 (d, 1H, *J =* 3.0 Hz), 6.98 (d, 1H, *J =* 1.5 Hz), 7.00 (s, 1H), 7.09 (s, 1H), 7.12 (s, 1H), 7.17 (d, 2H, *J =* 2.5 Hz), 8.21 (bs, 1H); ^13 ^C NMR (125 MHz, Acetone-d_6_) δ 55.38, 99.21, 109.0, 115.2, 115.8, 120.3, 129.2, 129.9, 146.9, 147.1, 147.8. HRMS (EI) *m/z*: [M]^+^ calcd. for C_21_H_20_N_2_O_4_ 364.1423; Found: 364.1423.

## 3. Results and discussion

### 3.1 Chemistry

**Scheme 2.**
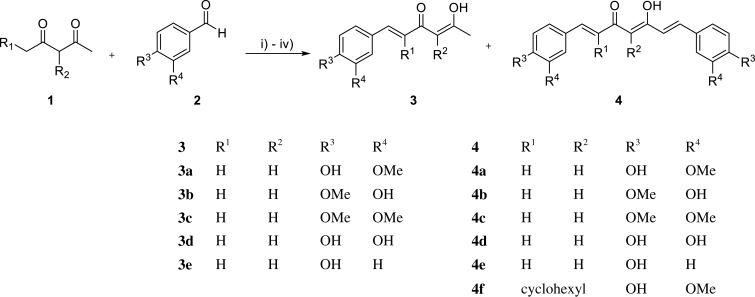
Reagent and conditions for synthesis of **3:** (i) **1**, B_2_O_3_, ethyl acetate, 90 °C; (ii) **2**, *n*-(BuO)_3_B, ethyl acetate, 90 °C; (iii) *n*-BuNH_2_, ethyl acetate, 90 °C; (iv) hydrochloric acid (1M, aq.), 50 °C; synthesis of **4**: (i) **3**, B_2_O_3_, ethyl acetate, 90 °C; (ii) **2**, *n*-(BuO)_3_B, ethyl acetate, 90 °C; (iii) *n*-BuNH_2_, ethyl acetate, 90 °C; (iv) hydrochloric acid (1M, aq.), 50 °C; synthesis of **4f**: **1**, **2**, B_2_O_3_, morpholine, AcOH, 40 min, microwave irradiation (300 W).

**Scheme 3.**
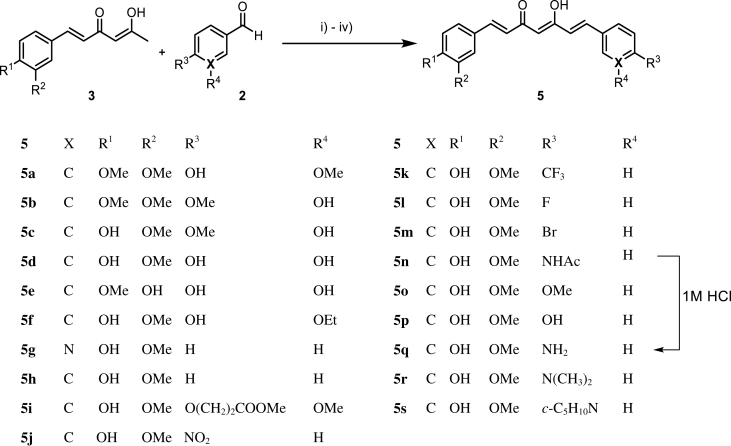
Reagent and conditions for synthesis of asymmetrical curcumin derivatives **5**: (i) **3**, B_2_O_3_, ethyl acetate, 90 °C; (ii) **2**, *n*-(BuO)_3_B, ethyl acetate, 90 °C; (iii) *n*-BuNH_2_, ethyl acetate, 90 °C; (iv) hydrochloric acid (1M, aq.), 50 °C.

**Scheme 4.**
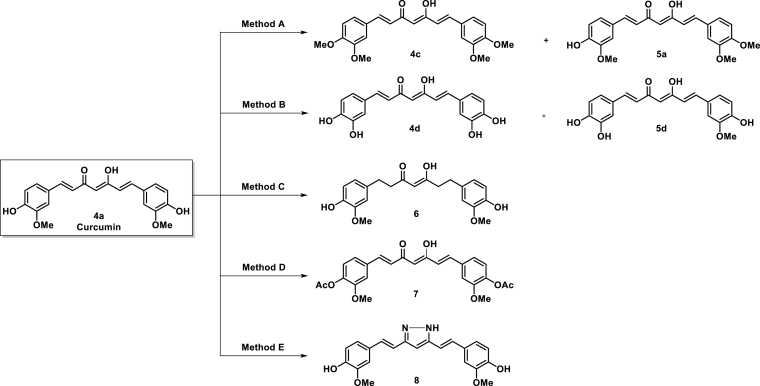
Reagent and conditions for synthesis of curcumin derivatives **4c**, **4d**, and **6**–**8**: (A) **4a**, CH_3_I, K_2_CO_3_, acetone, reflux; (B): **4a**, BBr_3_, –78 °C to ambient temperature, N_2_ (g), H_2_O; (C) **4a**, H_2_/Pd-C (10 wt. % of palladium), MeOH, 0 °C to ambient temperature; (D) **4a**, Ac_2_O, DMAP, pyridine, ambient temperature; (E) **4a**, NH_2_NH_2_^.^H_2_O, AcOH, reflux.

The synthetic route of the curcumin derivatives is shown in Schemes 2–4. The 6-(3, 4-substituted-phenyl)-hex-5-ene-2,4-diones (**3**) were prepared using acetylacetone with 1 equivalent of corresponding aldehydes by Pabon’s method. From this reaction, the symmetrical curcumins, **4a–4e**, were obtained as by-products (Scheme 2)^39^. The symmetrical curcumin derivatives, **4**, were synthesised by combining **1** with 2 equivalents of corresponding aldehydes **2**, except of compounds **4c** and **4d** owing to trace yields. The synthesis of **4c** and **4d** is shown in Scheme 4. Compound **4f**, which has an inserted cyclohexyl group in 1,3-diketone, was prepared by combining 2-acetylcyclohexanone with vanillin under microwave irradiation^40^.

Twenty asymmetrical curcumin derivatives, **5**, were prepared using Pabon’s reaction with **3** and the corresponding aldehydes (Scheme 3)^39^. Compound **5q** was synthesised by deacetylation of **5n** using 1 M HCl aqueous solution.

Symmetrical curcumin derivatives **4c**, **4d**, and **6**–**8** were prepared by treating the corresponding reagent with curcumin (Scheme 4). Compound **4c** was prepared with MeI/K_2_CO_3_ in acetone under reflux conditions and compound **4d** was produced by a demethylation reaction after treating tribromoborane with dry dichloromethane^41^. Tetrahydrocurcumin (**6**) was prepared by hydrogenation using 10% Pd-C as a catalyst^42^ and acetylated curcumin **7** was prepared using acetic anhydride with DMAP as a catalyst with anhydrous pyridine^34^. Compound **8** was prepared by condensation the 1, 3-diketone in curcumin with hydrazine hydrate under reflux condition^28^. The structures of all compounds were characterised using ^1^H NMR, ^13 ^C NMR, and EI-HRMS.

### 3.2 Biological evaluation

Hydrolytic activity of *S. pneumoniae* Nan A was confirmed using DANA (Neu5Ac2en). The IC_50_ value of DANA with respect to *S. pneumoniae* sialidase inhibition was 4.8 ± 1.1 µM^12^. To identify a sialidase inhibitor of *S. pneumoniae*, the inhibitory activity of *Turmeric* ethanol extract and its three major components was compared. For the methanol extract, the Nan A activity was 88% at 30 µg/mL. The sialidase inhibitory activity of the major components of *Turmeric* was as follows; curcumin (**4a**, IC_50_ = 0.6 ± 0.1 µM), demethoxycurcumin (**5p**, IC_50_ = 0.6 ± 0.2 µM), and bisdemthoxycurcumin (**4e**, IC_50_ = 4.0 ± 1.2 µM).

Among these compounds, **4e**, in which two of the methoxy groups were removed from curcumin showed diminished inhibitory activity. Based on this result, we predicted that curcumin derivatives would have inhibitory activity against sialidase from *S. pneumoniae* Nan A and that the methoxy group played an important role in the inhibitory activity. Therefore, we modified curcumin and evaluated the inhibitory effects on *S. pneumoniae* Nan A (Table 1).

**Table 1.** Inhibitory effects of synthesised compounds against *S. pneumoniae* Nan A.

Compounds	IC_50_ (μM)^a^	Compounds	IC_50_ (μM)
**4a**	0.6 ± 0.1	**5i**	1.0 ± 0.5
**4b**	1.0 ± 0.4	**5j**	0.9 ± 0.3
**4c**	3.4 ± 1.0	**5k**	1.0 ± 0.1
**4d**	1.5 ± 0.7	**5l**	1.8 ± 0.2
**4e**	0.4 ± 1.2	**5m**	1.0 ± 0.2
**4f**	4.7 ± 0.5	**5n**	1.8 ± 0.4
**5a**	1.5 ± 0.7	**5o**	1.2 ± 0.1
**5b**	0.6 ± 0.1	**5p**	0.6 ± 0.2
**5c**	1.0 ± 0.4	**5q**	1.6 ± 0.1
**5d**	0.7 ± 0.2	**5r**	1.5 ± 0.2
**5e**	0.2 ± 0.1	**5s**	1.5 ± 0.1
**5f**	1.5 ± 0.8	**6**	82.1 ± 7.6
**5g**	4.4 ± 0.1	**7**	2.6 ± 0.6
**5h**	7.1 ± 0.1	**8**	6.2 ± 1.5

a IC_50_ values of compounds represent the concentration that caused 50% enzyme activity loss; all compounds were examined in a set of triplicates experiment.

To evaluate the functionalisation of the curcumin skeleton to find a suitable backbone. Hydrogenation of the heptadienyl group (**6**, IC_50_ = 82.1 ± 7.6 µM) resulted in significantly diminished inhibitory activity. Next, to confirm the phenyl group at the 7-position, inhibitory activity after the addition of a pyridinyl (**5g**) or phenyl ring (**5h**) was compared. The results indicated that the pyridinyl group (**5 g,** IC_50_ = 4.4 ± 0.1 µM) was more effective than the phenyl group (**5 h**, IC_50_ =7.1 ± 0.1 µM), but both compounds showed lower inhibitory activity than curcumin. To investigate the effect of 1,3-diketone, 4-hydroxy or 3-methoxy groups, synthesised and compared with six kinds of curcumins. First, to investigate the effect of the 1, 3-diketone moiety, pyrazole (**8**, IC_50_ = 6.2 ± 1.5 µM) or carbocyclic 1, 3-diketone (**4f**, IC_50_ = 4.7 ± 0.5 µM) was substituted. Second, to investigate the effect of the 4-hydroxyl groups in curcumin, acetyl (**7**, IC_50_ = 2.6 ± 0.6 µM) or methyl groups (**4c**, IC_50_ = 3.4 ± 1.0 µM) were substituted and conversion of the hydroxy at the *para* position to ester (**5i**, IC_50_ = 1.0 ± 0.5 µM). Third, to investigate the 3-methoxy groups, elimination of the 3-methoxy groups (**4d**, IC_50_ = 1.5 ± 0.7 µM) and conversion of the methoxy at the *meta* position to ethoxy (**5f**, IC_50_ = 1.5 ± 0.8 µM) were compared. Inhibitory activity of these compounds was diminished than curcumin (**4a**). This suggested that 1,3-diketone and a heptadienyl group were essential functional groups for sialidase inhibition and either a methoxy or a hydroxyl group was required.

**Figure 1.**
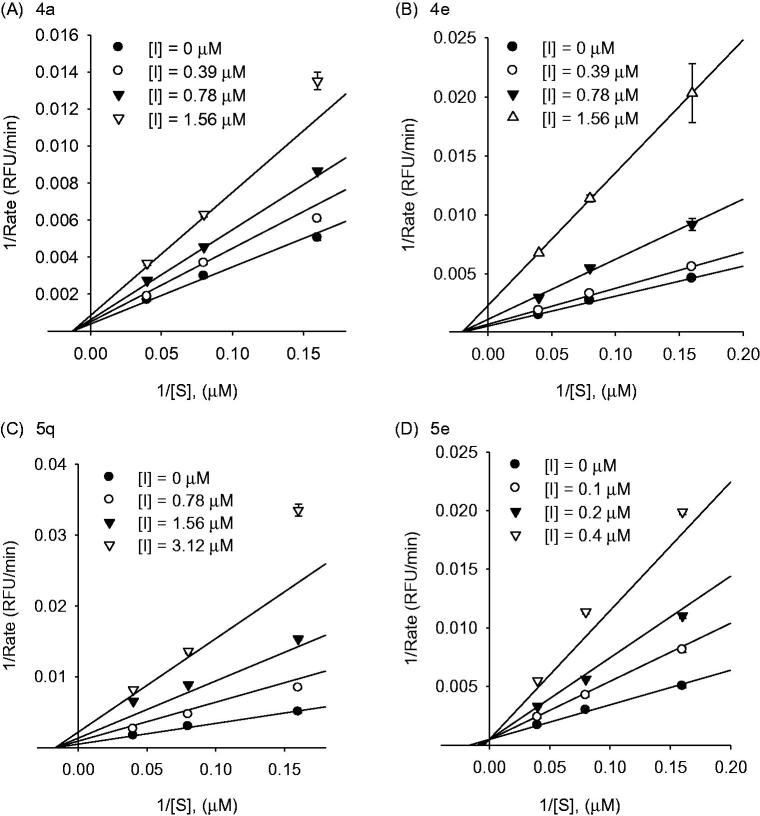
Graphical determination of the inhibition type for compounds **4a**, **4e**, **5q**, and **5e**. Lineweaver–Burk (A–D) plots for the inhibitory activity of compounds **4a**, **4e**, **5q**, and **5e**, respectively, against *S. pneumoniae* Nan A hydrolysis activity in the presence of different substrate concentrations.

Based on these results, we substituted a methoxy or hydroxyl group at the *para*- or *meta*- position of the 1,7-diphenylhepta-1,6-diene-3,5-dione backbone. To confirm the positional tendency of the methoxy and hydroxyl groups, we reacted it with feruloyl (**3a**) or isoferuloyl (**3b**) acetone or hispolon (**3c**) with the corresponding aldehyde **2**. Because a methoxy or hydroxyl group was essential for sialidase inhibition, compounds **4c** and **4d** were excluded from consideration. As shown in Table 1, IC_50_ values ranged from 0.2–1.5 µM, indicating there was no positional tendency for inhibition (**4a**, **4 b,** and **5a–5e** in Table 1). Among the examined compounds, **5e**, containing the catechol with the isoferuloyl moiety, was the most potent inhibitor (IC_50_ = 0.2 ± 0.1 µM), with a 3.0-fold improvement in inhibitory activity over that of curcumin. Thereafter, to investigate the electronic effect, we substituted an electron-donating group (EDG) or electron-withdrawing group (EWG) into the *para* position of the phenyl rings, including nitro (**5j**), trifluoromethyl (**5k**), fluoro (**5l**), bromo (**5m**), acetamido (**5n**), methoxy (**5o**), hydroxyl (**5p**), amino (**5q**), *N*, *N*-dimethylamino (**5r**), and piperidinyl (**5s**) groups. The IC_50_ values ranged from 0.6–1.8 µM and the electronic effect did not influence in Nan A inhibition. Based on these observations, we investigated the kinetic mechanisms of inhibitors with IC_50_ values of 25 µM or less. We selected the major components of *Turmeric* (**4a**, **4e**, **5q**) and compound **5e** as the most potent inhibitors for the kinetic study. We found that the major components of *Turmeric* (**4a**, **4e**, **5q**) showed noncompetitive inhibition characteristics with a *K*_i_ of 1.3 µM (**4a**), 1.2 µM (**4e**), and 0.8 µM (**5q**), respectively. Conversely, compound **5e** exhibited a potent competitive inhibition against Nan A with a *K*_i_ of 0.14 µM (Figure 1).

Synthesised compounds were then evaluated for their inhibitory effect on sialidase from *Vibrio cholerae* and *Clostridium perfringens*, which also release sialidase and play a role in the pathogenesis. The inhibitory assay results are summerised in Table 2.

**Table 2.** Inhibitory effects of curcumin derivatives in *V. cholera* and *C. perfringens* sialidase.

Compound	IC_50_ (μM)^a^		Compound	IC_50_ (μM)	
	*V. cholerae*	*C. perfringens*		*V. cholerae*	*C. perfringens*
**4a**	5.3 ± 0.7	1.6 ± 0.4	**5g**	43.6 ± 0.1	32.9 ± 5.1
**4b**	7.6 ± 0.5	3.2 ± 0.4	**5i**	9.5 ± 1.5	3.3 ± 0.4
**4c**	21.5 ± 7.0	13.1 ± 2.2	**5j**	6.9 ± 1.8	1.6 ± 0.5
**4d**	11.9 ± 2.0	6.1 ± 0.8	**5n**	6.8 ± 0.8	5.9 ± 0.8
**4e**	15.1 ± 0.1	11.0 ± 0.8	**5o**	4.2 ± 0.8	2.8 ± 0.3
**4f**	24.9 ± 1.8	13.4 ± 0.1	**5p**	4.3 ± 0.7	2.1 ± 0.4
**5a**	11.7 ± 0.2	6.7 ± 0.5	**5q**	2.9 ± 0.9	1.7 ± 0.1
**5b**	9.2 ± 0.2	6.7 ± 0.8	**5r**	1.9 ± 0.2	1.3 ± 0.2
**5c**	7.7 ± 0.7	3.5 ± 0.2	**6**	NA^b^	269.4 ± 31.2
**5d**	2.7 ± 0.8	1.0 ± 0.1	**7**	37.5 ± 3.5	13.8 ± 0.6
**5e**	4.8 ± 1.5	0.5 ± 0.07	**8**	41.6 ± 18.7	9.5 ± 0.1
**5f**	6.7 ± 0.6	2.9 ± 0.5			

a IC_50_ values of compounds represent the concentration that caused 50% enzyme activity loss; all compounds were examined in a set of triplicates experiment.

b No activity.

Similar to the above results, methoxy or hydroxyl, heptadienyl and *α*, *β*-unsaturated ketone groups played important roles in the inhibitory activity. Although the position of the methoxy and hydroxyl group did not influence the inhibition of the above enzymes, the inhibitory effect increased with the greater substitution of hydroxyl groups in the phenyl rings. Thereafter, we evaluated inhibitor activity to confirm the electronic effect. *C. perfringens* sialidase inhibitory activity was not affected by the electronic effect. Among the examined compounds, **5e** (IC_50_ = 0.5 ± 0.07 µM), containing the catechol moiety, was the most potent inhibitor of *C. perfringens* and displayed a 3.2-fold improvement over curcumin. Conversely, substitution of an electron donating groups at the *para* position of the phenyl group resulted in better potency than the substitution of an electron withdrawing groups against *V. cholera* sialidase. Among the examined compounds, compound **5r** (IC_50_=1.9 ± 0.2 µM), containing the *N*, *N*-dimethylamino group, was the most potent and displayed a 2.7-fold improvement in inhibitory activity over that of curcumin. The IC_50_ values for sialidase inhibitory activity were 2.7–43.6 µM in *V. Choleara* and 0.5–269.4 µM in *C. perfringens*.

## 4. Conclusions

In conclusion, we developed, for the first time, inhibitors of sialidase from *S. pneumoniae* Nan A, *V. cholerae* and *C. perfringens* using curcumin derivatives. Of the 28 compounds synthesised, 7-(3,4-dihydroxyphenyl)-5-hydroxy-1-(3-hydroxy-4-methoxyphenyl)hepta-1,4,6-trien-3-one (**5e**), which is novel compound contained isoferuloyl and catechol moieties, was the most potent inhibitors of *S. pneumoniae* (IC_50_ = 0.2 ± 0.1 µM) and *C. perfringens* (IC_50_ = 0.5 ± 0.07 µM). All major *Turmeric* components showed noncompetitive inhibition, but compound **5e** exhibited competitive inhibition against *S. pneumoniae* Nan A. In the case of *V. cholerae* sialidase, 7-(4-(dimethylamino)phenyl)-5-hydroxy-1-(4-hydroxy-3-methoxyphenyl)hepta-1,4,6-trien-3-one (**5r**), containing *N*, *N*-dimethylamino as an electron-donating group, was the most potent inhibitor (IC_50_ = 1.9 ± 0.2 µM). The SAR analysis suggested that the *α*, *β*-unsaturated ketone, heptadienyl and either the methoxy or hydroxy group in curcumin was required for sialidase inhibition. These results indicated that curcumin analogues may potentially be used for sepsis caused by bacterial sialidase. Further *in vivo* evaluation of compound **5e** and **5r** will be performed in our laboratory.

## Supplementary Material

Supplemental Material

## References

[1] Singer M, Deutschman CS, Seymour CW, et al. The third international consensus definitions for sepsis and septic shock (Sepsis-3). JAMA 2016;315:801–10. 26903338 10.1001/jama.2016.0287 PMC4968574

[2] Martin GS Sepsis, severe sepsis and septic shock: changes in incidence, pathogens and outcomes. Expert Rev anti Infect Ther 2012;10:701–6. 22734959 10.1586/eri.12.50 PMC3488423

[3] Ispahani P, Slack RCB, Donald FE, et al Twenty year surveillance of invasive pneumococcal disease in Nottingham: serogroups responsible and implications for immunization. Arch Dis Child 2004;89:757–62. 15269078 10.1136/adc.2003.036921 PMC1720039

[4] O'Brien KL, Wolfson LJ, Watt JP, et al. Burden of disease caused by Streptococcus pneumoniae in children younger than 5 years: global estimates. Lancet 2009;374:893–902. 19748398 10.1016/S0140-6736(09)61204-6

[5] Bogaert D, De Groot R, Hermans PW Streptococcus pneumoniae colonisation: the key to pneumococcal disease. Lancet Infect Dis 2004;4:144–54. 14998500 10.1016/S1473-3099(04)00938-7

[6] Mitchell TJ Virulence factors and the pathogenesis of disease caused by *Streptococcus pneumoniae* . Res Microbiol 2000;151:413–9. 10961453 10.1016/s0923-2508(00)00175-3

[7] Yang L, Connaris H, Potter JA, et al Structural characterization of the carbohydrate-binding module of Nan A sialidase: a pneumococcal virulence factor. BMC Struct Biol 2015;15:15. 26289431 10.1186/s12900-015-0042-4 PMC4546082

[8] Paton JC, Andrew PW, Boulnois GJ, et al Molecular analysis of the pathogenicity of Streptococcus pneumoniae: the role of pneumococcal proteins. Annu Rev Microbiol 1993;47:89–115. 7903033 10.1146/annurev.mi.47.100193.000513

[9] Lee Y, Ryu YB, Youn HS, et al. Structural basis of sialidase in complex with geranylated flavonoids as potent natural inhibitors. Acta Cryst 2014;70:1357–65. 10.1107/S1399004714002971 PMC4014123 24816104

[10] Park JY, Lim SH, Kim BR, et al. Sialidase inhibitory activity of diarylnonanoid and neolignan compounds extracted from the seeds of *Myristica fragrans* . Bioorg Med Chem Lett 2017;27:3060–4. 28551100 10.1016/j.bmcl.2017.05.055

[11] Gut H, King SJ, Walsh MA Structural and functional studies of *Streptococcus pneumoniae* neuraminidase B: an intramolecular trans-sialidase. FEBS Lett 2008;582:3348–52. 18775704 10.1016/j.febslet.2008.08.026

[12] Coats MT, Murphy T, Paton JC, et al. Exposure of Thomsen-Friedenreich antigen in Streptococcus pneumoniae infection is dependent on pneumococcal neuraminidase A. Microb Pathog 2011;50:343–9. 21377521 10.1016/j.micpath.2011.02.010 PMC3088309

[13] Walther E, Xu Z, Richter M, et al. Dual acting neuraminidase inhibitors open new opportunities to disrupt the lethal synergism between *Streptococcus pneumoniae* and influenza virus. Front Microbiol 2016;7:357. 27047471 10.3389/fmicb.2016.00357 PMC4800182

[14] Huh J, Ha TKQ, Kang KB, et al. Methylated flavonoid glycosides from pentarhizidium orientale rhizomes and their inhibitory effects on the H1N1 influenza virus. J Nat Prod 2017; 80:2818–24. 28984452 10.1021/acs.jnatprod.7b00677

[15] Bang S, Li W, Ha TKQ, et al. Anti-influenza effect of the major flavonoids from *Salvia plebeia* R.Br. via inhibition of influenza H1N1 virus neuraminidase. Nat Prod Res 2017; 15:1–5. 10.1080/14786419.2017.1326042 28504013

[16] Woo HS, Kim DW, Curtis-Long MJ, et al. Potent inhibition of bacterial neuraminidase activity by pterocarpans isolated from the roots of Lespedeza bicolor. Bioorg Med Chem Lett 2011; 21:6100–3. 21911291 10.1016/j.bmcl.2011.08.046

[17] Ryu YB, Jeong HJ, Yoon SY, et al. Influenza virus neuraminidase inhibitory activity of phlorotannins from the edible brown alga Ecklonia cava. J Agric Food Chem 2011; 59:6467–73. 21585204 10.1021/jf2007248

[18] Hoffmann A, Richter M, von Grafenstein S, et al. Discovery and characterization of diazenylaryl sulfonic acids as inhibitors of viral and bacterial neuraminidases. Front Microbiol 2017;8:205. 28261167 10.3389/fmicb.2017.00205 PMC5309245

[19] Grienke U, Richter M, Walther E, et al. Discovery of prenylated flavonoids with dual activity against influenza virus and Streptococcus pneumoniae. Sci Rep 2016;6:27156. 27257160 10.1038/srep27156 PMC4891693

[20] Uddin Z, Song YH, Curtis-Long MJ, et al. Potent bacterial neuraminidase inhibitors, anthraquinone glucosides from *Polygonum cuspidatum* and their inhibitory mechanism. J Ethnopharmacol 2016;193:283–92. 27553976 10.1016/j.jep.2016.08.026

[21] Nabavi SF, Daglia M, Moghaddam AH, et al. Curcumin and liver disease: from chemistry to medicine. Compr Rev Food Sci Food Saf 2014;13:62–77. 10.1111/1541-4337.12047 33412694

[22] Dulbecco P, Savarino V Therapeutic potential of curcumin in digestive diseases. World J Gastroenterol 2013;19:9256–70. 24409053 10.3748/wjg.v19.i48.9256 PMC3882399

[23] Majhi A, Rahman GM, Panchal S, et al Binding of curcumin and its long chain derivatives to the activator binding domain of novel protein kinase C. Bioorg Med Chem 2010;18:1591–8. 20100661 10.1016/j.bmc.2009.12.075 PMC2843403

[24] Farnsworth NR, Bunyapraphatsara N Thai medicinal plants: recommended for Primary health care system, Bangkok, Medicinal Plant Information Center 1992;409. (ISBN: 9745874981)

[25] Chattopadhyay I, Biswas K, Bandyopadhyay U, et al Turmeric and curcumin: Biological activity and medicinal applications. Curr Sci 2004;87:44–53.

[26] Internet News Turmeric – an effective therapy for Crohn's disease. Nat Prod Rad (CSIR) 2004;3:115

[27] Mishra S, Palanivelu K The effect of curcumin (turmeric) on Alzheimer’s disease: an overview. Ann Indian Acad Neurol 2008;11:13–9. 19966973 10.4103/0972-2327.40220 PMC2781139

[28] Endo H, Nikaido Y, Nakadate M, et al. Structure activity relationship study of curcumin analogues toward the amyloid-beta aggregation inhibitor. Bioorg Med Chem Lett 2014;24:5621–6. 25467149 10.1016/j.bmcl.2014.10.076

[29] Lin L, Shi Q, Nyarko AK, et al. Antitumor agents. 250. Design and synthesis of new curcumin analogues as potential anti-prostate cancer agents. J Med Chem 2006;49:3963–72. 16789753 10.1021/jm051043z PMC2597393

[30] Du ZY, Liu RR, Shao WY, et al. Alpha-glucosidase inhibition of natural curcuminoids and curcumin analogs. Eur J Med Chem 2006;41:213–8. 16387392 10.1016/j.ejmech.2005.10.012

[31] Pavolini T, Gambarin F, Grinzato AM Curcumin and curcuminoids. Ann Chim Rome 1950;40:280–91.

[32] Mohamad H, Lajis NH, Abas F, et al. Antioxidative constituents of Etlingera elatior. J Nat Prod 2005;68:285–8. 15730265 10.1021/np040098l

[33] Caruso F, Pettinari R, Rossi M, et al. The in vitro antitumor activity of arene-ruthenium(II) curcuminoid complexes improves when decreasing curcumin polarity. J Inorg Biochem 2016;162:44–51. 27293144 10.1016/j.jinorgbio.2016.06.002

[34] Sagnou M, Mitsopoulou KP, Koliopoulos G, et al. Evaluation of naturally occurring curcuminoids and related compounds against mosquito larvae. Acta Trop 2012;123:190–5. 22634203 10.1016/j.actatropica.2012.05.006

[35] Feng JY, Liu ZQ Phenolic and enolic hydroxyl groups in curcumin: which plays the major role in scavenging radicals? J Agric Food Chem 2009;57:11041–6. 19736944 10.1021/jf902244g

[36] Nichols CE, Youssef D, Harris RG, et al Microwave-assisted synthesis of curcumin analogs. ARKIVOC 2016;13:64–72.

[37] Lee SL, Huang WJ, Lin WW, et al. Preparation and anti-inflammatory activities of diarylheptanoid and diarylheptylamine analogs. Bioorg Med Chem 2005;13:6175–81. 16084726 10.1016/j.bmc.2005.06.058

[38] Lozada MC, Enríquez RG, Lobato CE, et al. Synthesis and structure of new heterocyclic derivatives of curcumin. Heterocycles 2005;65:49–58.

[39] Pabon HJJ A synthesis of curcumin and related compounds. Recl Trav Chim Pays-Bas 2010;83:379–86.

[40] Youssef D, Nichols CE, Cameron TS, et al. Design, synthesis, and cytostatic activity of novel cyclic curcumin analogues. Bioorg Med Chem Lett 2007;17:5624–9. 17768050 10.1016/j.bmcl.2007.07.079

[41] Fadda AA, Badria FA, El-Attar KM Synthesis and evaluation of curcumin analogues as cytotoxic agents‏. Med Chem Res 2010;19:413–30.

[42] Changtam C, Koning HP, Ibrahim H, et al. Curcuminoid analogs with potent activity against *Trypanosoma* and *Leishmania* species. Eur J Med Chem 2010;45:941–56. 20004045 10.1016/j.ejmech.2009.11.035

