# Supplementary material for: Design, synthesis, and evaluation of curcumin analogues as potential inhibitors of bacterial sialidase
Source: J Enzyme Inhib Med Chem. 2018 Aug 21;33(1):1256–65. doi: 10.1080/14756366.2018.1488695 (PMC6104608; doi:10.1080/14756366.2018.1488695)
Supplement: Supplemental Material [file IENZ_A_1488695_SM0829.pdf]

Electronic Supplementary Information

Design, synthesis, and evaluation of curcumin analogues as potential inhibitors of bacterial sialidase

Bo Ram Kim [a, †], Ji-Young Park [b, †], Hyung Jae Jeong [a], Hyung-Jun Kwon [b], Su-Jin Park [b], In-Chul Lee [b], Young Bae Ryu [b, *], Woo Song Lee [b, *]

Affiliation

[a] *Bio-processing Technology Development and Support Team, Korea Research Institute of Bioscience and Biotechnology, Jeongeup 56212, Republic of Korea*

[b] *Natural Product Material Research Center, Korea Research Institute of Bioscience and Biotechnology, Jeongeup 56212, Republic of Korea*

* Corresponding authors at: Natural Product Material Research Center, Korea Research Institute of Bioscience and Biotechnology, Jeongeup 580-185, Republic of Korea. Tel: +82 63 570 5170; Fax: +82 63 570 5239(W.S. Lee), Tel: +82 63 570 5171 (Y.B. Ryu). E-mail address: wslee@kribb.re.kr (W.S. Lee) and ybryu@kribb.re.kr (Y.B. Ryu).

[†]Both authors contributed equally to the work.

Contents of Supplementary Information

1. **Characterization data**
   - ^{1}H and ^{13}C NMR spectrum of curcumins (pp. S3 – S29)

2. **Preparation of enzyme**
   - Sequence data of prepared enzymes (pp. S30-S31)
   - Michaelis-Menten plots and Linewever-Burk plot of *S. pneumoniae* NanA K_m values (pp. S32)

3. Bioassay plots
   - Lineweaver-Burk and Dixon plots of the compound **4a**, **4e**, **5q** and **5e** (pp. S33 – S34)

1. ^{1}H and ^{13}C NMR spectrum of curcumins

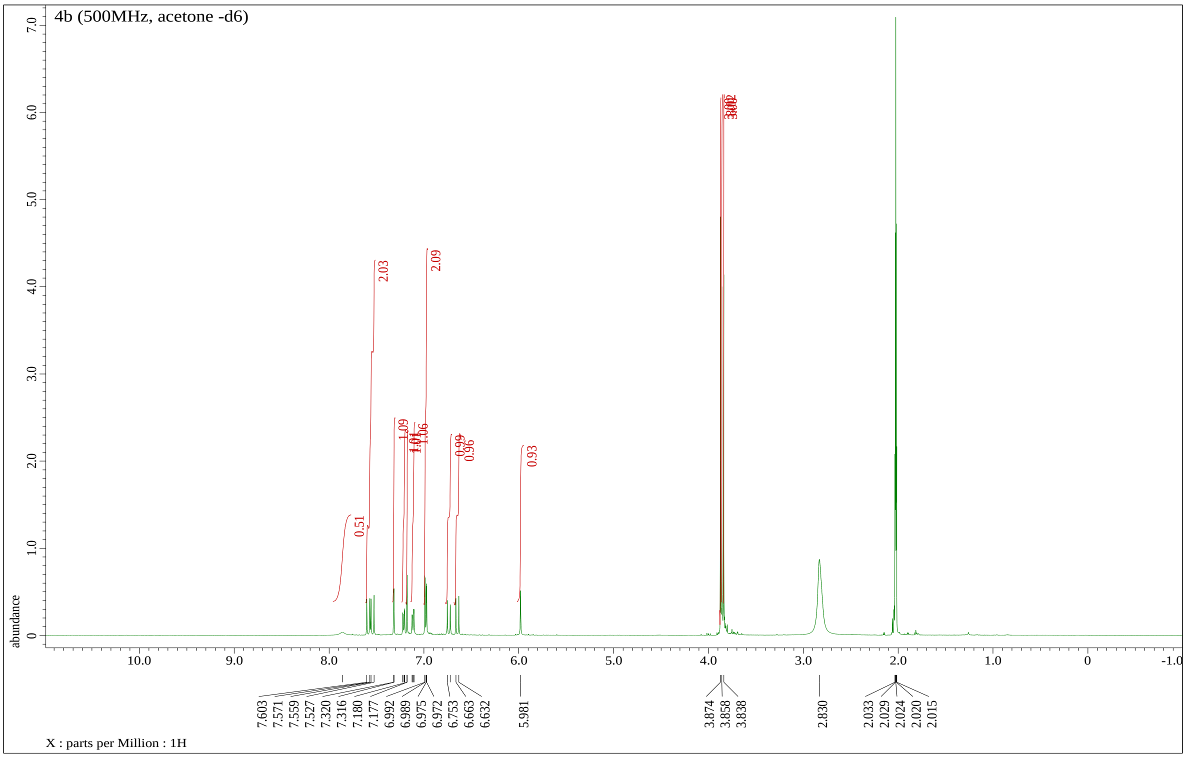

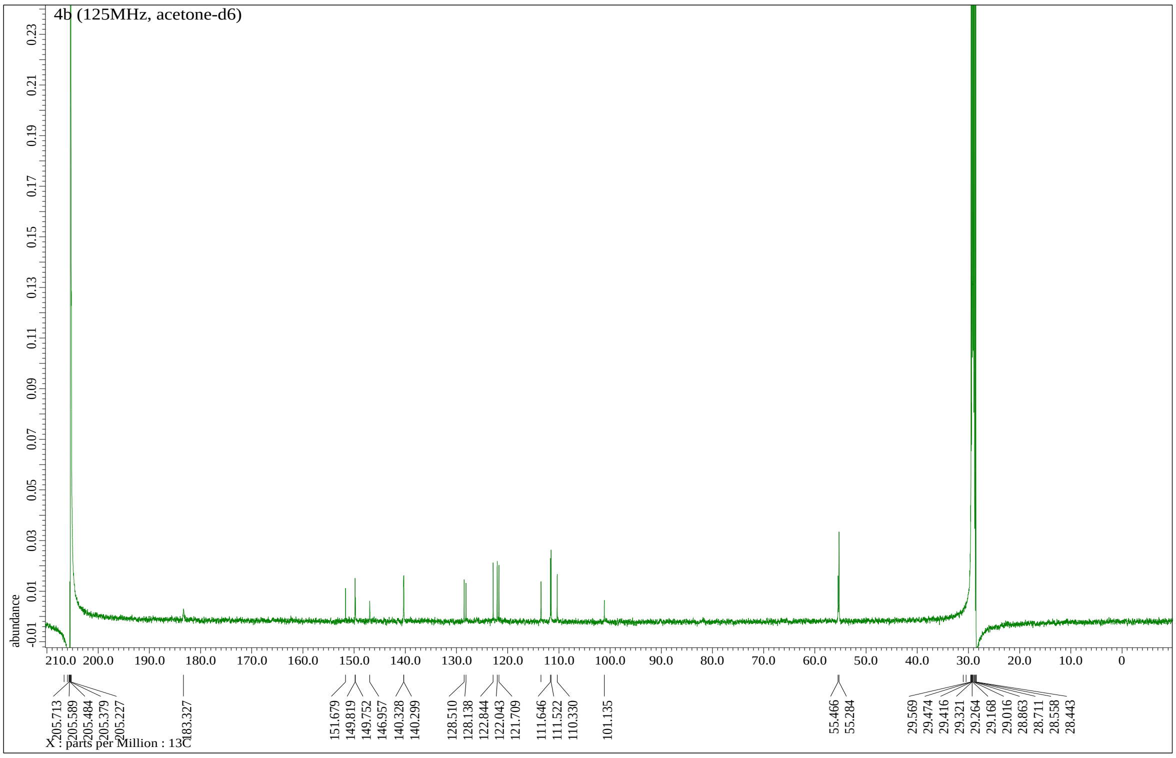

Figure S1. ^{1}H (top) and ^{13}C NMR (bottom) spectra in acetone-d_6 of **4b**

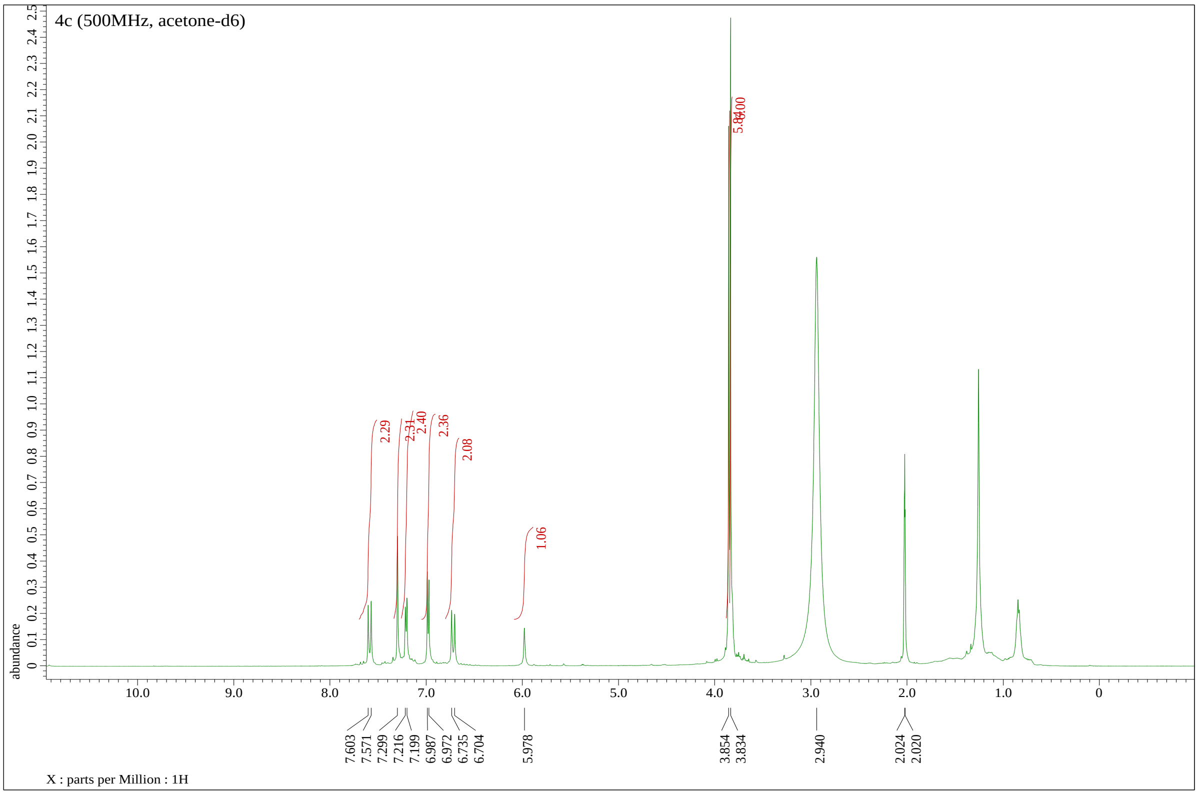

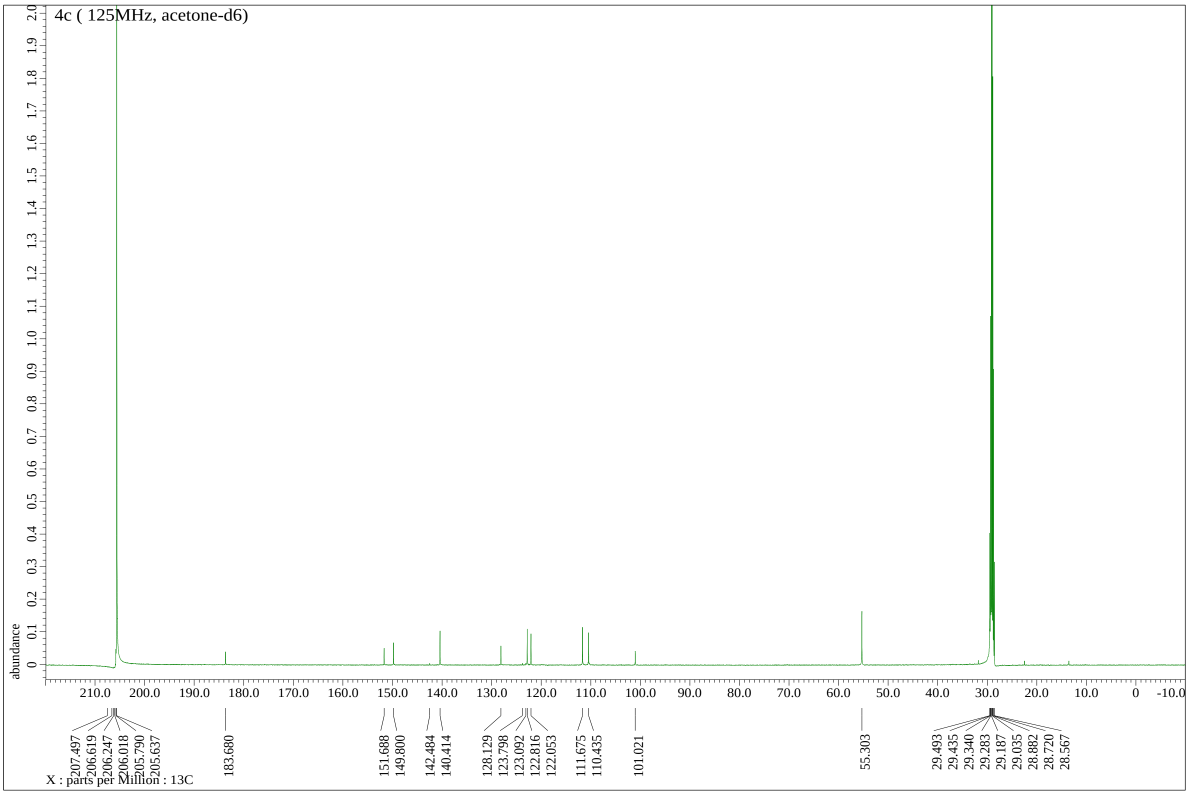

Figure S2. ^{1}H (top) and ^{13}C NMR (bottom) spectra in acetone-d_6 of **4c**

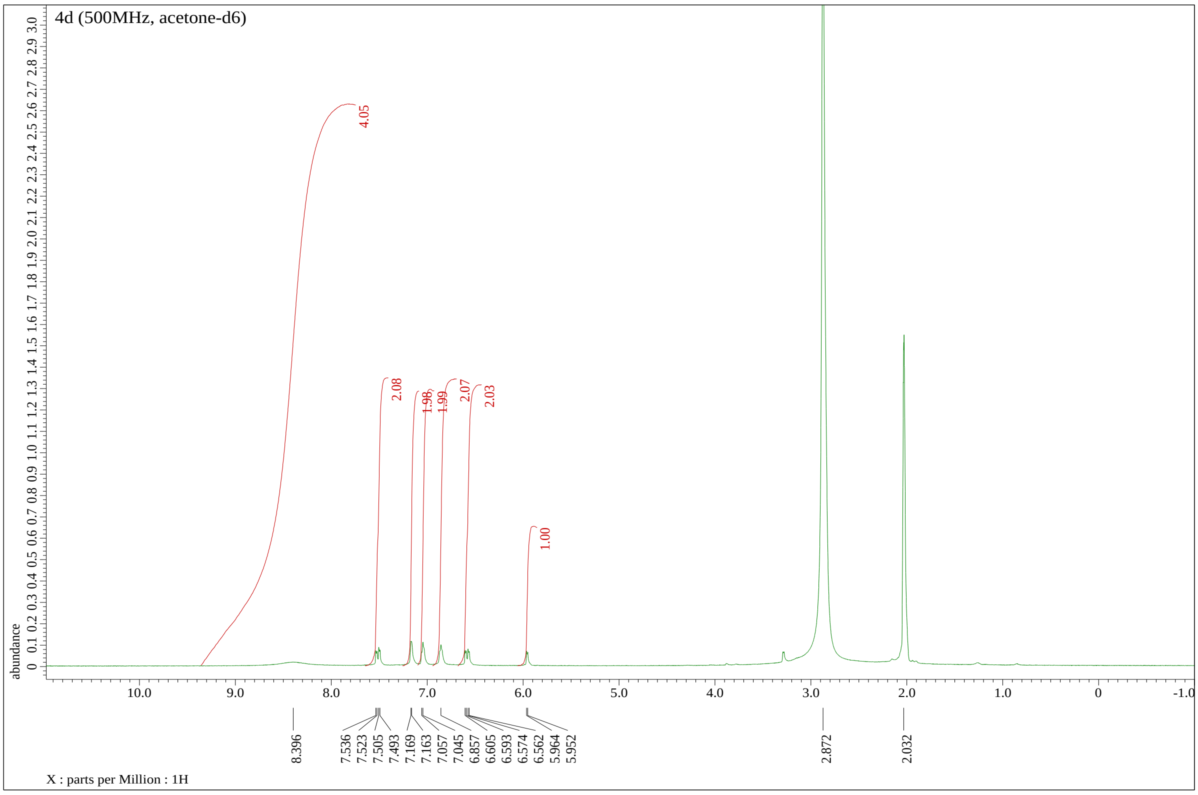

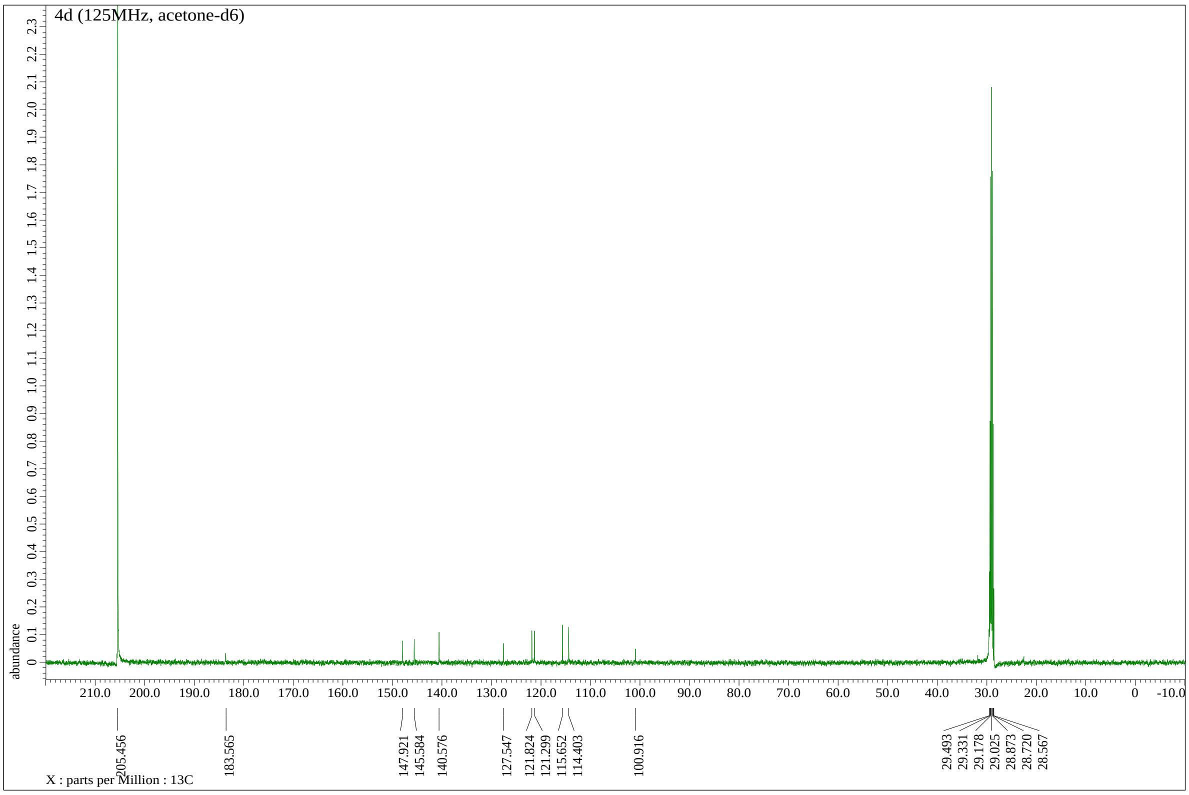

Figure S3. ^{1}H (top) and ^{13}C NMR (bottom) spectra in acetone-d_6 of **4d**

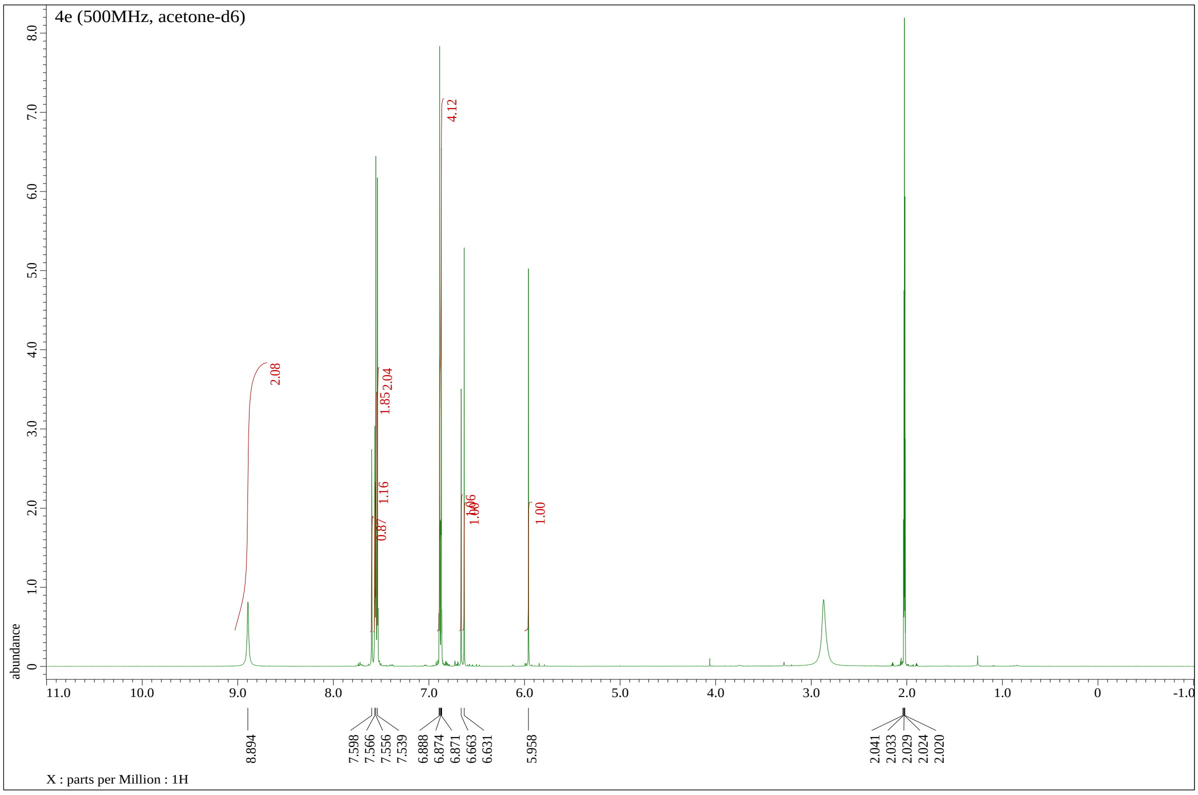

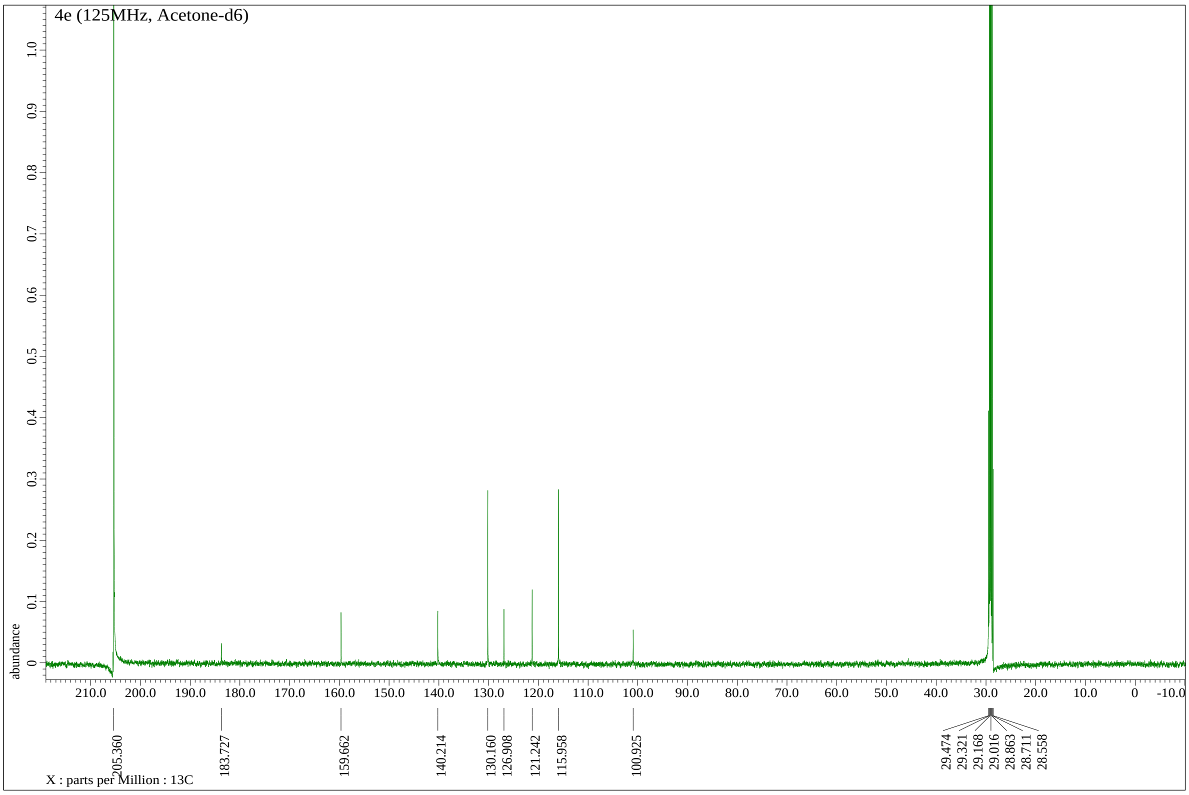

Figure S4. ^{1}H (top) and ^{13}C NMR (bottom) spectra in acetone-d_6 of **4e**

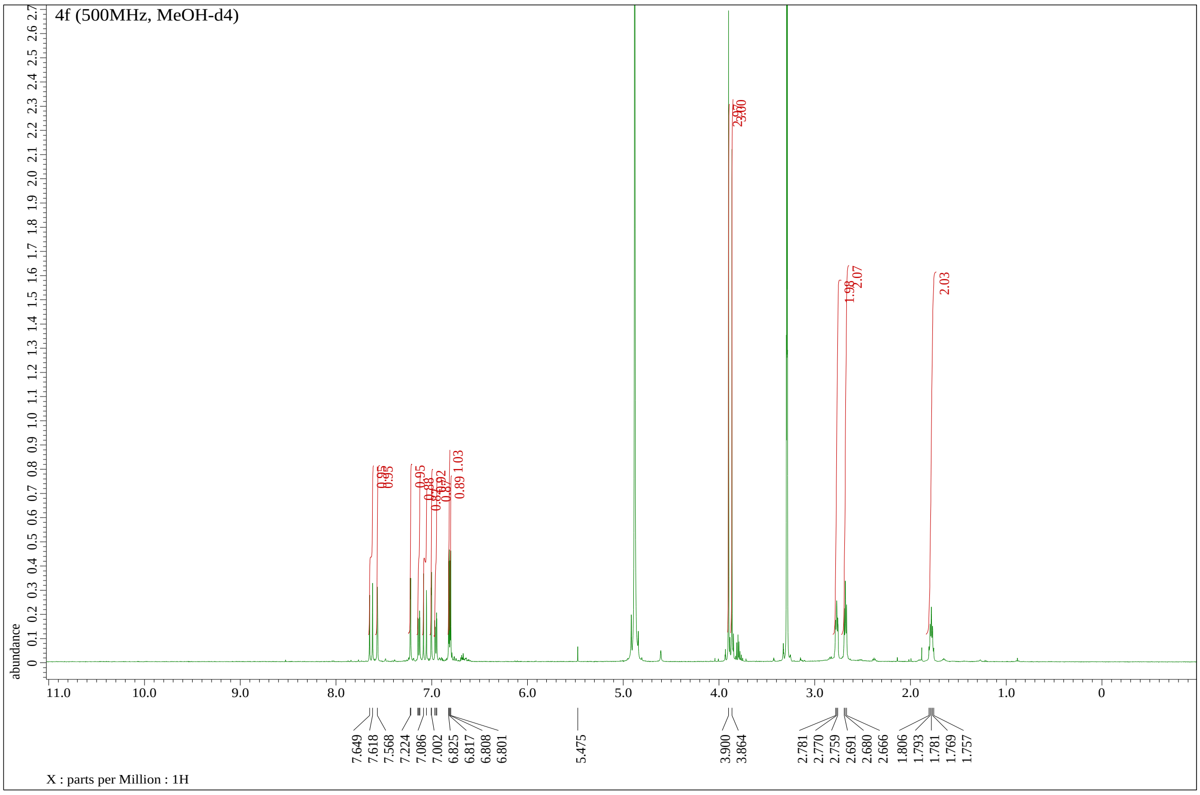

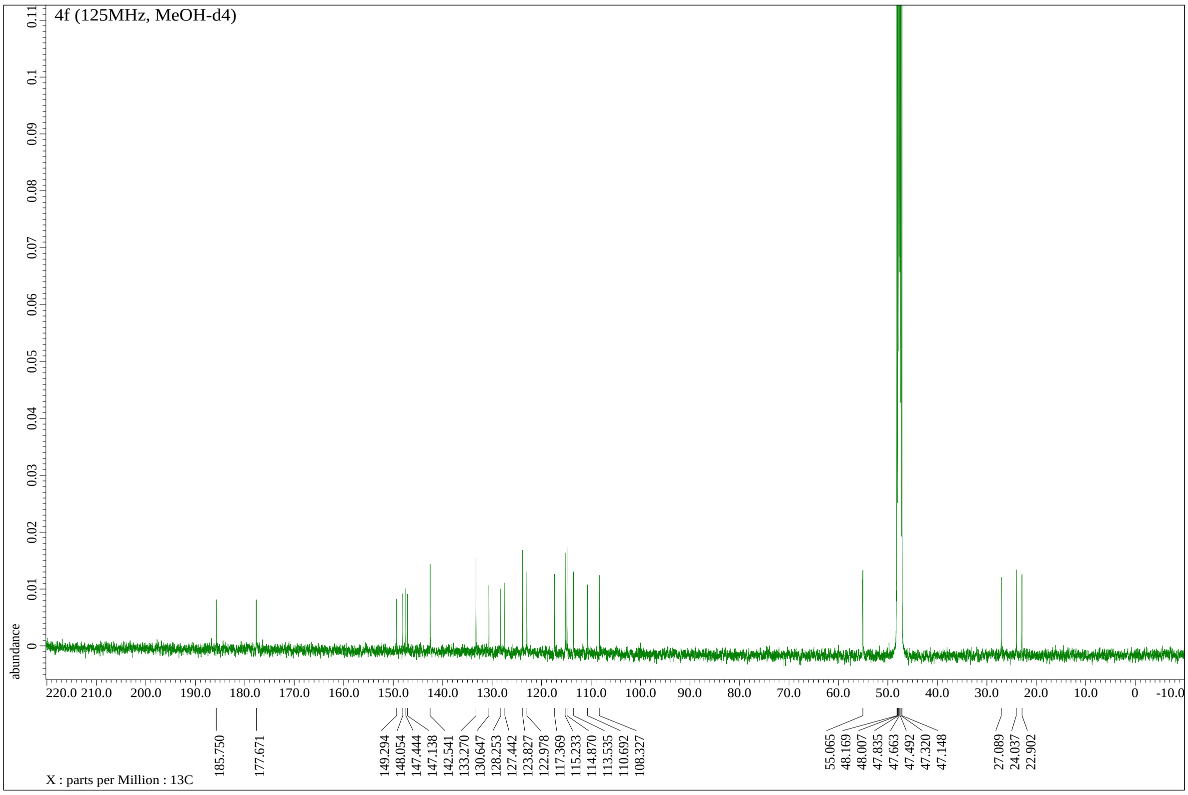

Figure S5. ^{1}H (top) and ^{13}C NMR (bottom) spectra in acetone-d_6 of **4f**

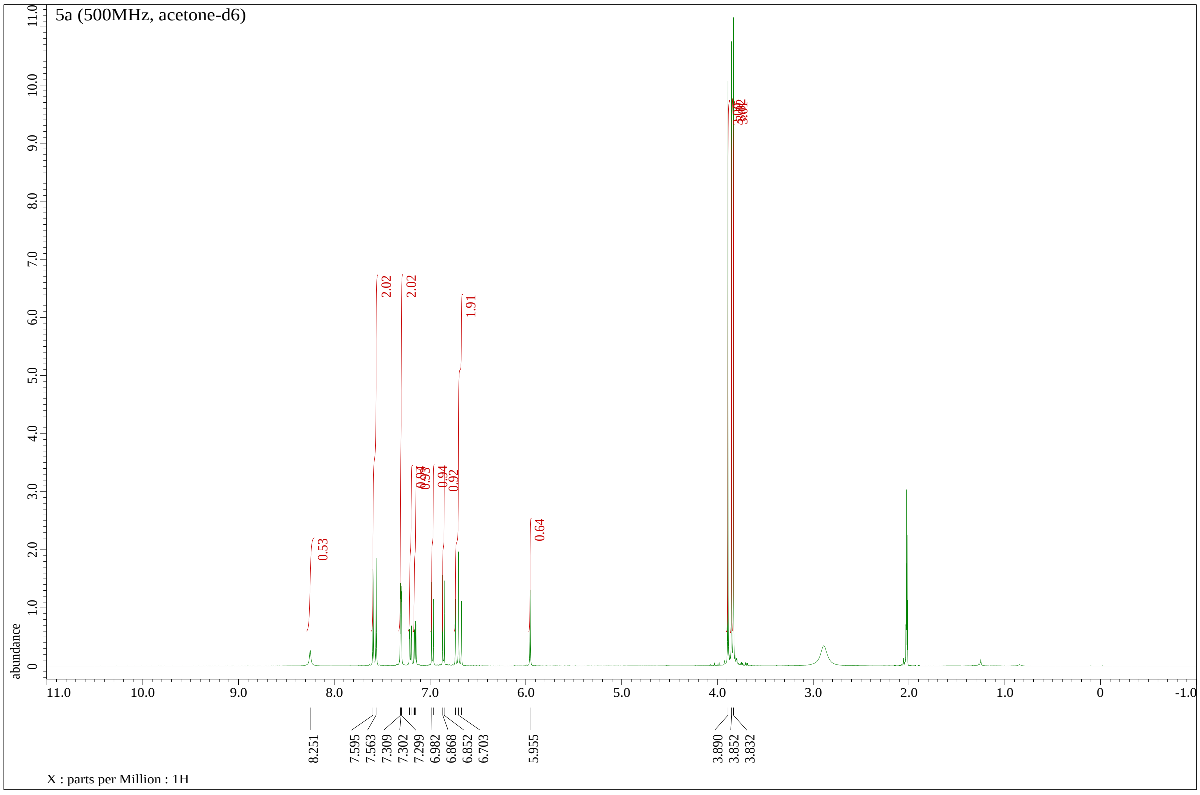

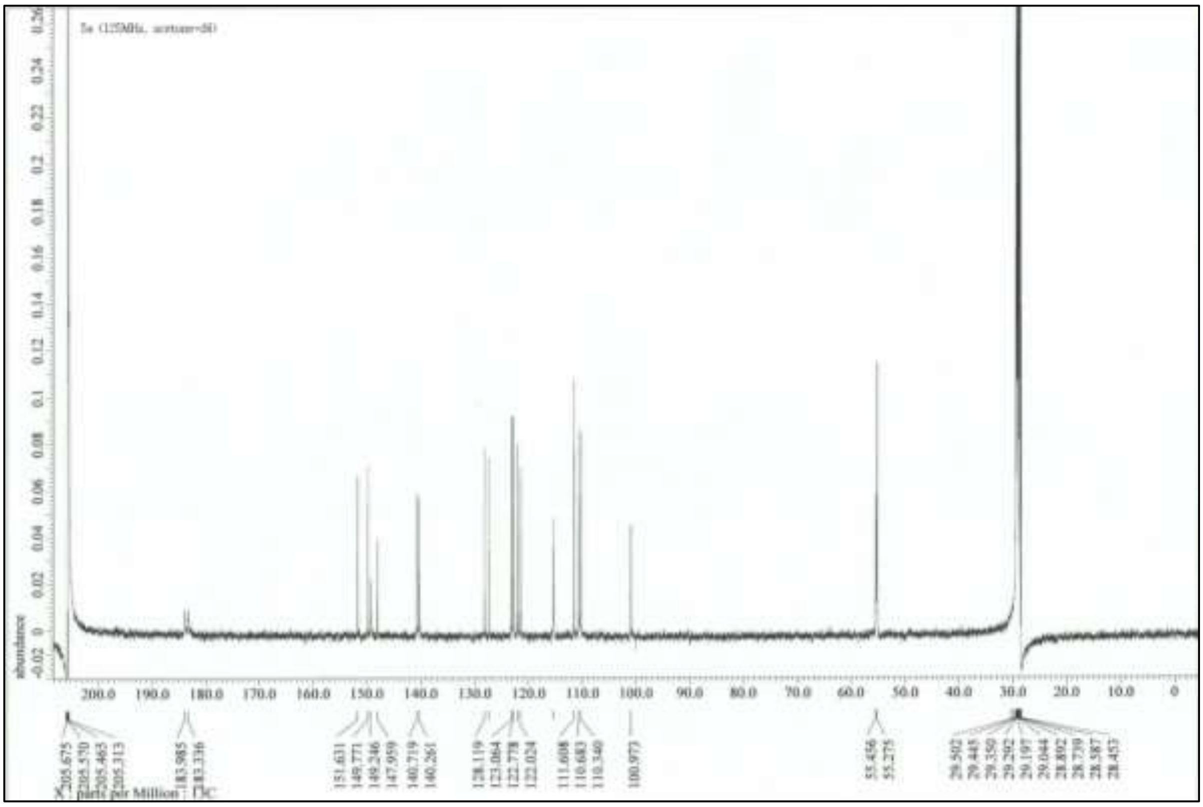

Figure S6. ^{1}H (top) and ^{13}C NMR (bottom) spectra in acetone-d_6 of **5a**

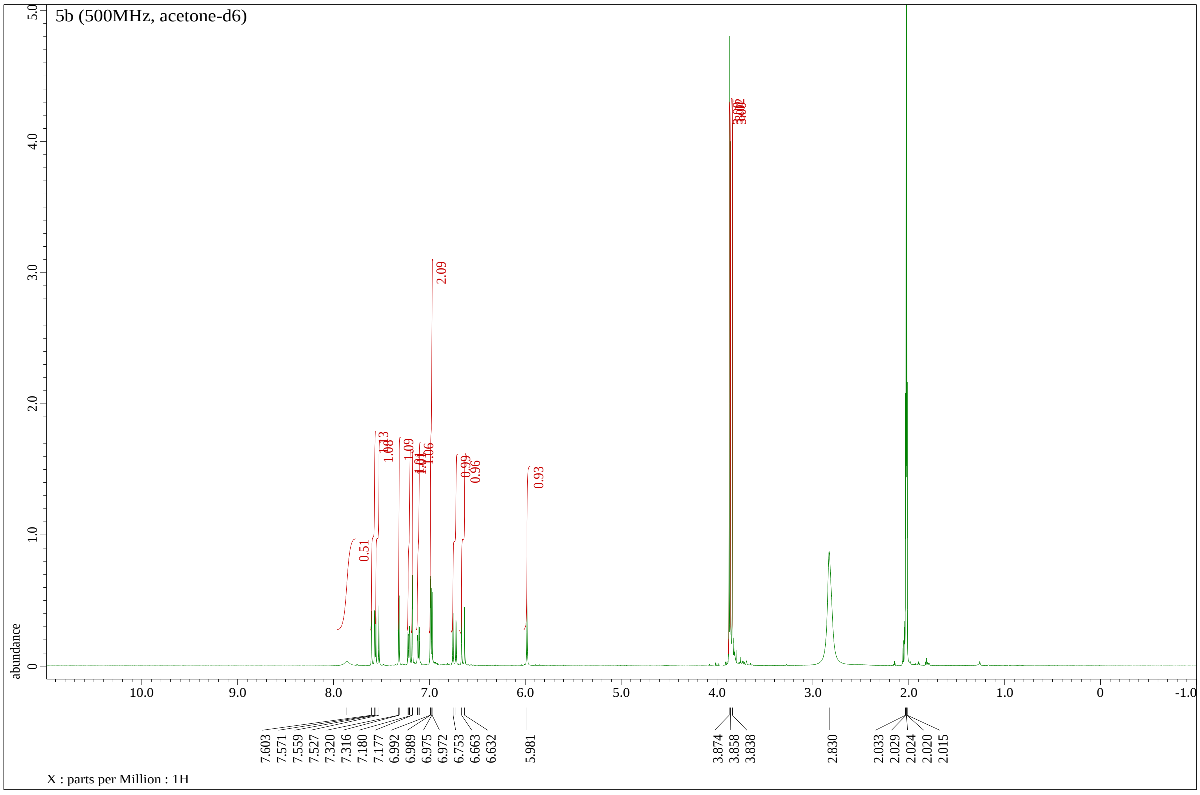

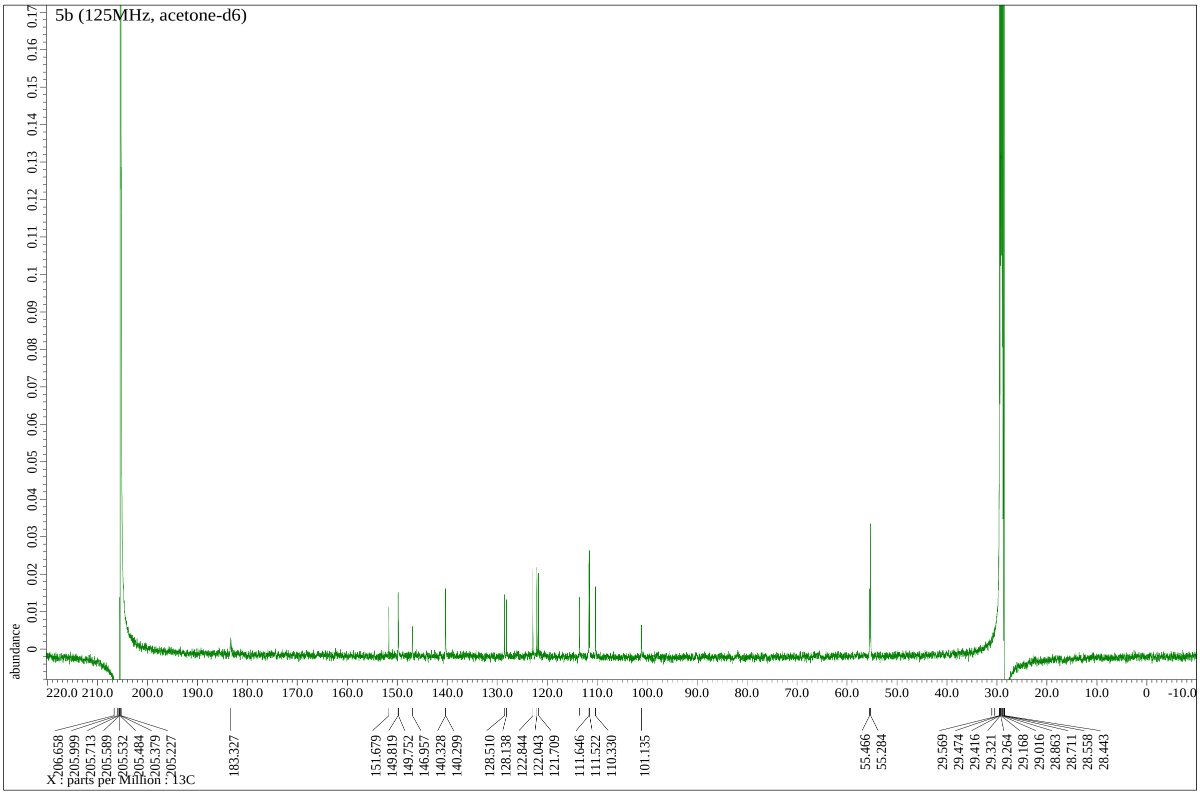

Figure S7. ^{1}H (top) and ^{13}C NMR (bottom) spectra in acetone-d_6 of **5b**

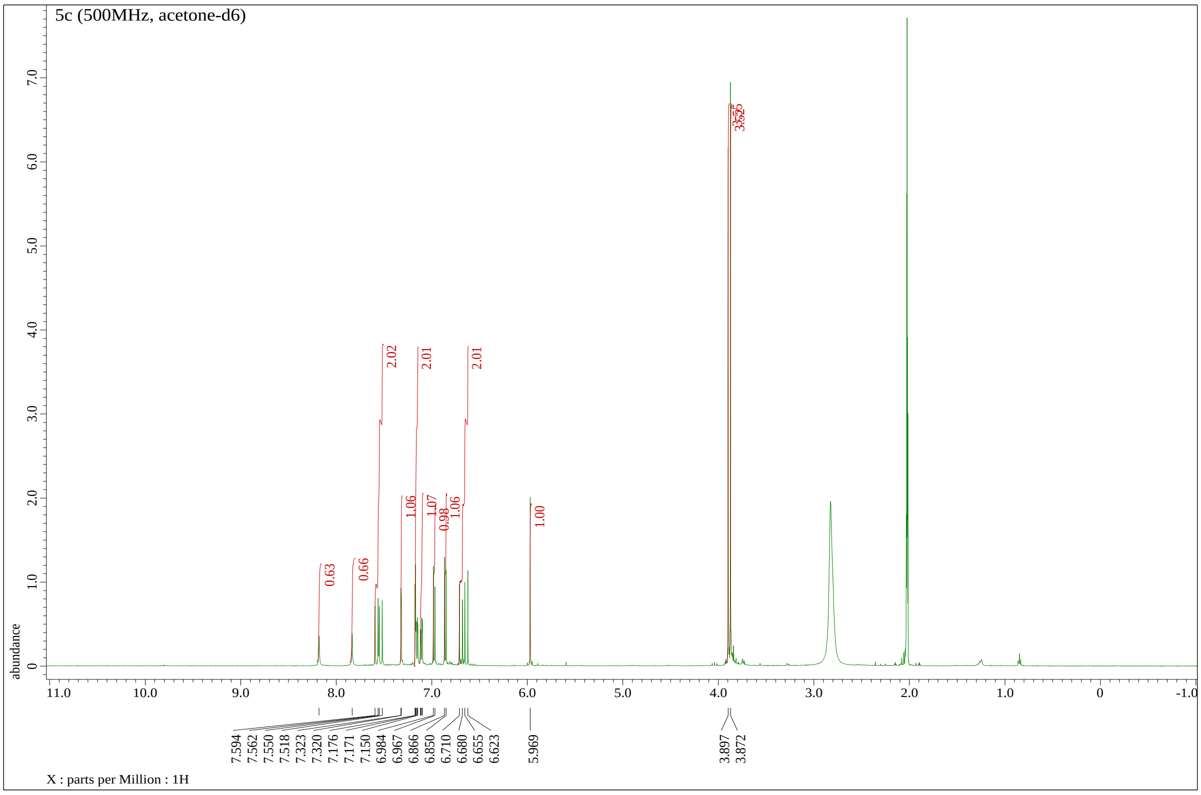

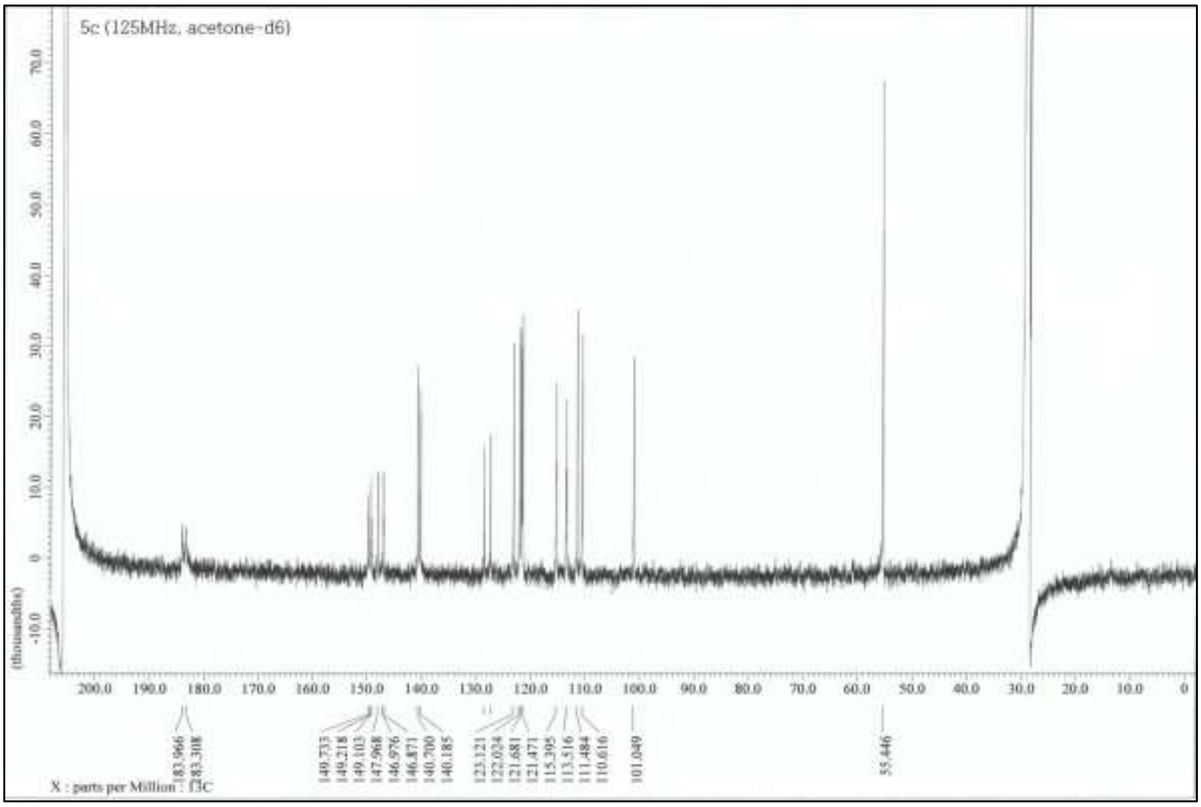

Figure S8. ^{1}H (top) and ^{13}C NMR (bottom) spectra in acetone-d_6 of **5c**

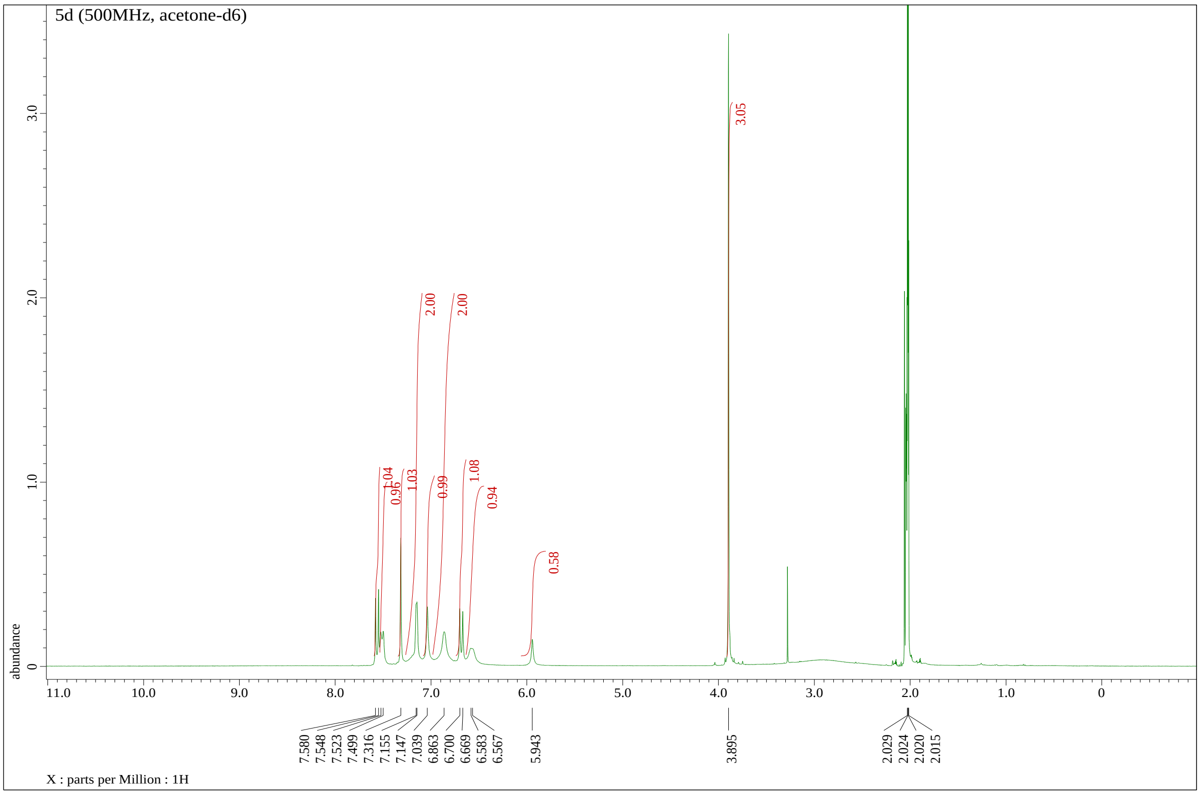

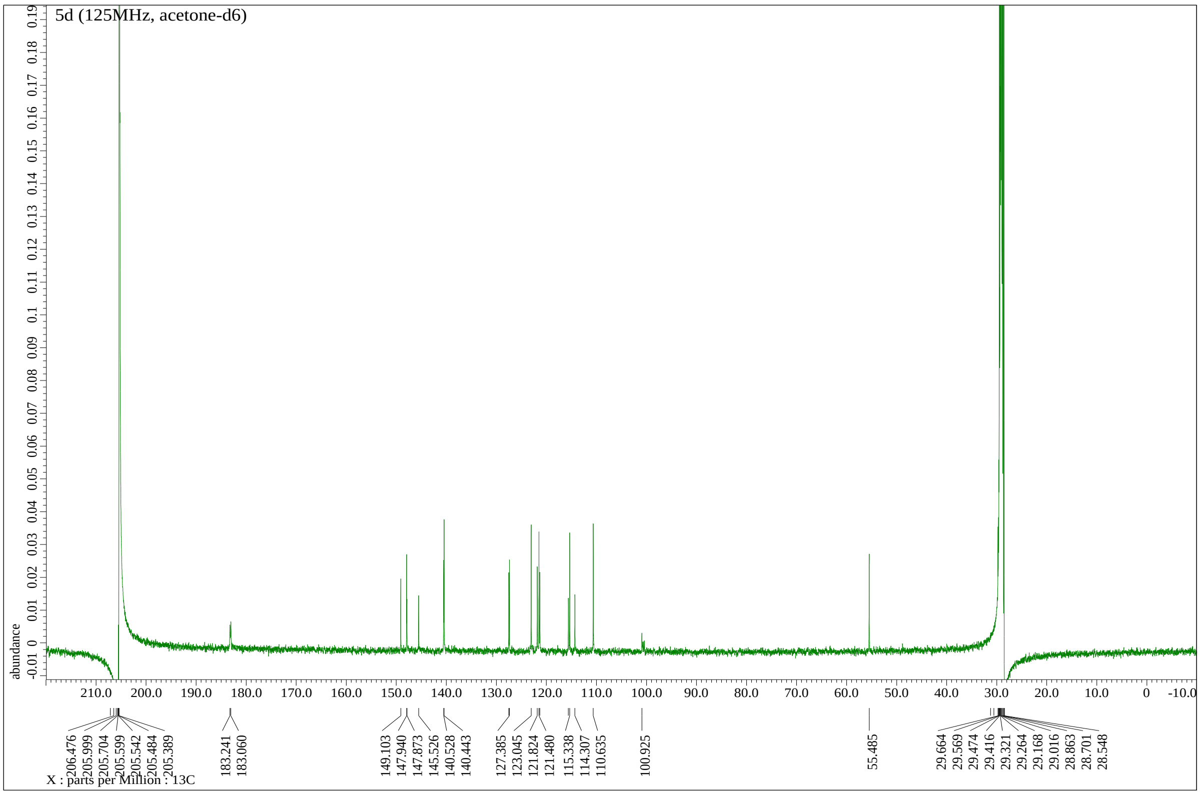

Figure S9. ^{1}H (top) and ^{13}C NMR (bottom) spectra in acetone-d_6 of **5d**

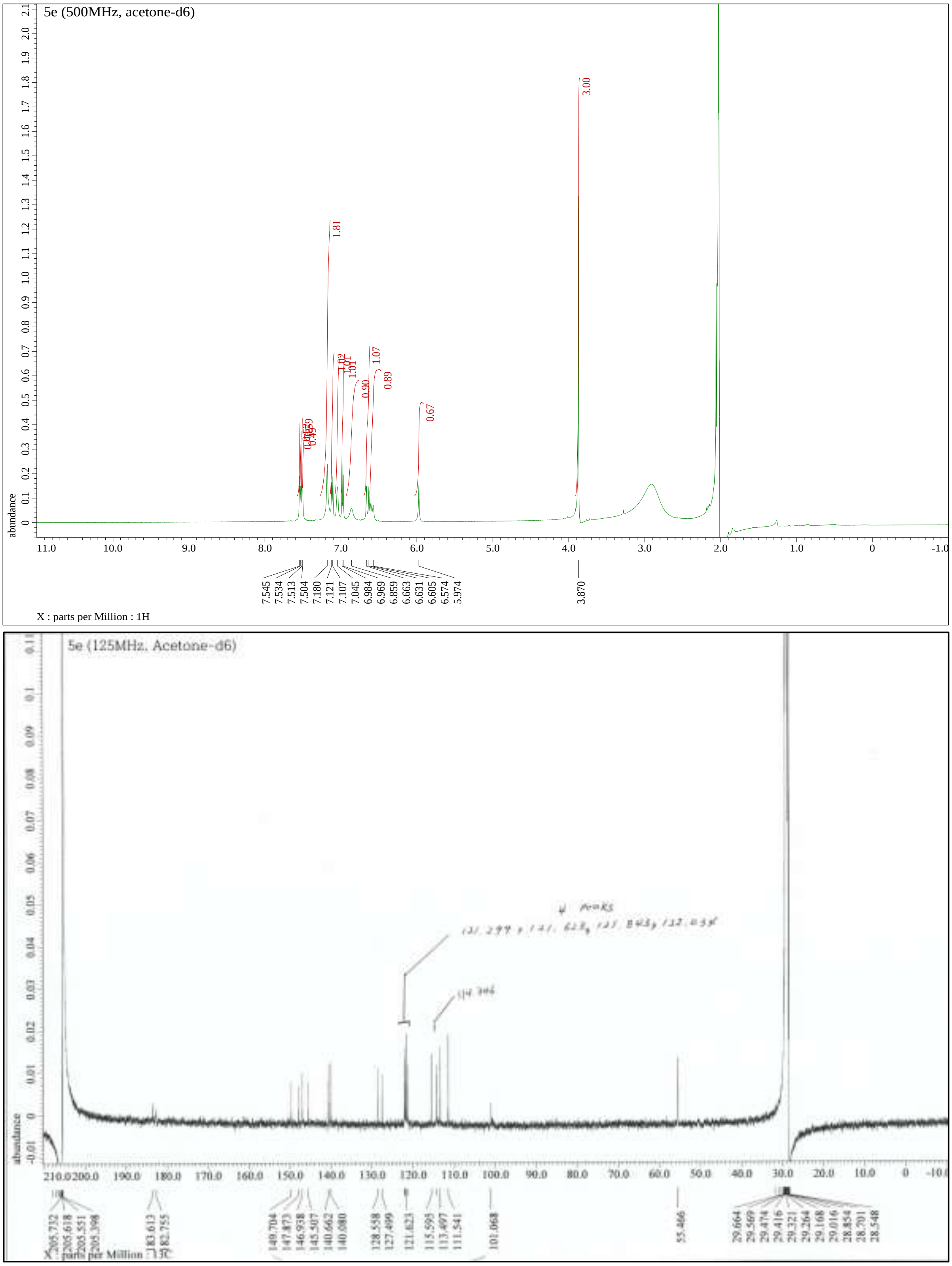

Figure S10. ^{1}H (top) and ^{13}C NMR (bottom) spectra in acetone-d_6 of **5e**

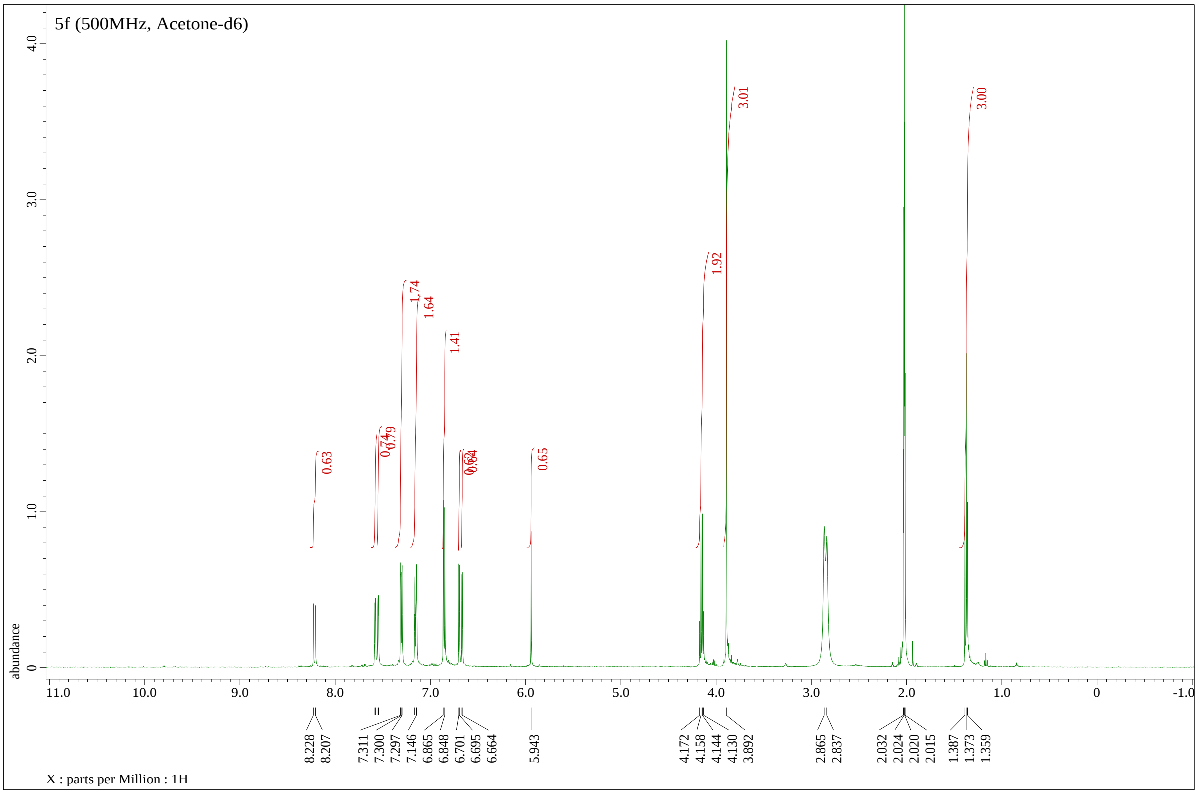

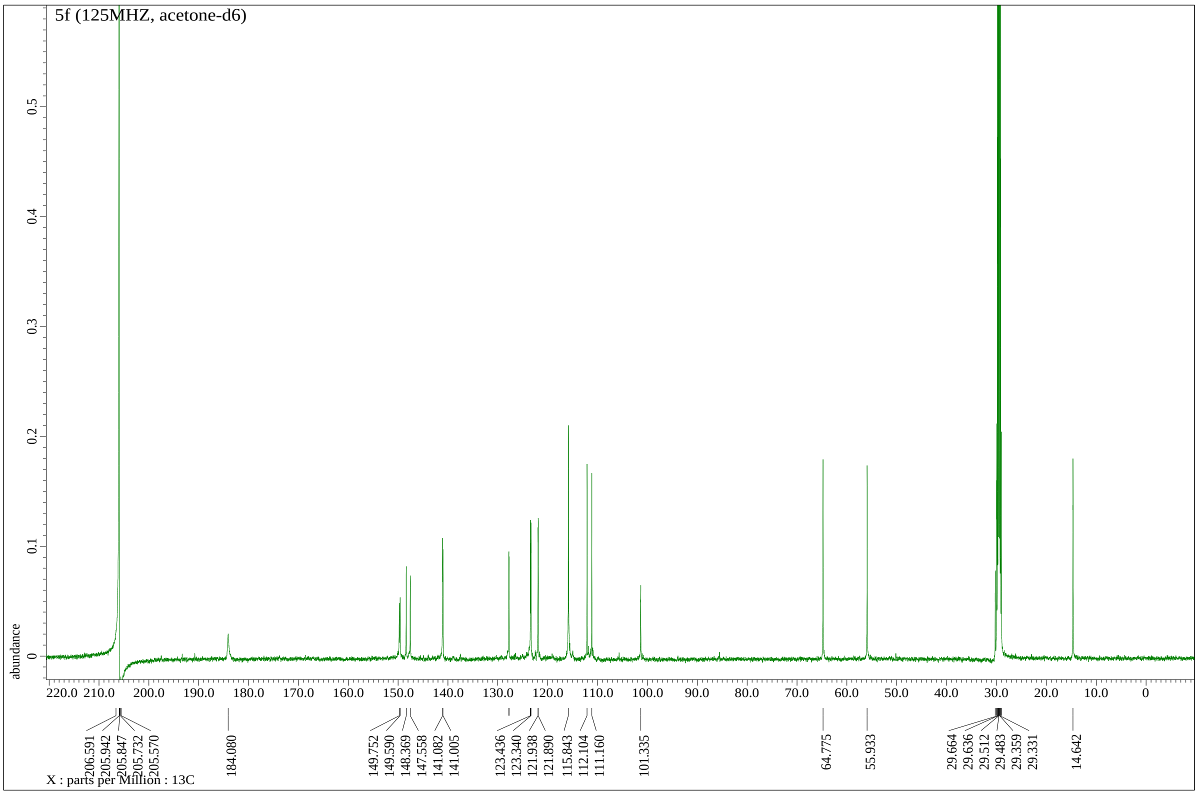

Figure S11. ^{1}H (top) and ^{13}C NMR (bottom) spectra in acetone-d_6 of **5f**

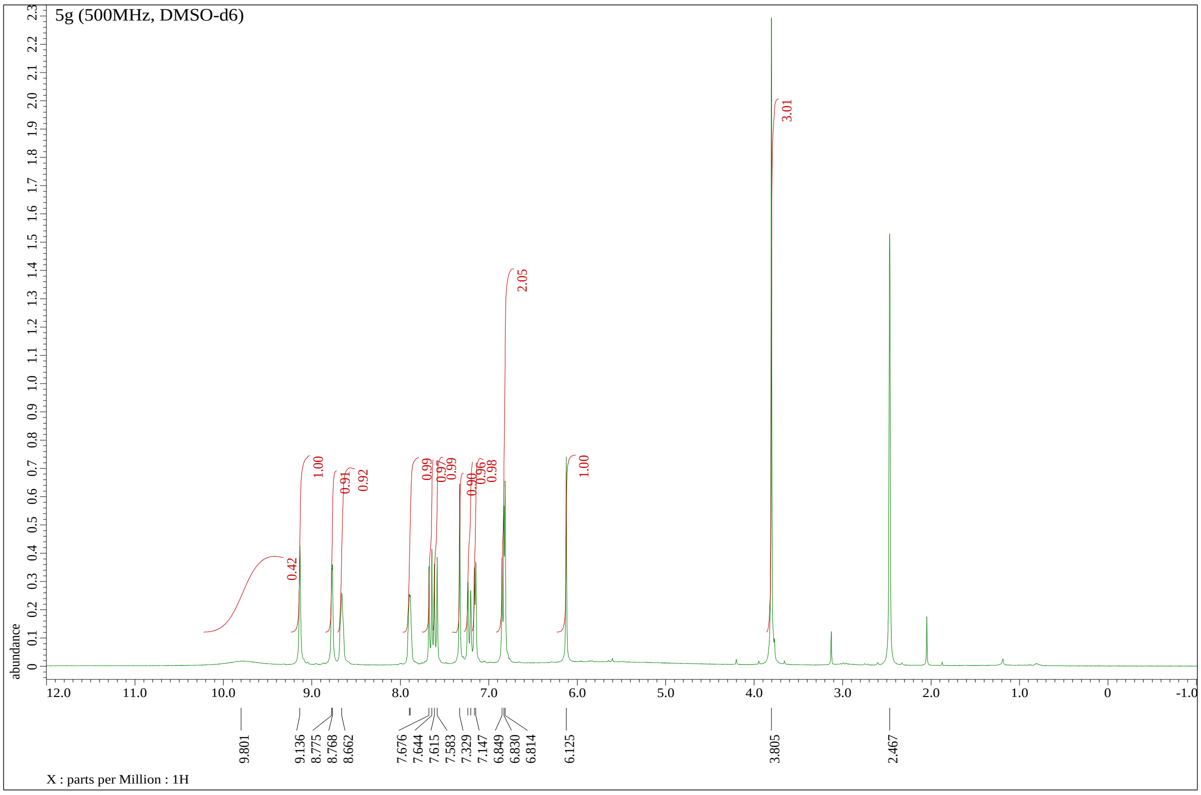

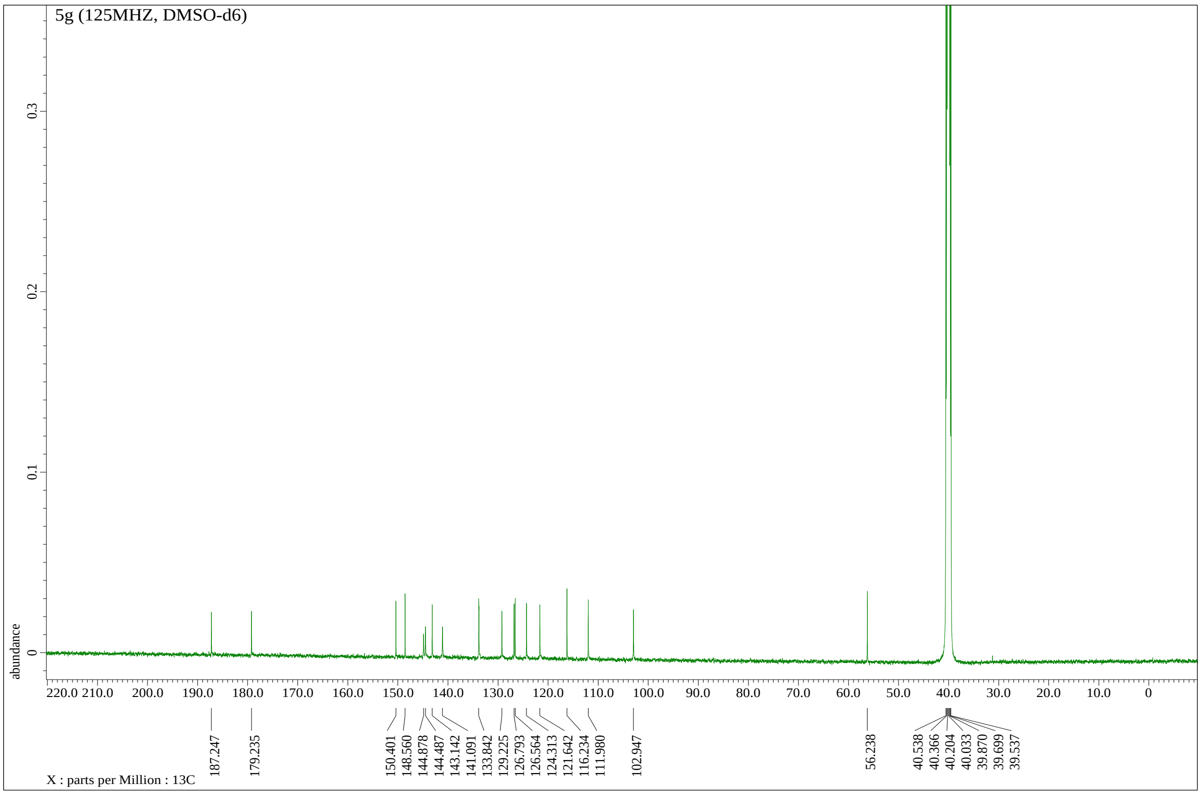

Figure S12. ^{1}H (top) and ^{13}C NMR (bottom) spectra in dimethylsulfoxide-d_6 of **5g**

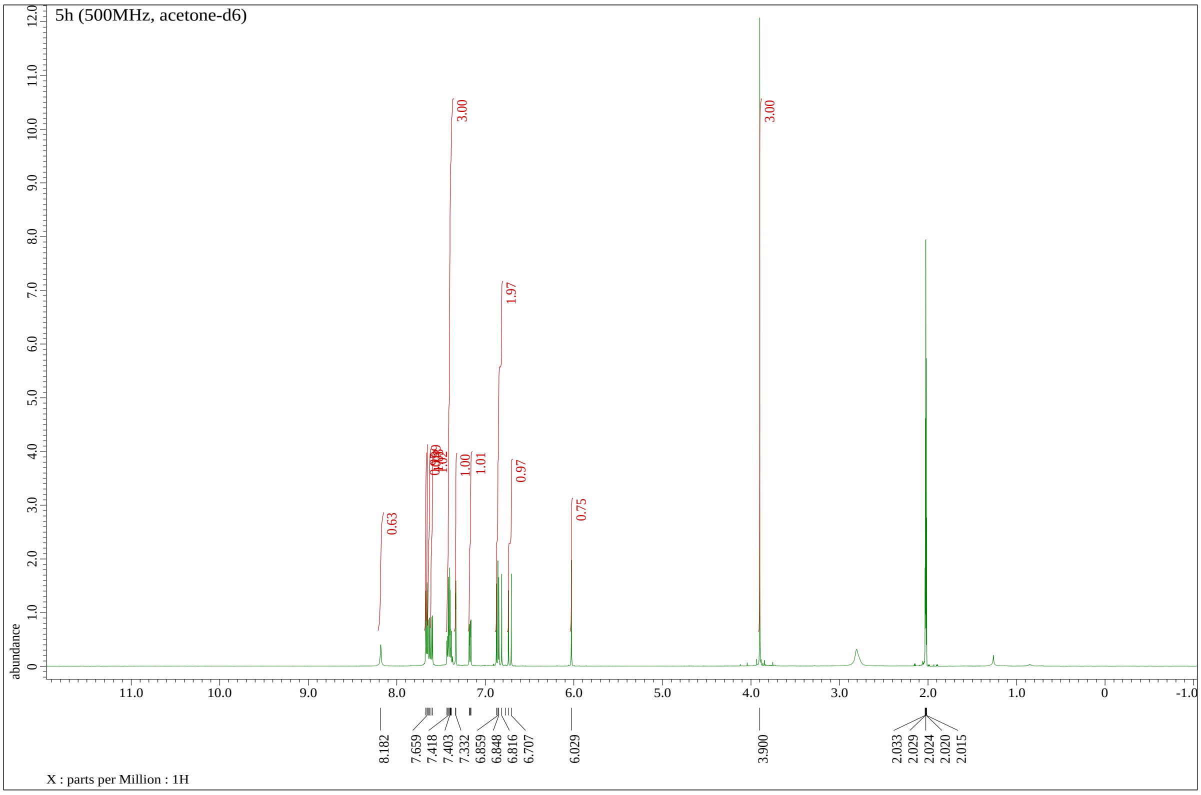

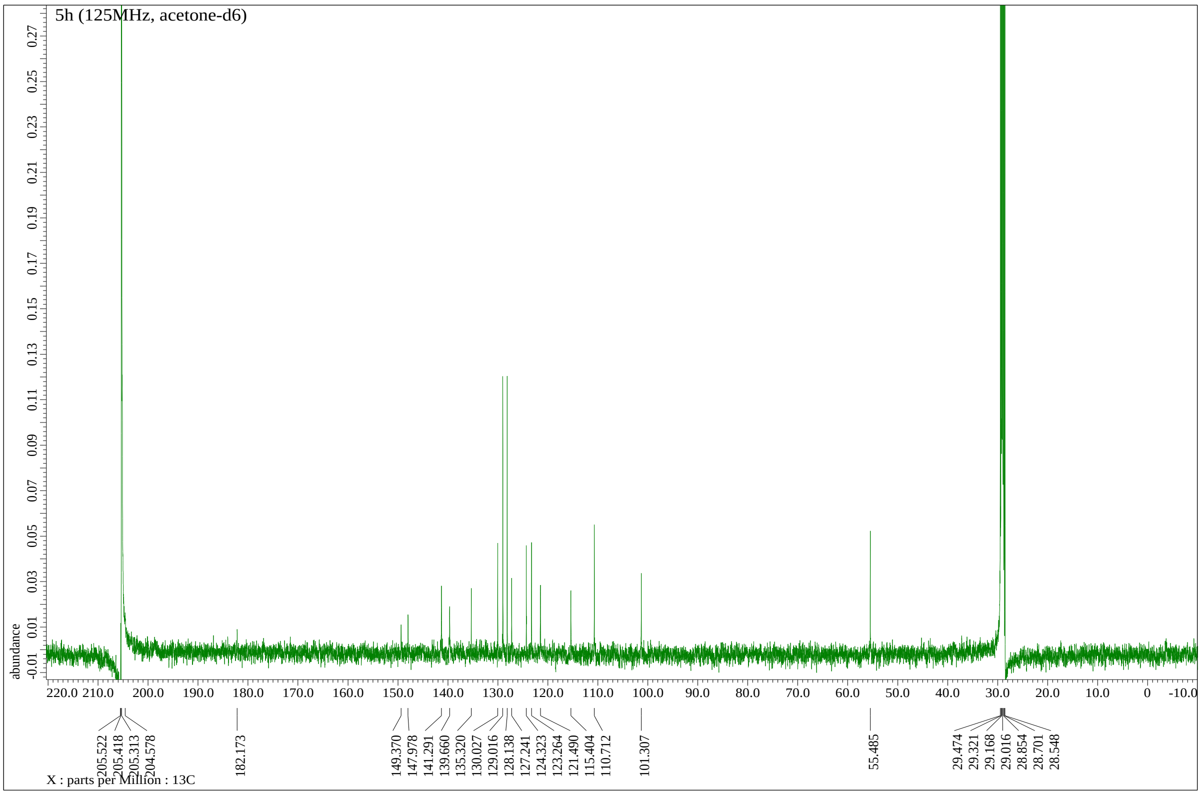

Figure S13. ^{1}H (top) and ^{13}C NMR (bottom) spectra in acetone-d_6 of **5h**

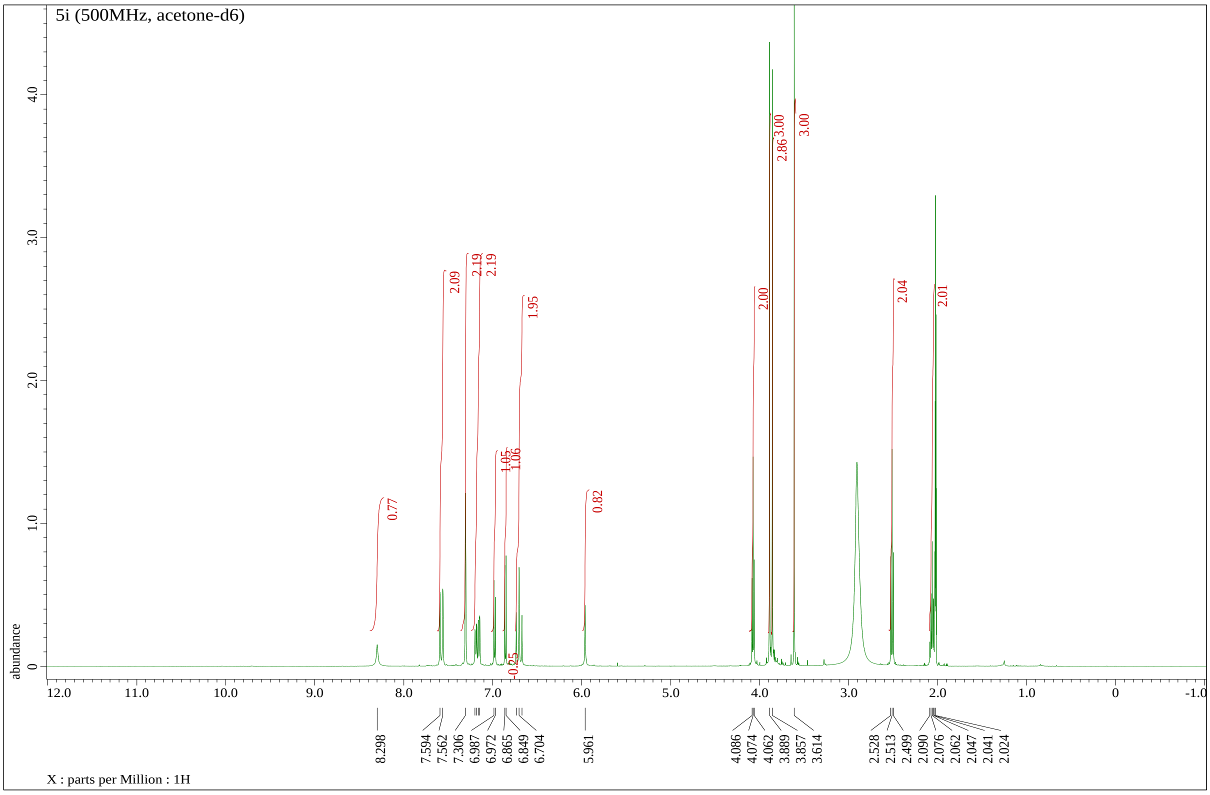

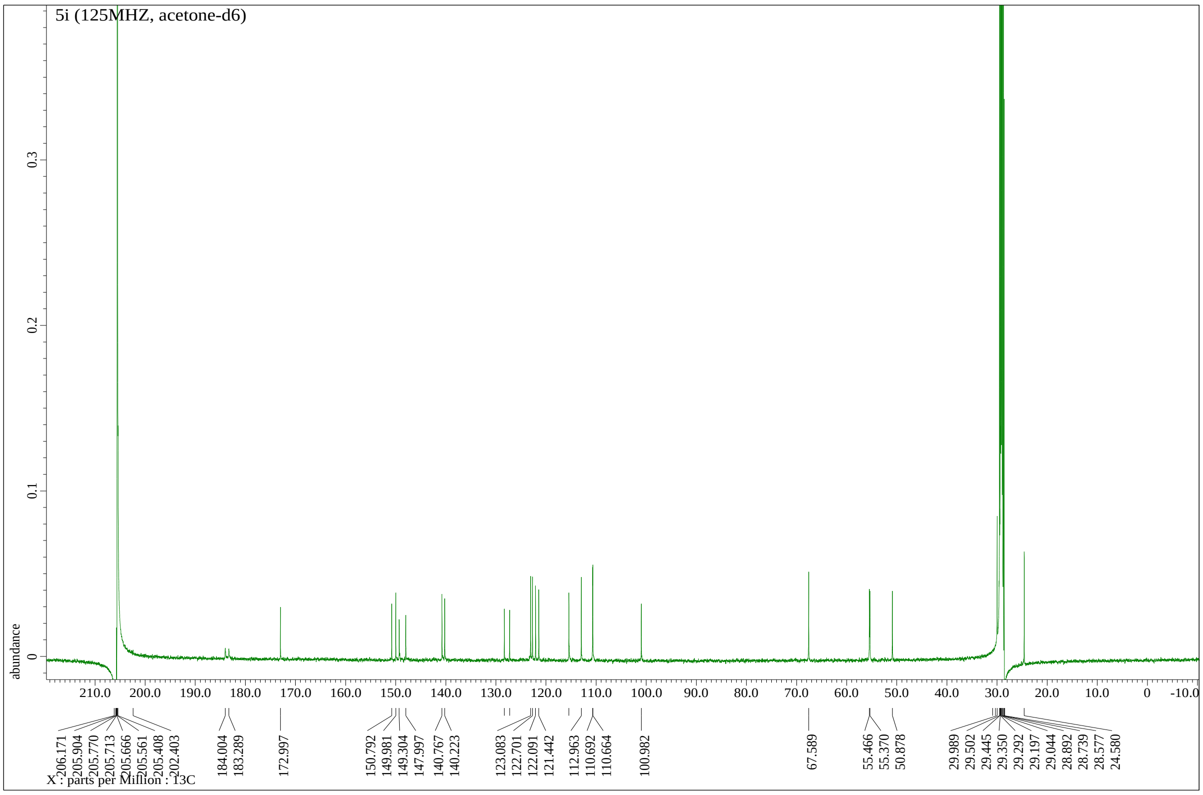

Figure S14. ^{1}H (top) and ^{13}C NMR (bottom) spectra in acetone-d_6 of **5i**

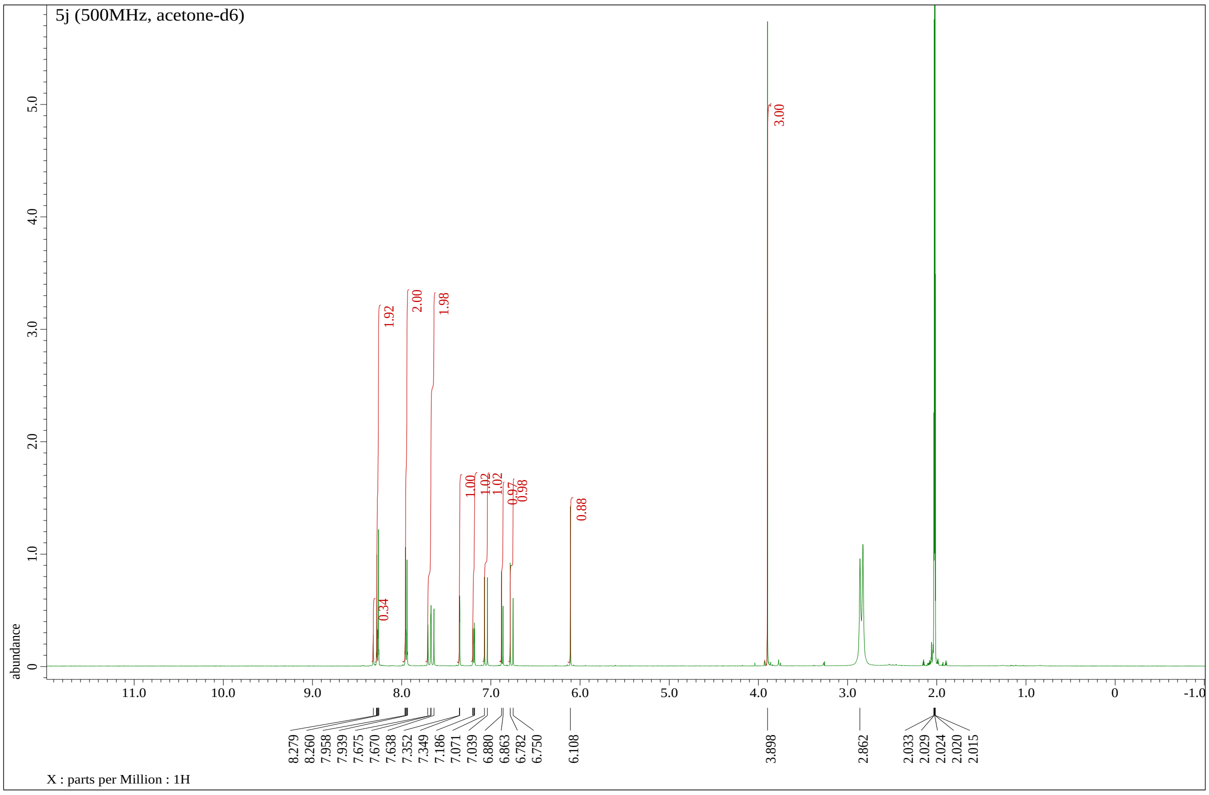

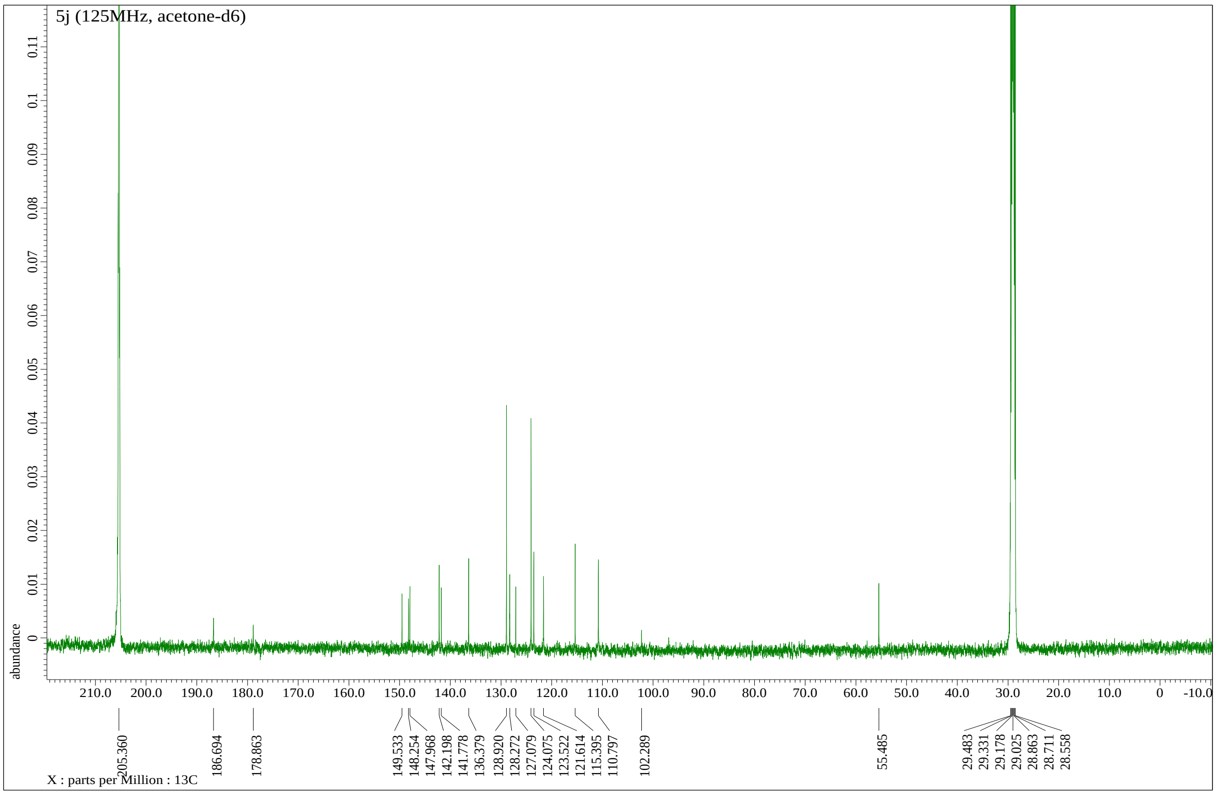

Figure S15. ^{1}H (top) and ^{13}C NMR (bottom) spectra in acetone-d_6 of **5j**

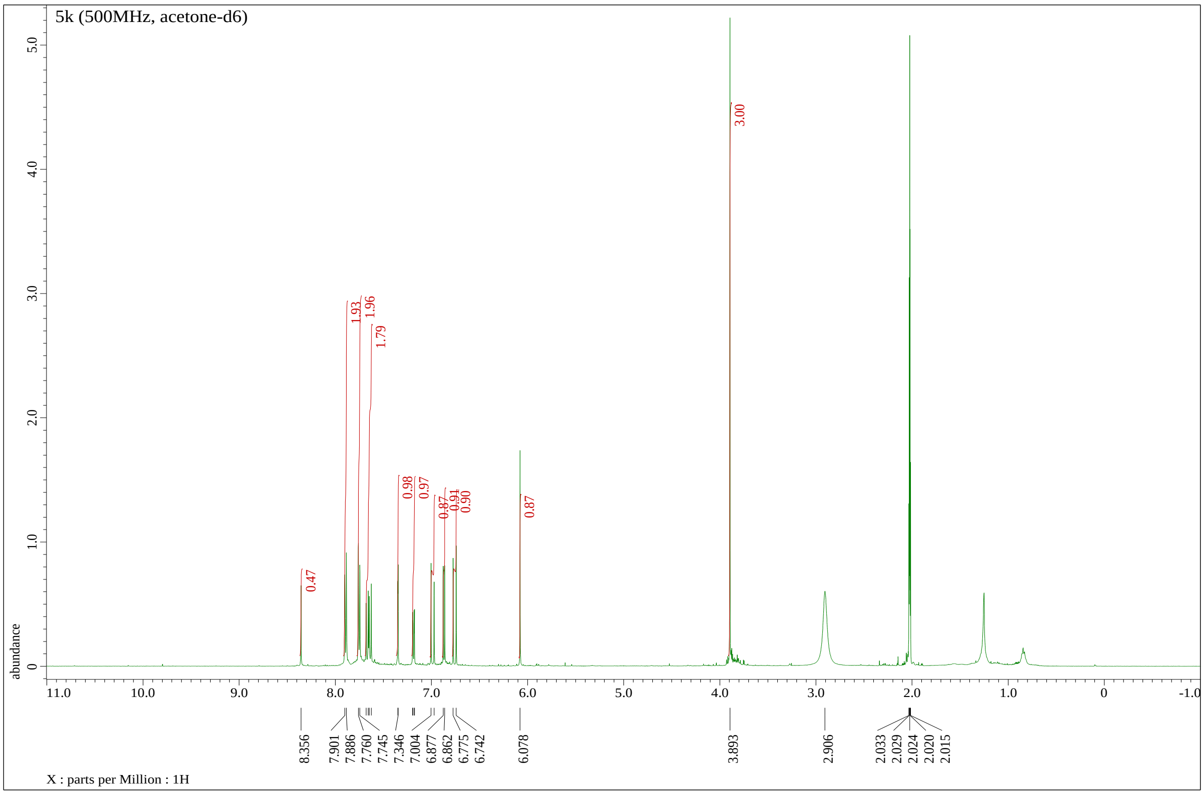

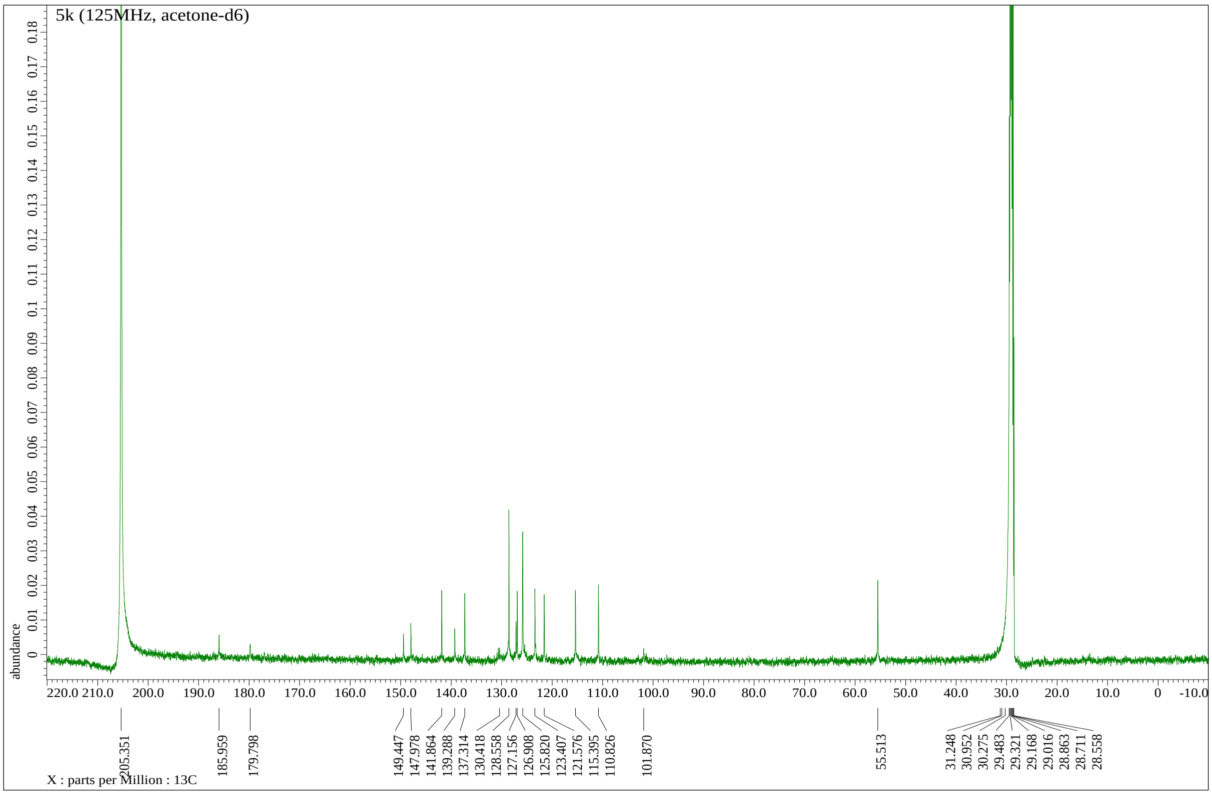

Figure S16. ^{1}H (top) and ^{13}C NMR (bottom) spectra in acetone-d_6 of **5k**

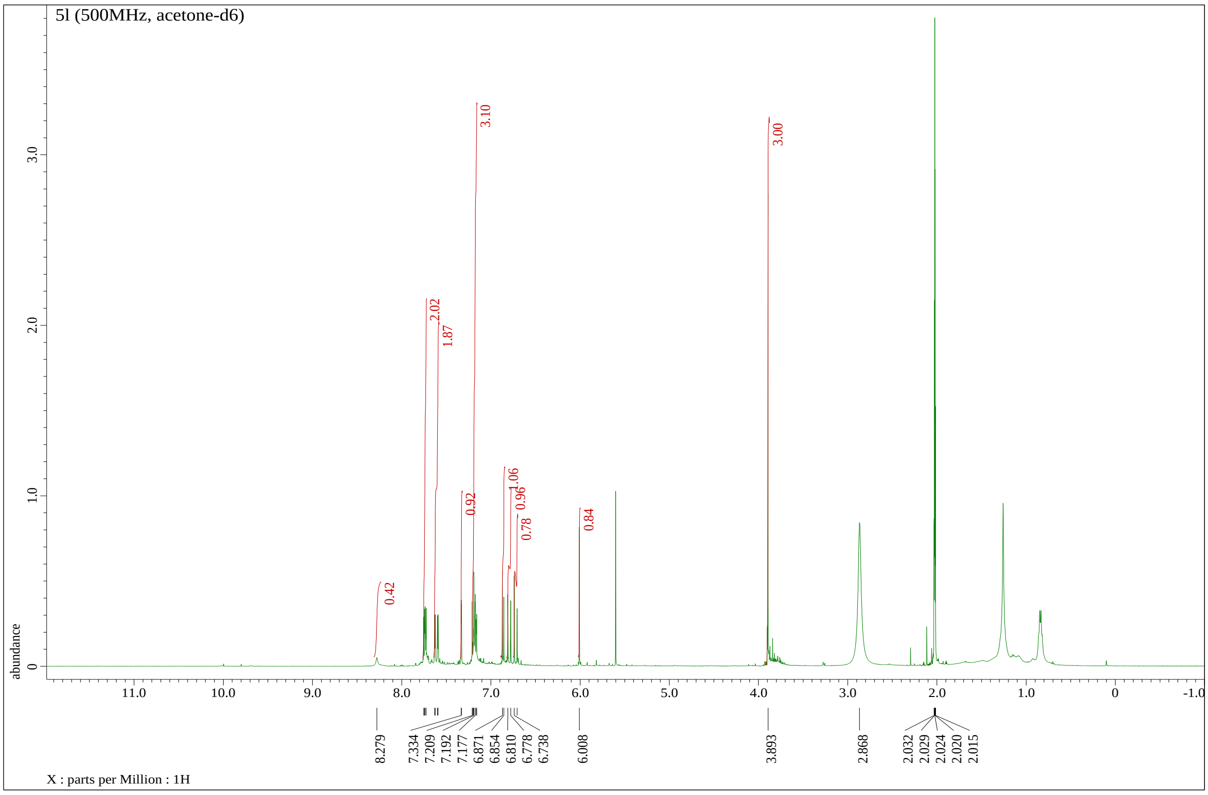

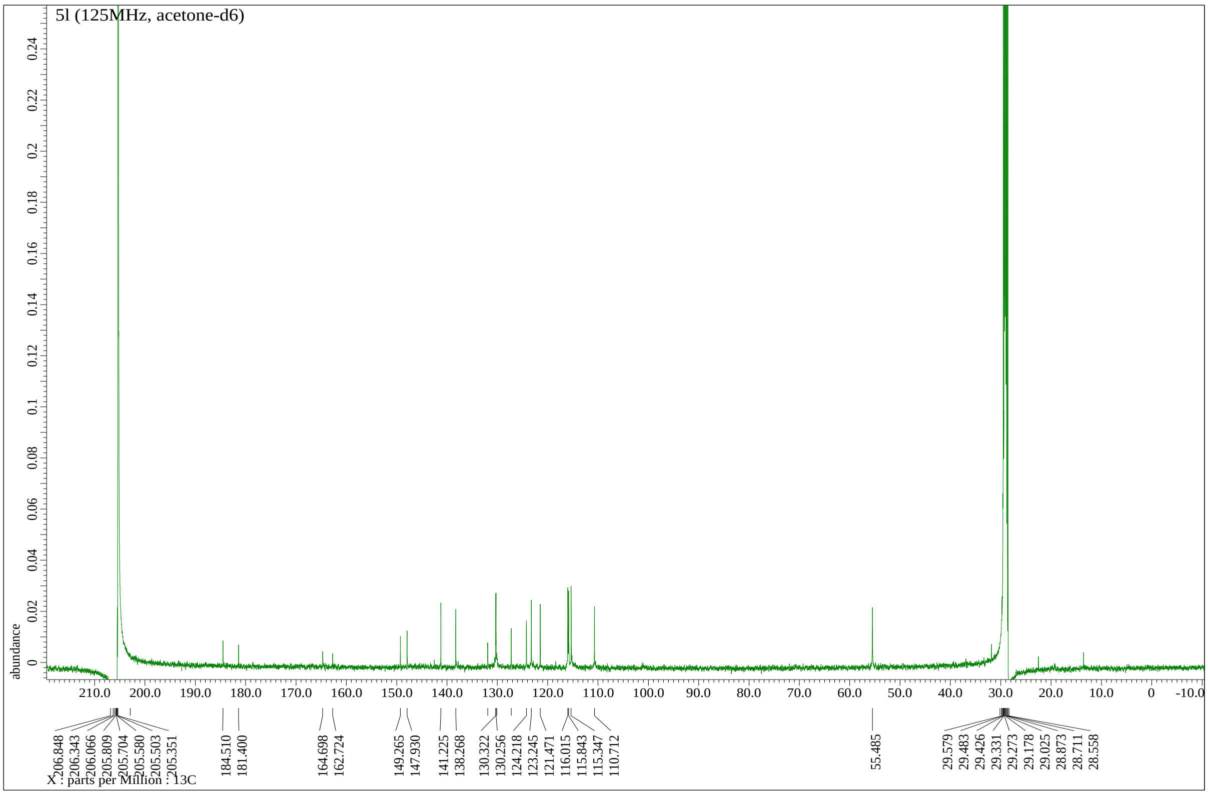

Figure S17. ^{1}H (top) and ^{13}C NMR (bottom) spectra in acetone-d_6 of **5l**

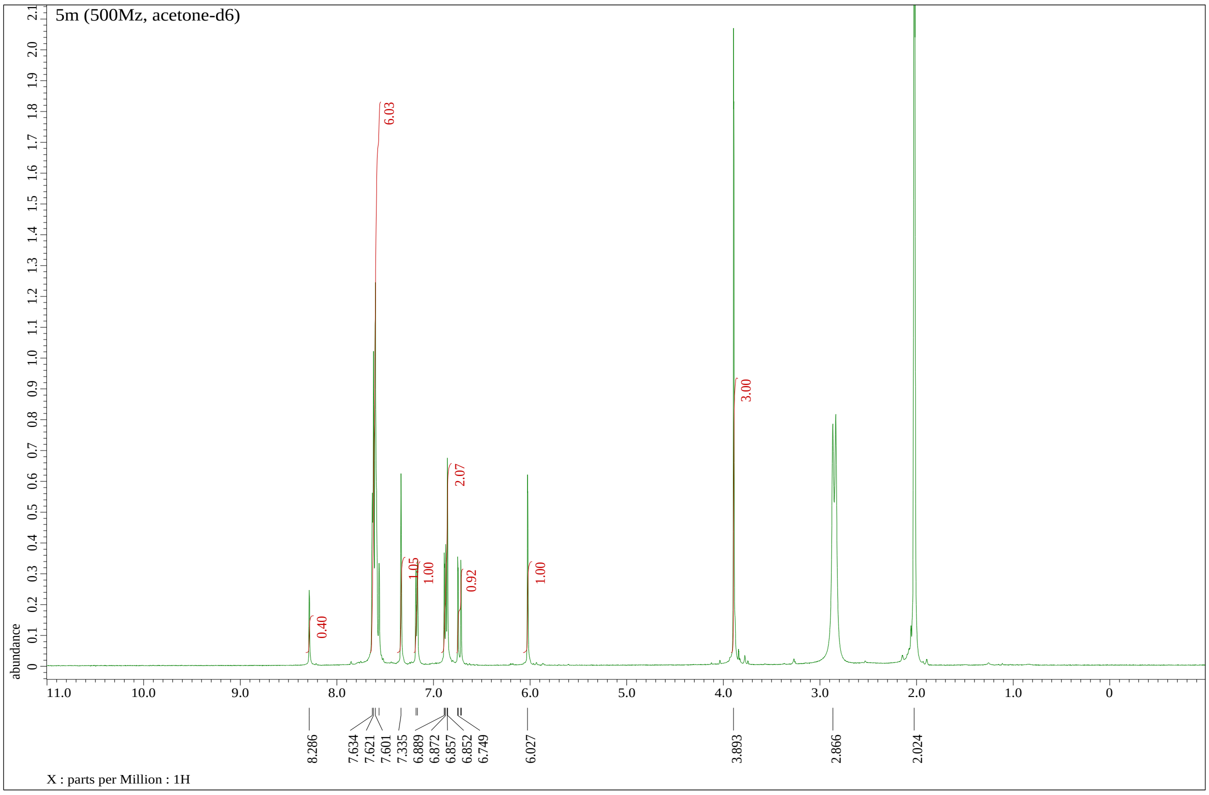

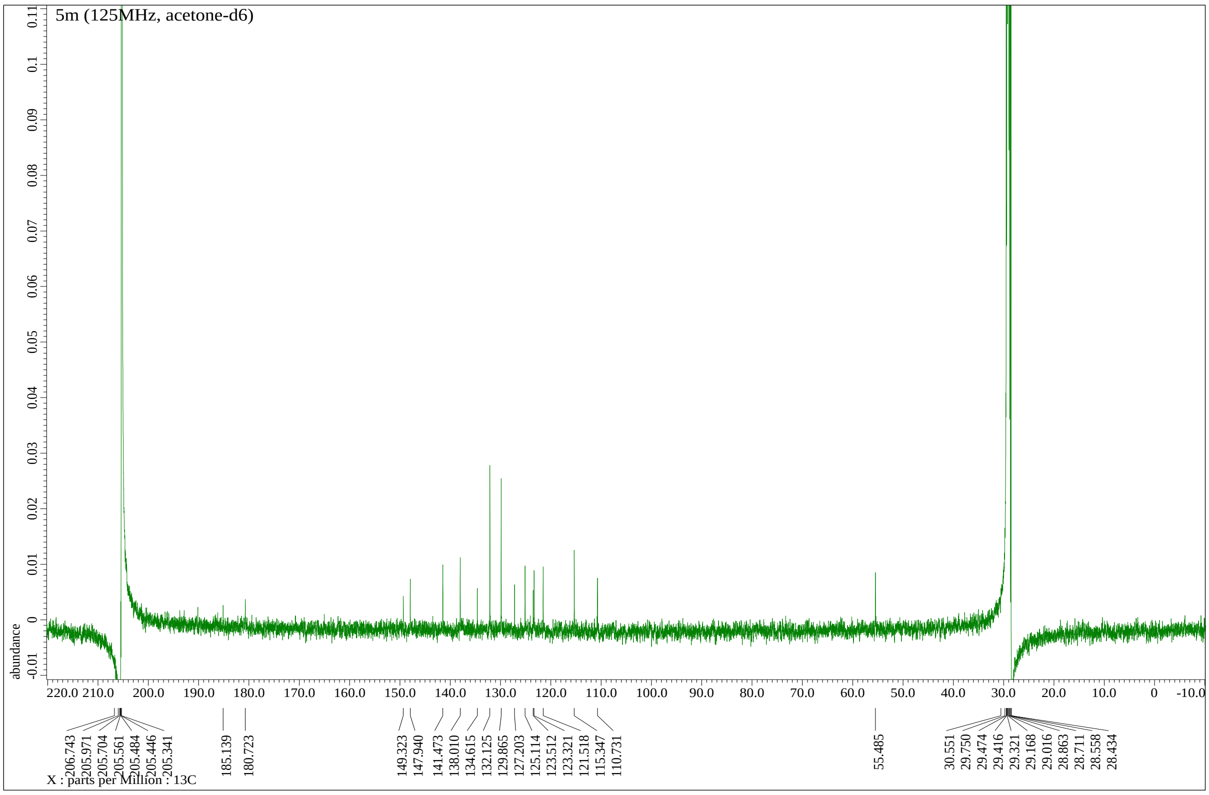

Figure S18. ^{1}H (top) and ^{13}C NMR (bottom) spectra in acetone-d_6 of **5m**

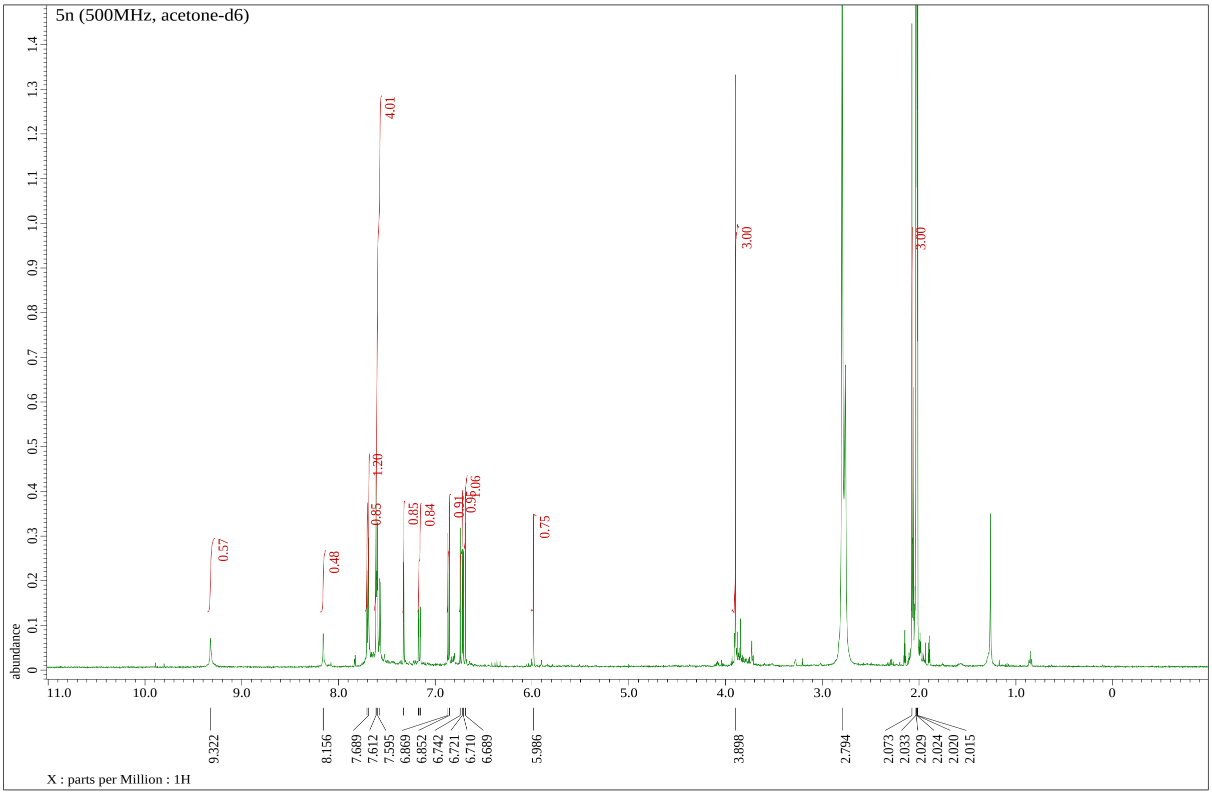

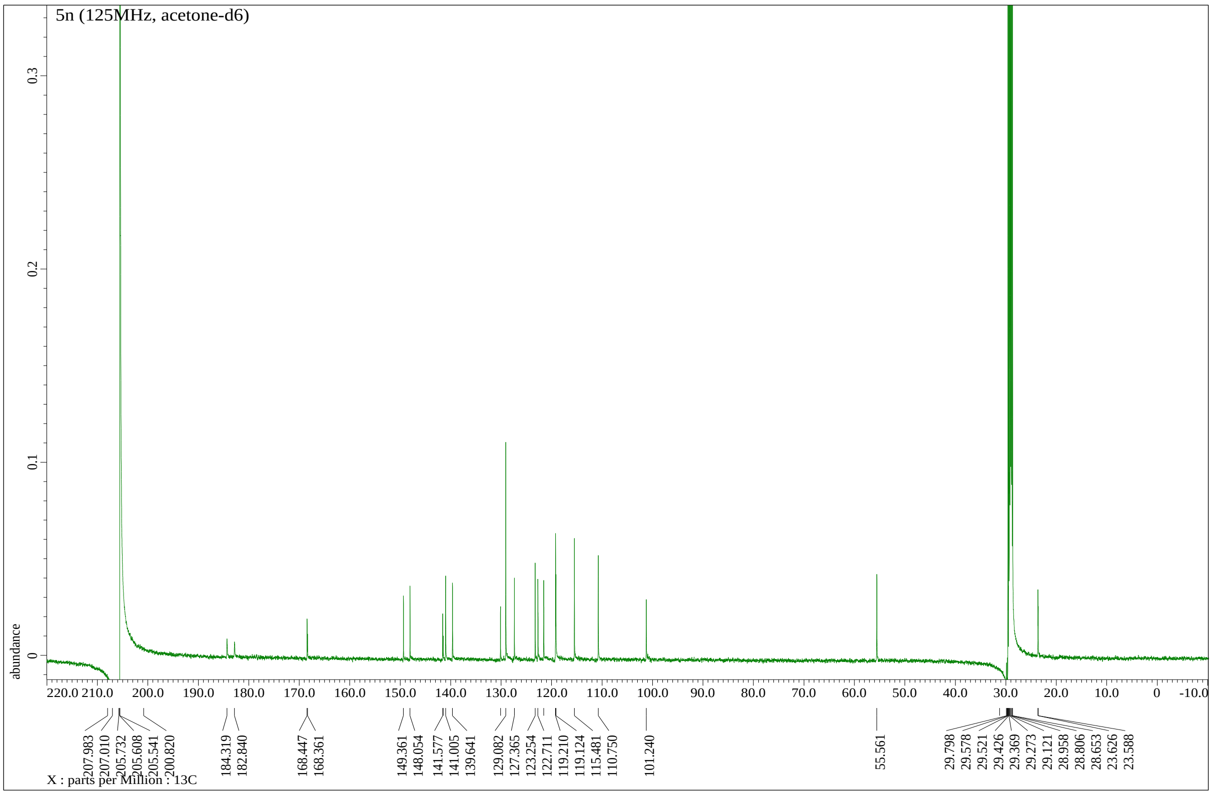

Figure S19. ^{1}H (top) and ^{13}C NMR (bottom) spectra in acetone-d_6 of **5n**

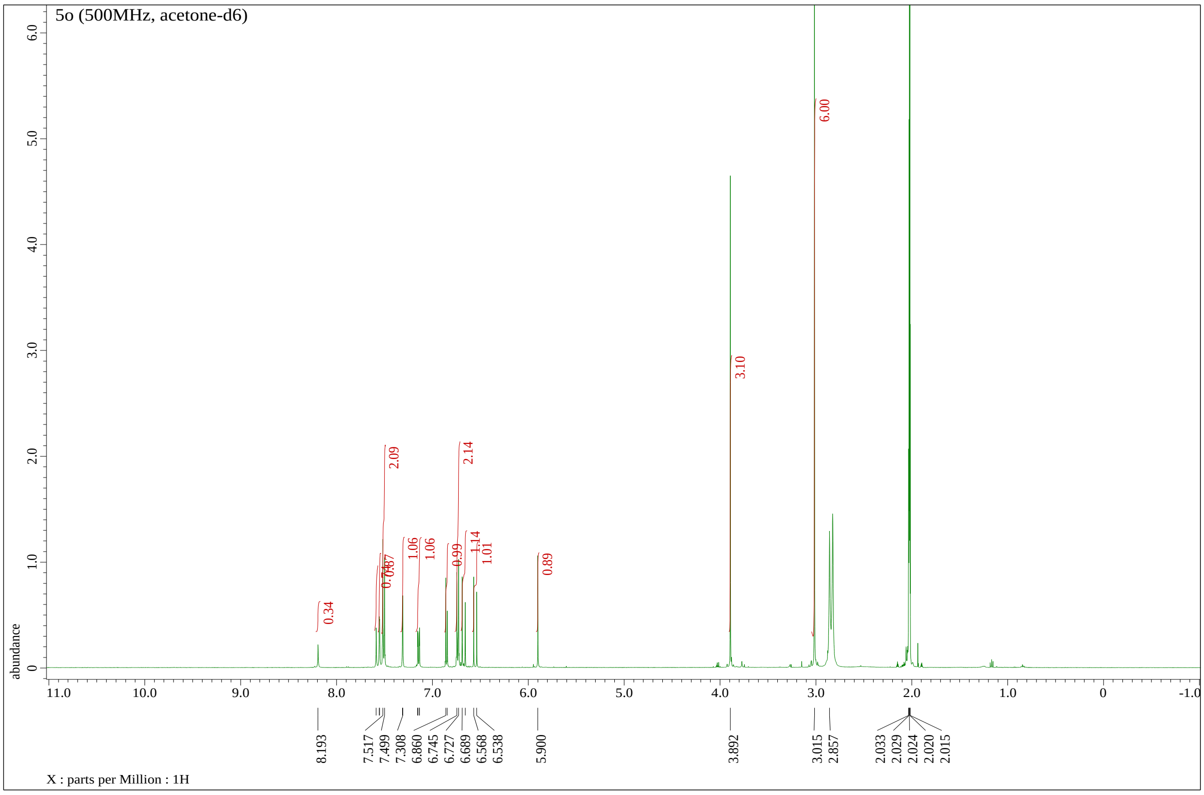

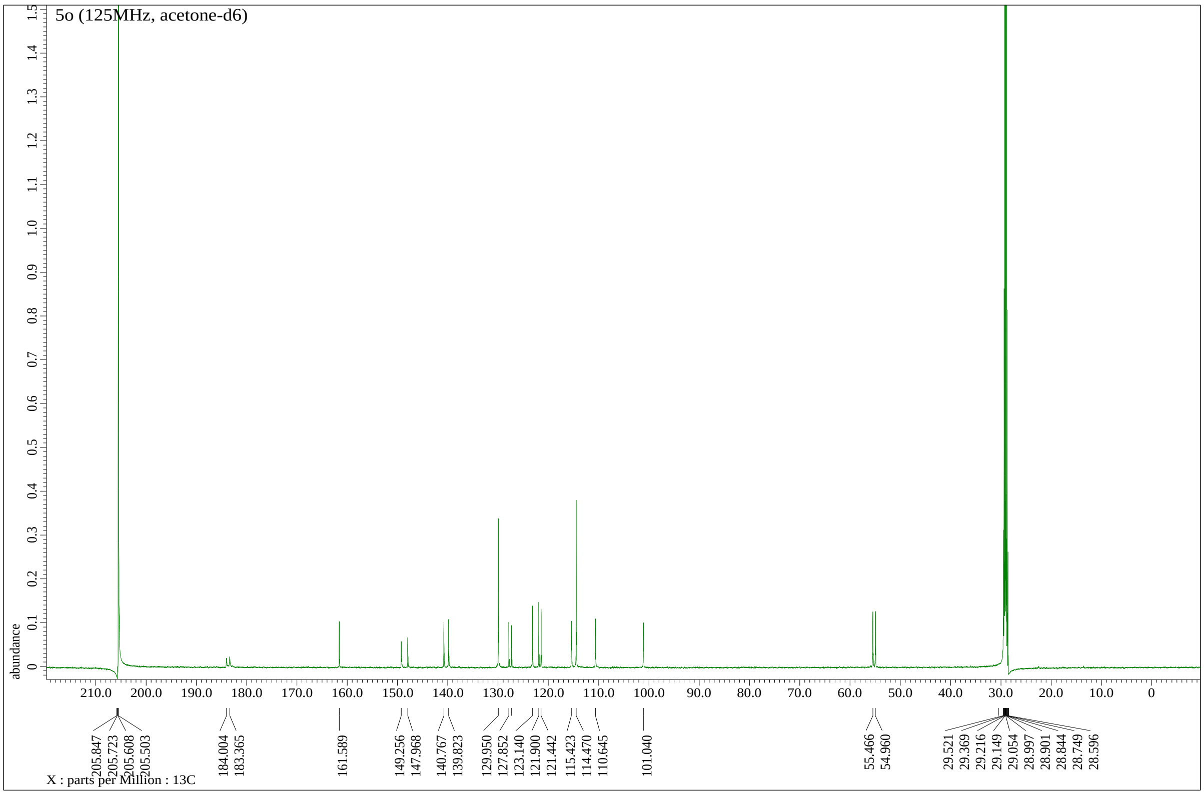

Figure S20. ^{1}H (top) and ^{13}C NMR (bottom) spectra in acetone-d_6 of **5o**

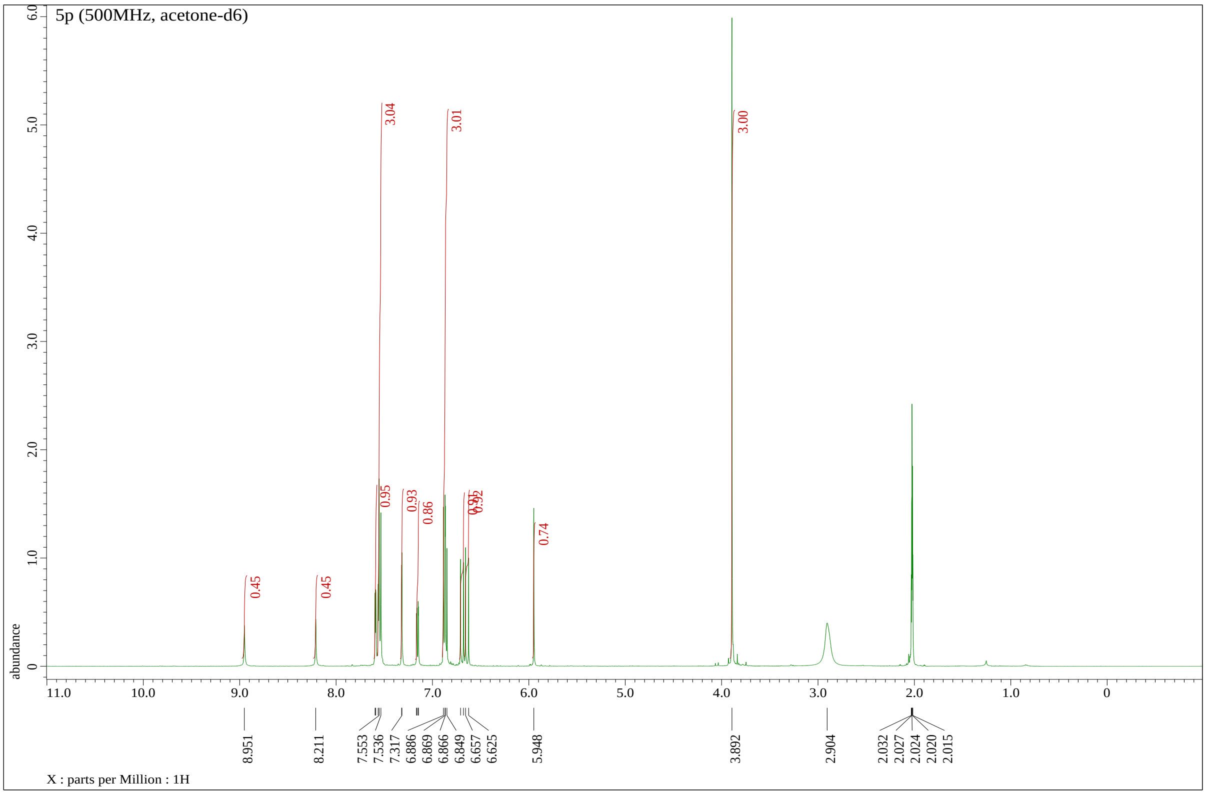

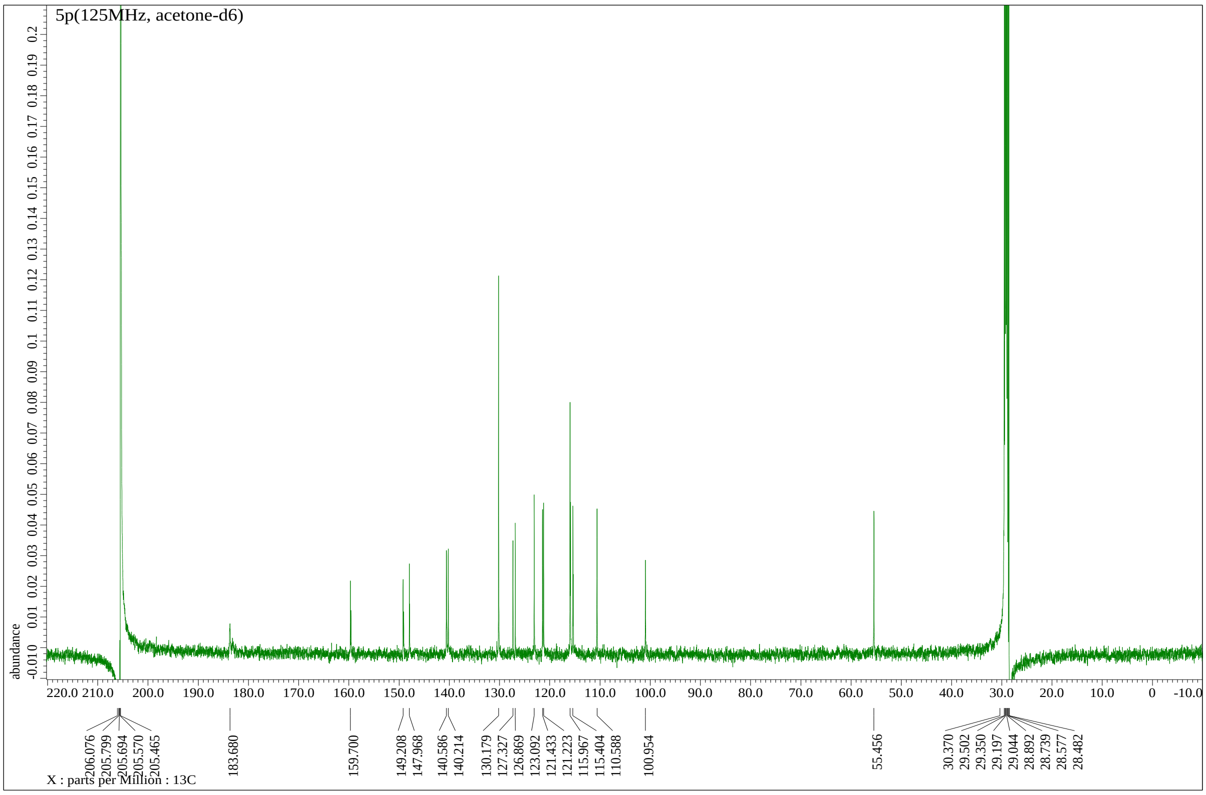

Figure S21. ^{1}H (top) and ^{13}C NMR (bottom) spectra in acetone-d_6 of **5p**

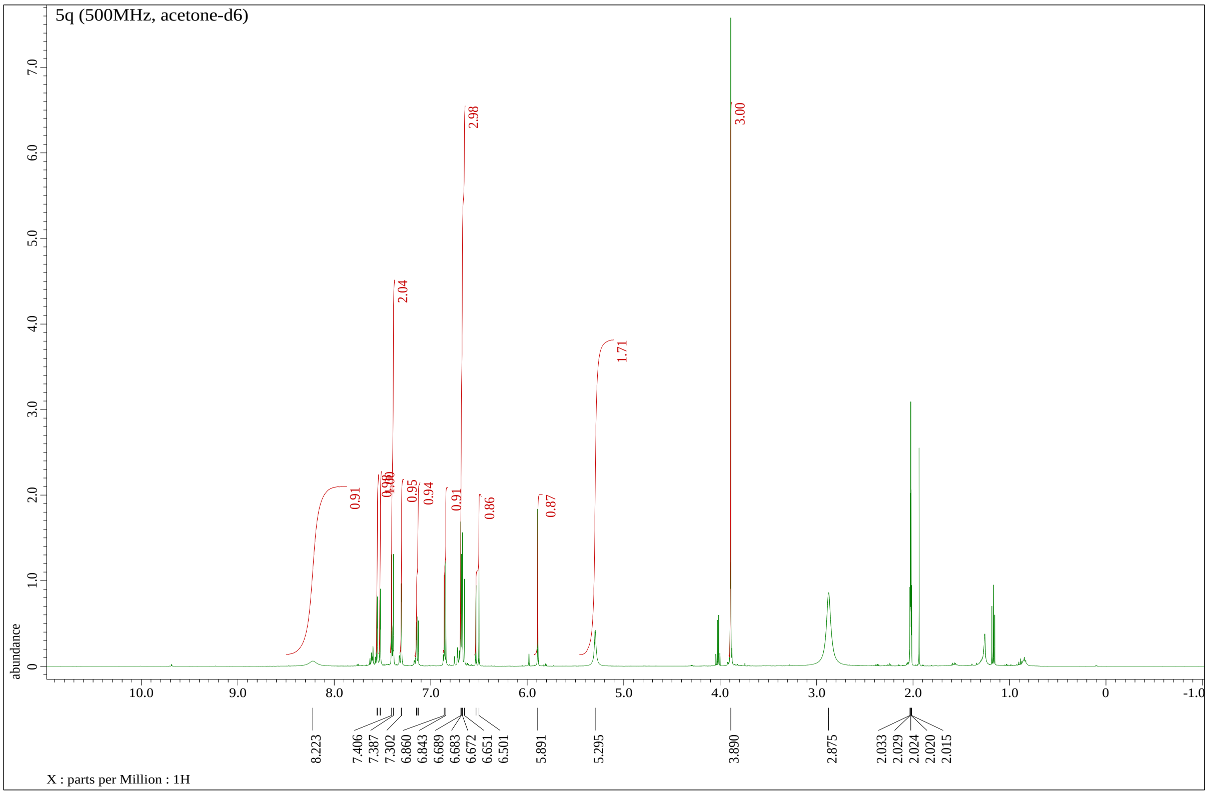

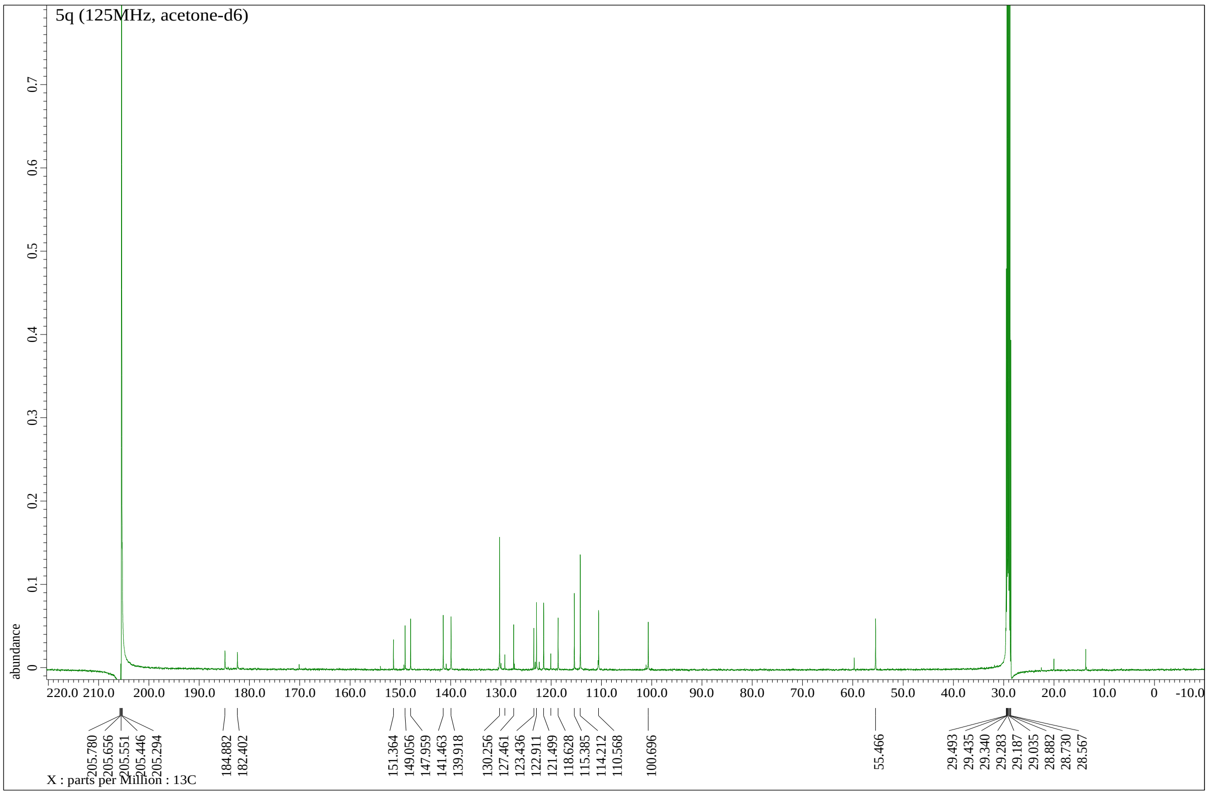

Figure S22. ^{1}H (top) and ^{13}C NMR (bottom) spectra in acetone-d_6 of **5q**

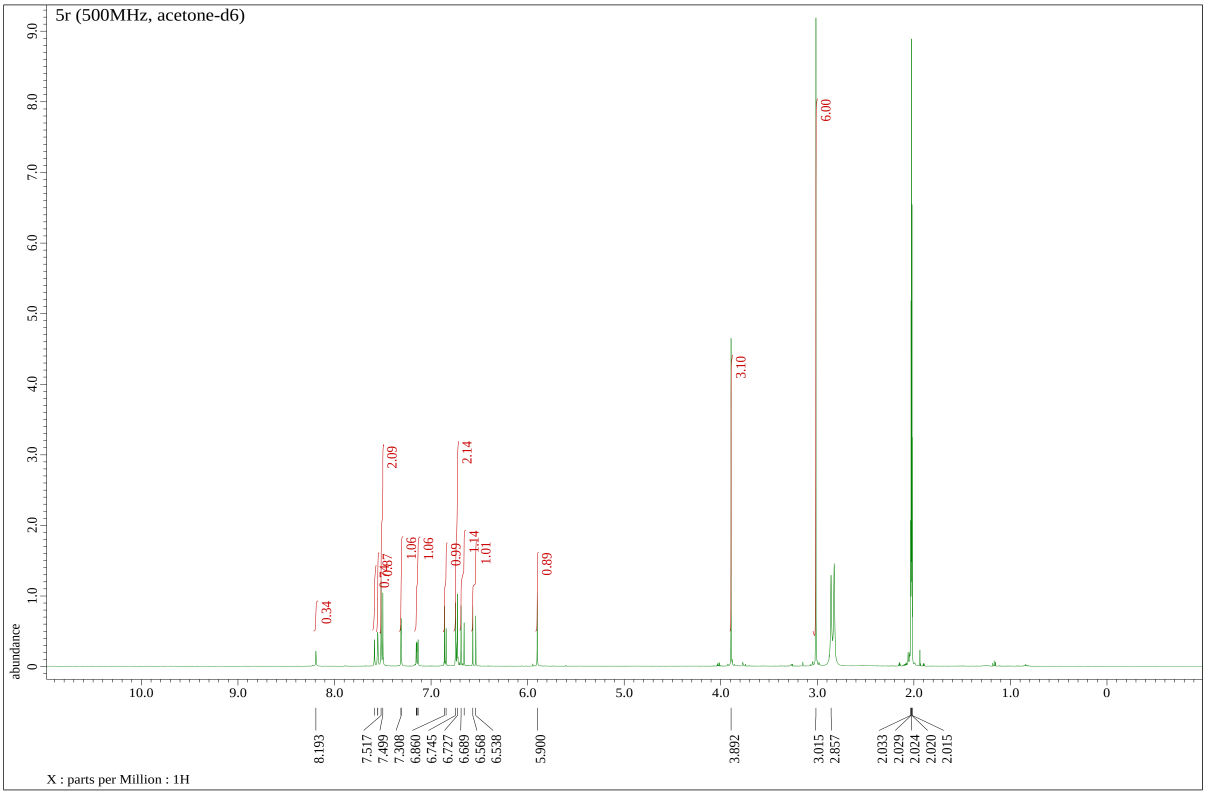

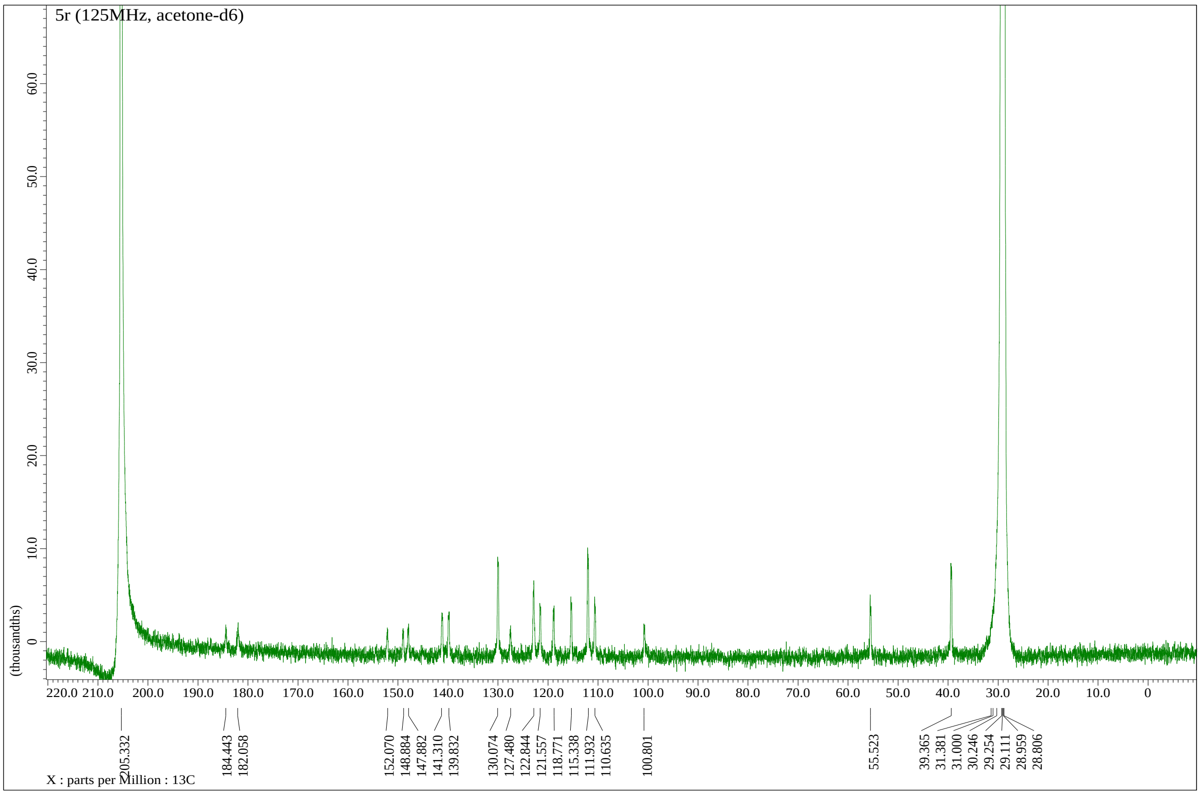

Figure S23. ^{1}H (top) and ^{13}C NMR (bottom) spectra in acetone-d_6 of **5r**

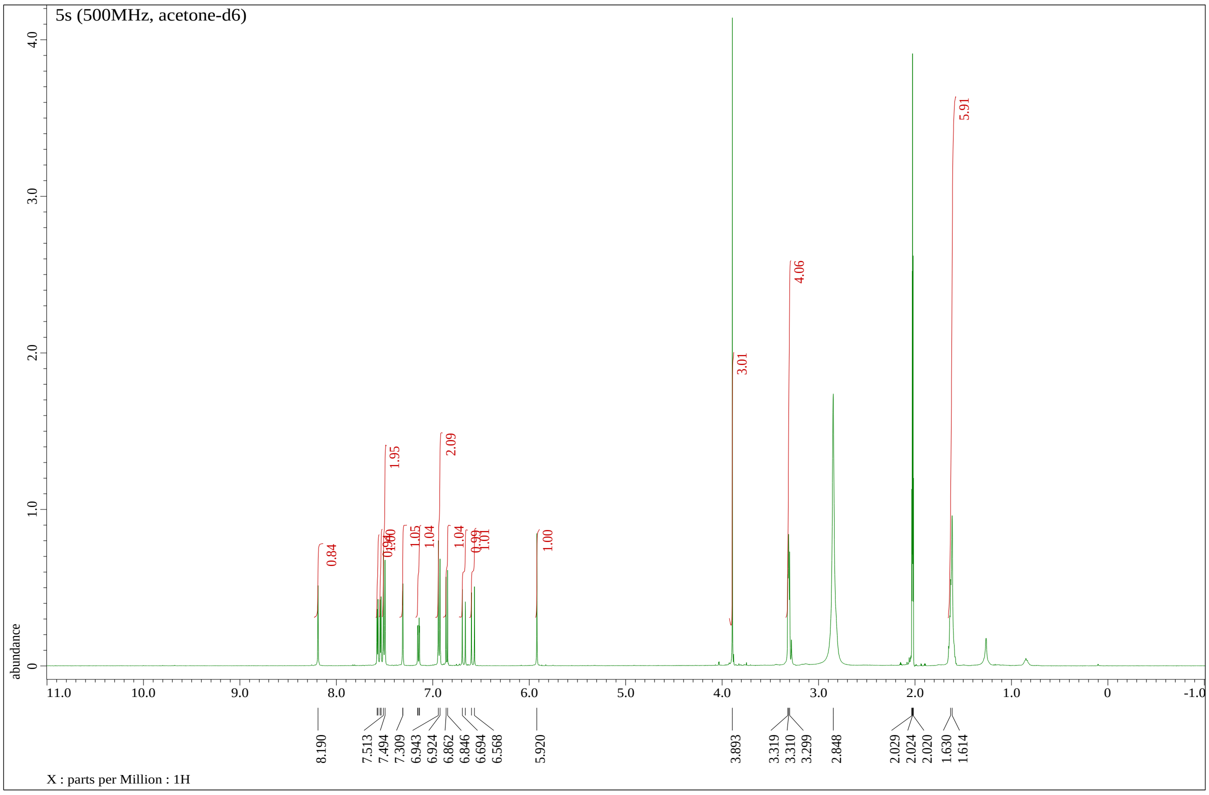

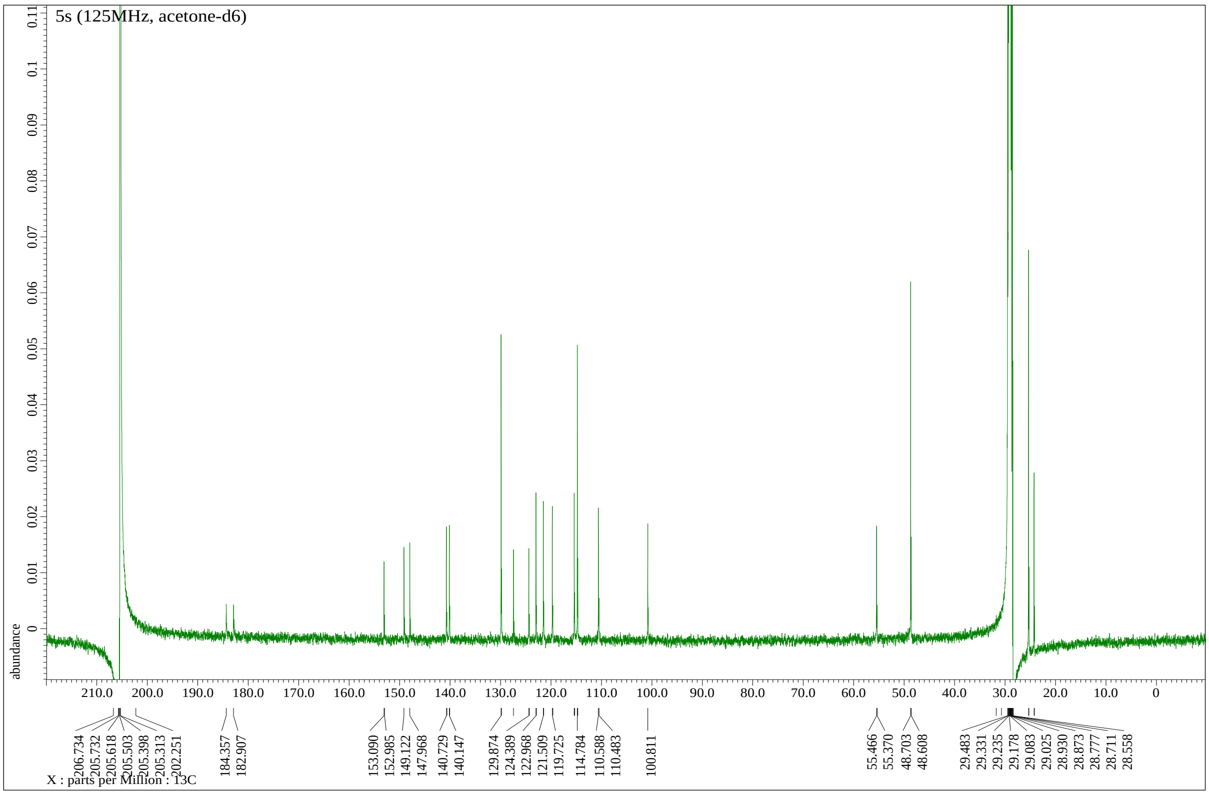

Figure S24. ^{1}H (top) and ^{13}C NMR (bottom) spectra in acetone-d_6 of **5s**

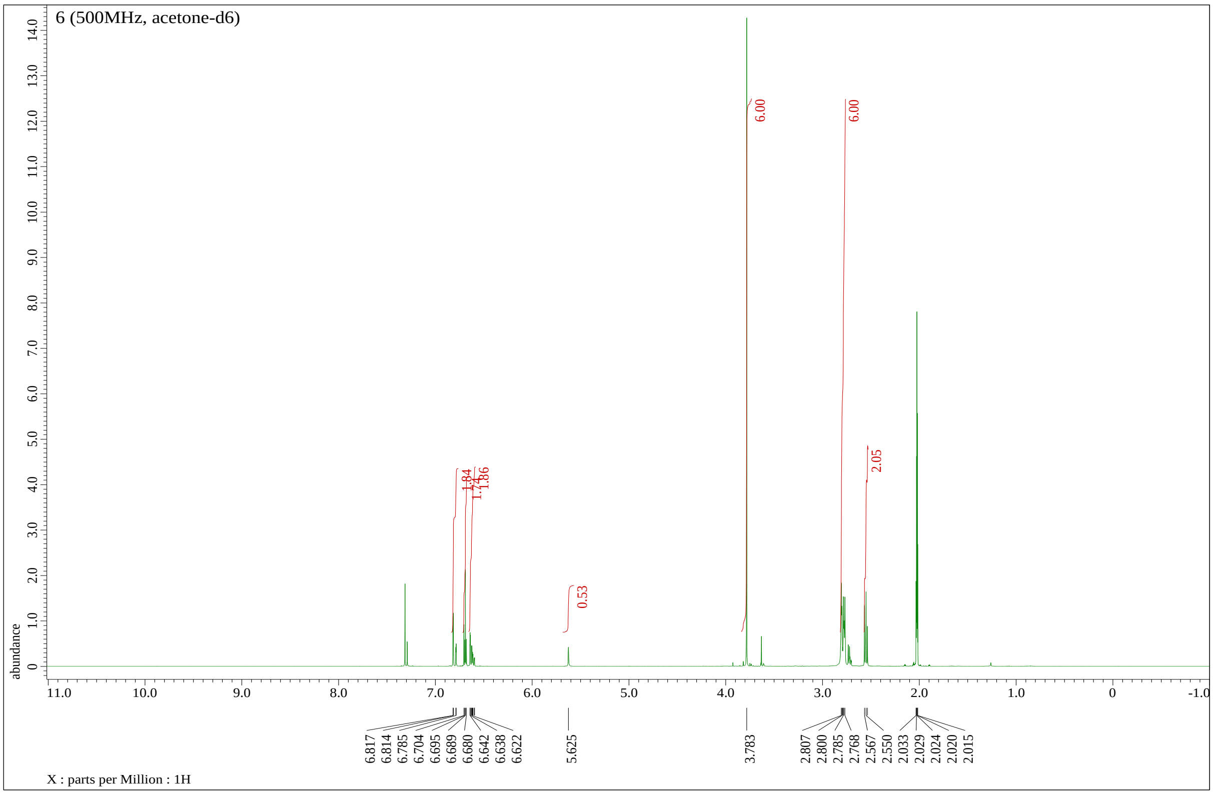

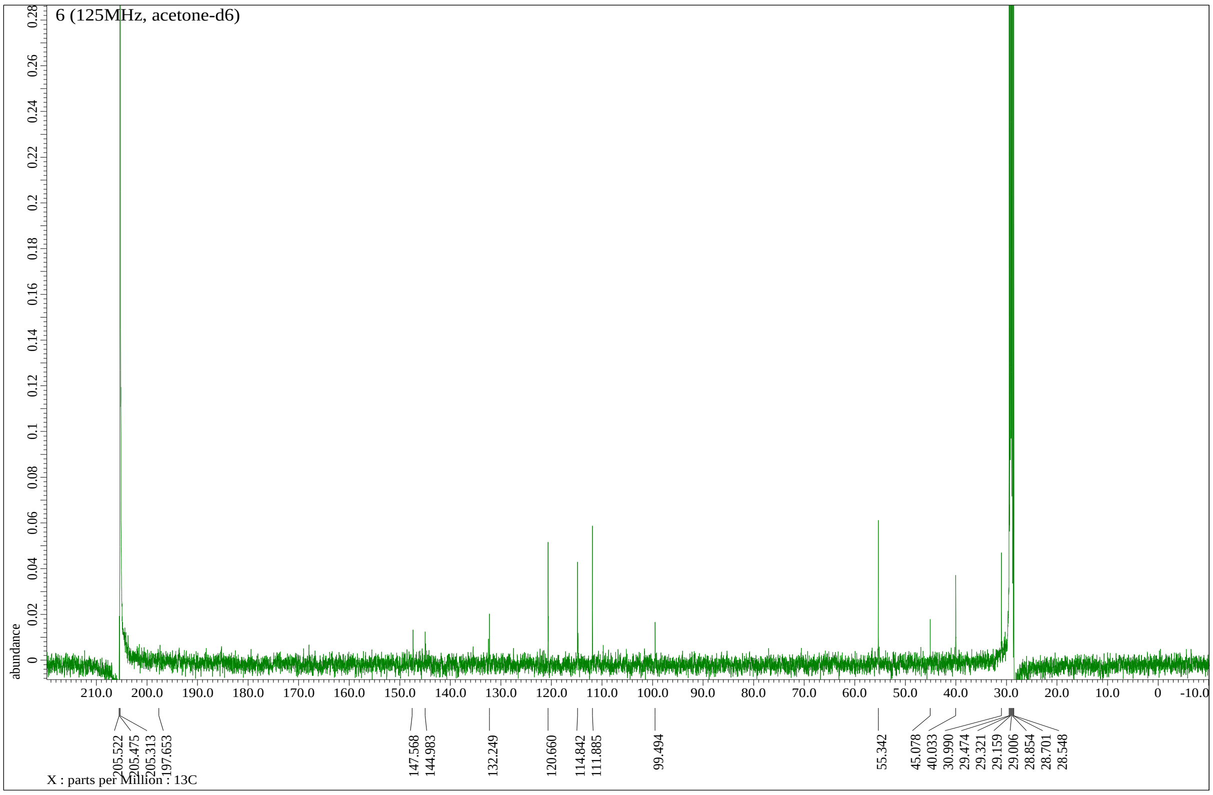

Figure S25. ^{1}H (top) and ^{13}C NMR (bottom) spectra in acetone-d_6 of **6**

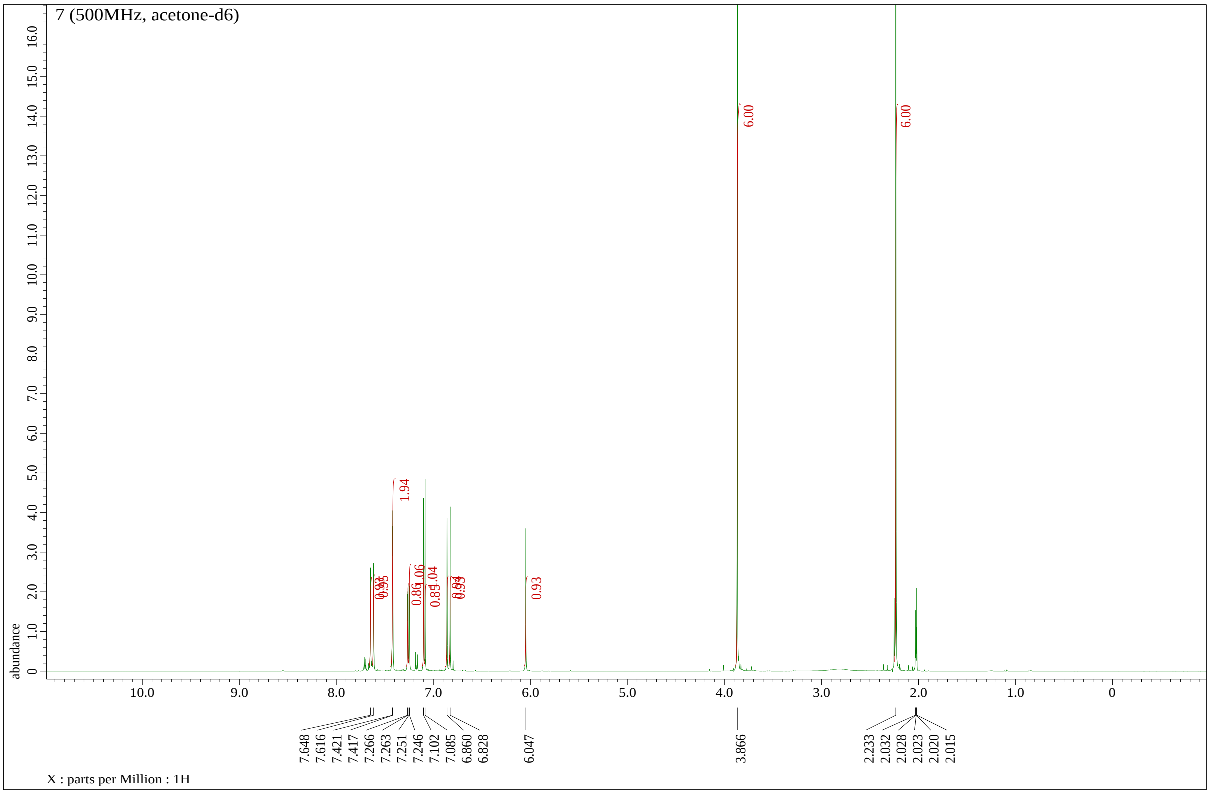

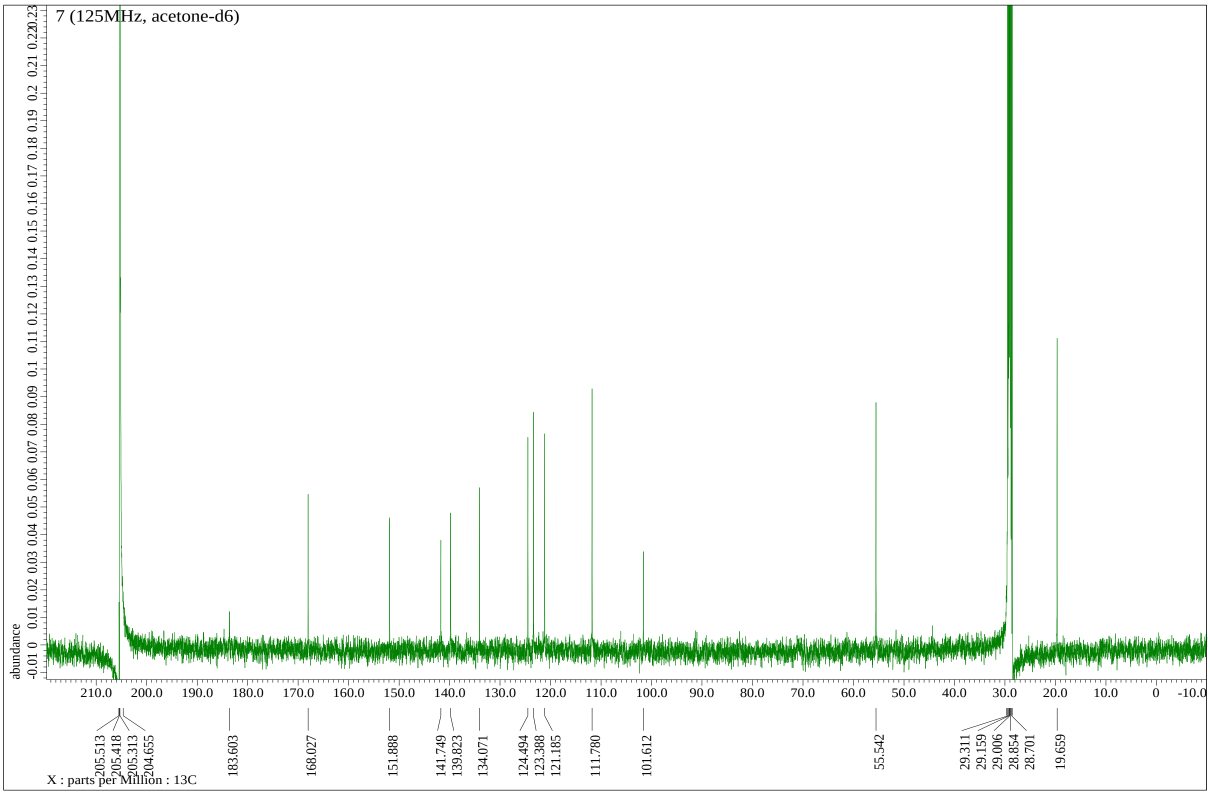

Figure S26. ^{1}H (top) and ^{13}C NMR (bottom) spectra in acetone-d_6 of **7**

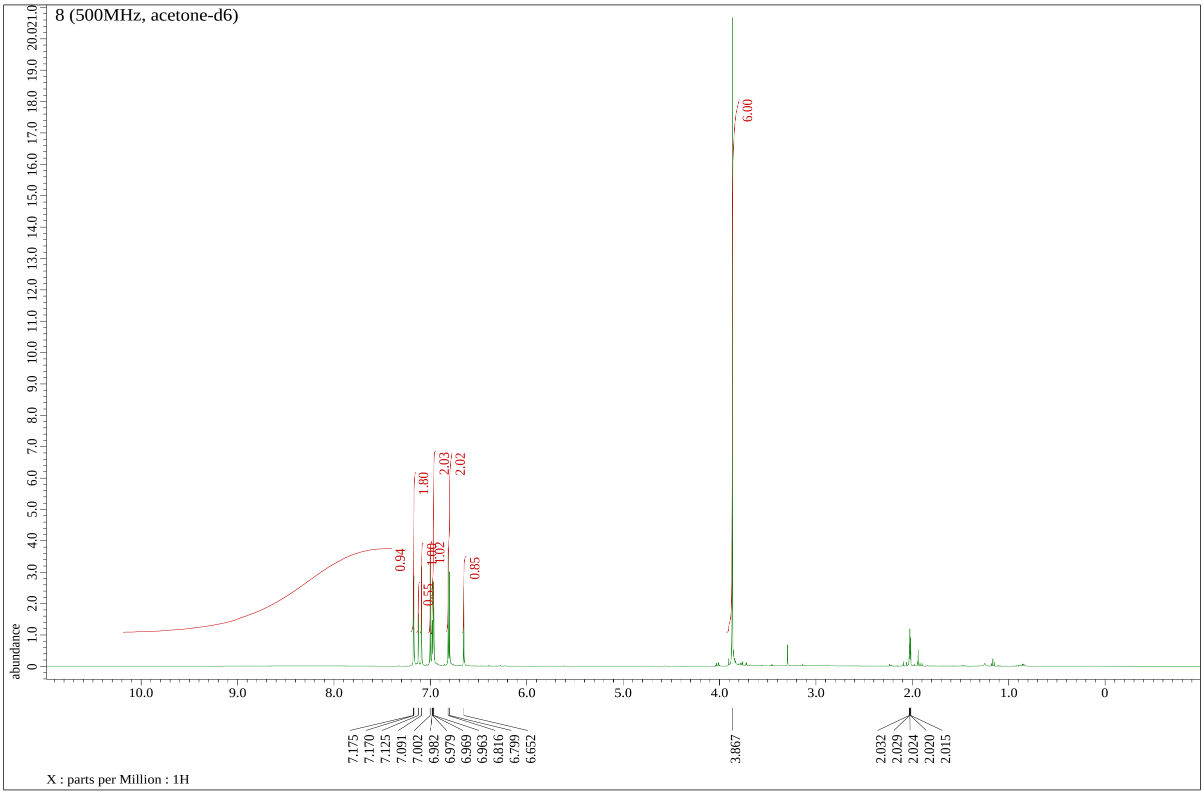

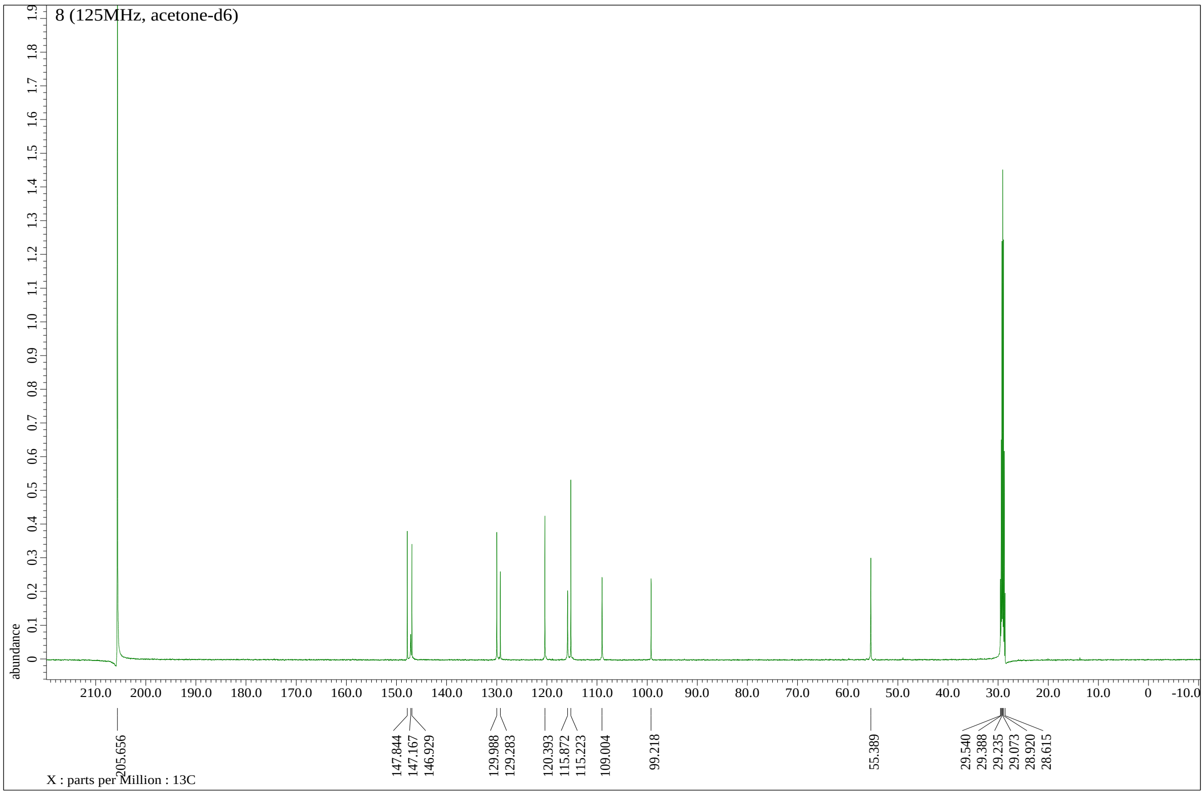

Figure S27. ^{1}H (top) and ^{13}C NMR (bottom) spectra in acetone-d_6 of **8**

2. Preparation of enzyme

```
   1 gaaggagcggctttaacagagaaaacggacatattcgaaagcgggcgtaacggtaaacca
     E  G  A  A  L  T  E  K  T  D  I  F  E  S  G  R  N  G  K  P
  61 aataaagatggaatcaagagttatcgtattccagcacttctcaagacagataaaggaact
     N  K  D  G  I  K  S  Y  R  I  P  A  L  L  K  T  D  K  G  T
 121 ttgatcgcaggtgcagatgaacgccgtctccattcgagtgactggggtgatatcggtatg
     L  I  A  G  A  D  E  R  R  L  H  S  S  D  W  G  D  I  G  M
 181 gtcatcagacgtagtgaagataatggtaaaacttggggtgaccgagtaaccattaccaac
     V  I  R  R  S  E  D  N  G  K  T  W  G  D  R  V  T  I  T  N
 241 ttacgtgacaatccaaaagcttctgacccatcgatcggttcaccagtgaatatcgatatg
     L  R  D  N  P  K  A  S  D  P  S  I  G  S  P  V  N  I  D  M
 301 gtgttggttcaagatcctgaaaccaaacgaatcttttctatctatgacatgttcccagaa
     V  L  V  Q  D  P  E  T  K  R  I  F  S  I  Y  D  M  F  P  E
 361 gggaagggaatctttggaatgtcttcacaaaaagaagaagcctacaaaaaaatcgatgga
     G  K  G  I  F  G  M  S  S  Q  K  E  E  A  Y  K  K  I  D  G
 421 aaaacctatcaaatcctctatcgtgaaggagaaaagggagcttataccattcgagaaaat
     K  T  Y  Q  I  L  Y  R  E  G  E  K  G  A  Y  T  I  R  E  N
 481 ggtactgtctatacaccagatggtaaggcgacagactatcgcgttgttgtagatcctgtt
     G  T  V  Y  T  P  D  G  K  A  T  D  Y  R  V  V  V  D  P  V
 541 aaaccagcctatagcgacaaggggggatctatacaagggtaaccaattactaggcaatatc
     K  P  A  Y  S  D  K  G  D  L  Y  K  G  N  Q  L  L  G  N  I
 601 tacttcacaacaaacaaaacttctccatttagaattgccaaggatagctatctatggatg
     Y  F  T  T  N  K  T  S  P  F  R  I  A  K  D  S  Y  L  W  M
 661 tcctacagtgatgacgacgggaagacatggtcagcgcctcaagatattactccgatggtc
     S  Y  S  D  D  D  G  K  T  W  S  A  P  Q  D  I  T  P  M  V
 721 aaagccgattggatgaaattcttgggtgtaggtcctggaacaggaattgtacttcggaat
     K  A  D  W  M  K  F  L  G  V  G  P  G  T  G  I  V  L  R  N
 781 gggcctcacaagggacggattttgataccggtttatacgactaataatgtatctcactta
     G  P  H  K  G  R  I  L  I  P  V  Y  T  T  N  N  V  S  H  L
 841 aatggctcgcaatcttctcgtatcatctattcagatgatcatggaaaaacttggcatgct
     N  G  S  Q  S  S  R  I  I  Y  S  D  D  H  G  K  T  W  H  A
 901 ggagaagcggtcaacgataaccgtcaggtagacggtcaaaagatccactcttctacgatg
     G  E  A  V  N  D  N  R  Q  V  D  G  Q  K  I  H  S  S  T  M
 961 aacaatagacgtgcgcaaaatacagaatcaacggtggtacaactaaacaatggagatgtt
     N  N  R  R  A  Q  N  T  E  S  T  V  V  Q  L  N  N  G  D  V
1021 aaactctttatgcgtggtttgactggagatcttcaggttgctacaagtaaagacggagga
     K  L  F  M  R  G  L  T  G  D  L  Q  V  A  T  S  K  D  G  G
1081 gtgacttgggagaaggatatcaaacgttatccacaggttaaagatgtctatgttcaaatg
     V  T  W  E  K  D  I  K  R  Y  P  Q  V  K  D  V  Y  V  Q  M
```

```
1141 tctgctatccatacgatgcacgaaggaaaagaatacatcatcctcagtaatgcaggtgga
     S  A  I  H  T  M  H  E  G  K  E  Y  I  I  L  S  N  A  G  G
1201 ccgaaacgtgaaaatgggatggtccacttggcacgtgtcgaagaaaatggtgagttgact
     P  K  R  E  N  G  M  V  H  L  A  R  V  E  E  N  G  E  L  T
1261 tggctcaaacacaatccaattcaaaaaggagagtttgcctataattcgctccaagaatta
     W  L  K  H  N  P  I  Q  K  G  E  F  A  Y  N  S  L  Q  E  L
1321 ggaaatggggagtatggcatcttgtatgaacatactgaaaaaggacaaaatgcctatacc
     G  N  G  E  Y  G  I  L  Y  E  H  T  E  K  G  Q  N  A  Y  T
1381 ctatcatttagaaaatttaattgggacttttgagcaaagatctgatttctcctaccgaa
     L  S  F  R  K  F  N  W  D  F  L  S  K  D  L  I  S  P  T  E
1441 gcgaaagtgaagcgaactagagagatgggcaaaggagttattggcttggagttcgactca
     A  K  V  K  R  T  R  E  M  G  K  G  V  I  G  L  E  F  D  S
1501 gaagtattggtc
     E  V  L  V
```

Figure S28. Nucleotide sequence and protein sequence of synthesized *S. pneumoniae* neuraminidase A (NanA)

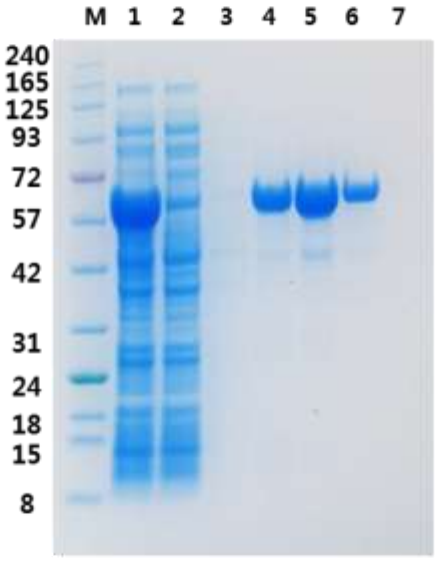

lane 1 : Crude NanA
lane 2 : Flow through
lane 3 : washing (Binding buffer)
lane 4 : Elution (50 mM imidazole)
lane 5 : Elution (100 mM imidazole)
lane 6 : Elution (200 mM imidazole)
lane 7 : Elution (300 mM imidazole)

Figure S29. Expression and purification of *S. pneumoniae* NanA.

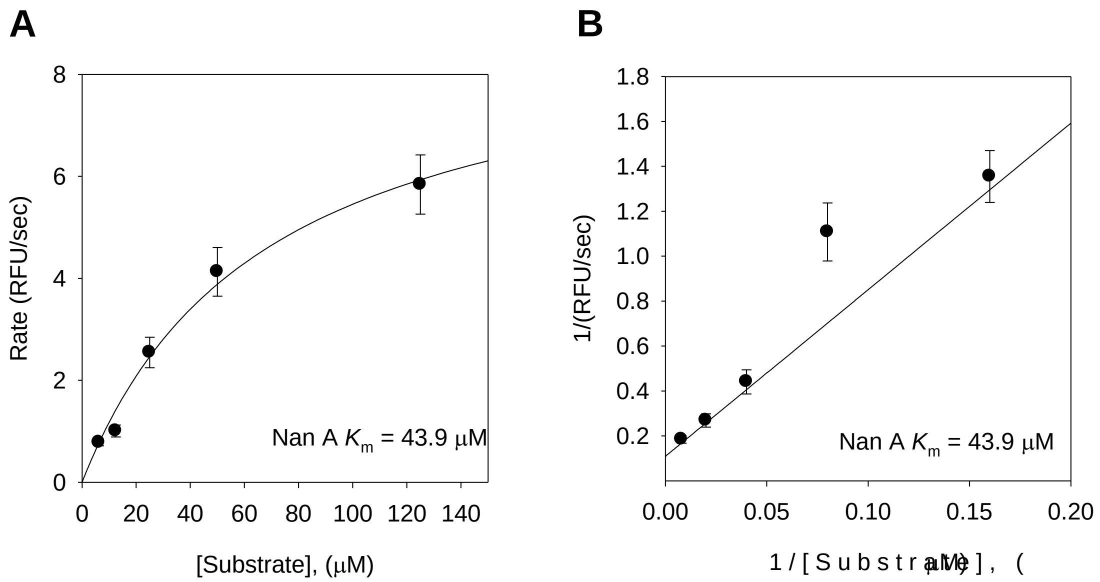

Figure S30. Michaelis-Menten plots (A) and Linewever-Burk plot (B) of *S. pneumoniae* NanA K_m values. The reaction was performed at various substrate concentrations to obtain enzyme K_m values. SigmaPlot was used to fit the kinetic data using Michaelis-Menten and Lineweaver-Burk double reciprocal plots.

[A] **4a**

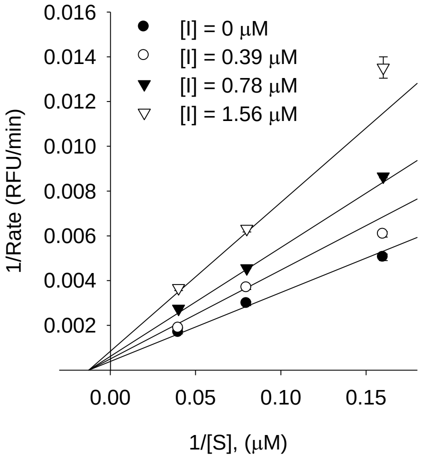

[B] **4e**

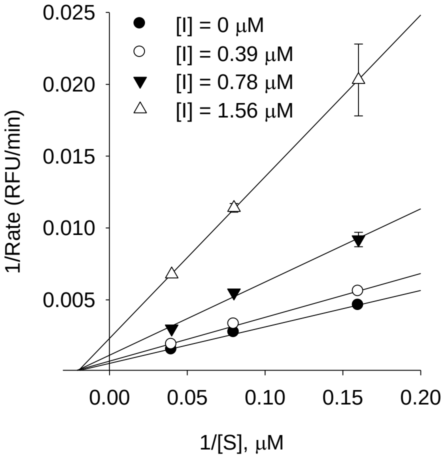

[C] **5q**

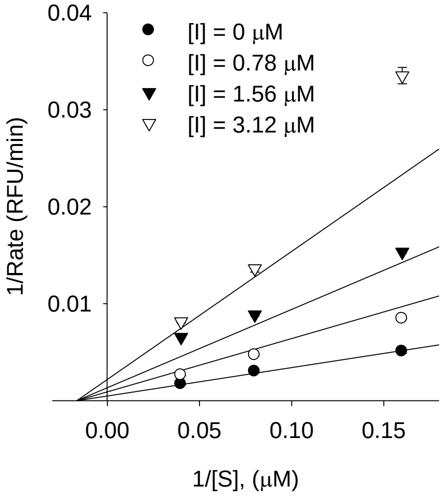

[D] **5e**

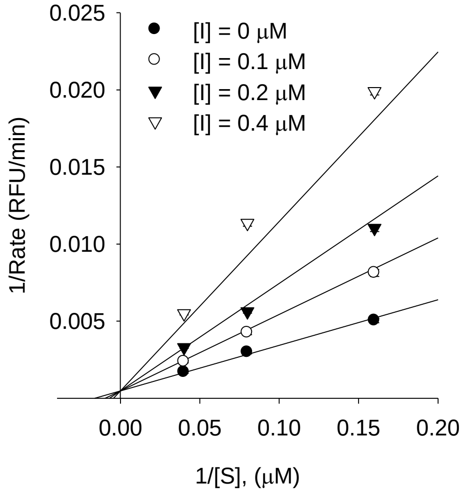

Figure S31. Graphical determination of the inhibition type for compounds **4a**, **4e**, **5q** and **5e**. Lineweaver-Burk (A-D) and Dixon (E-H) plots for the inhibitory activity of compounds **4a**, **4e**, **5q** and **5e**, respectively, against *S. pneumoniae* NanA hydrolysis activity in the presence of different substrate concentrations.

[E] **4a**

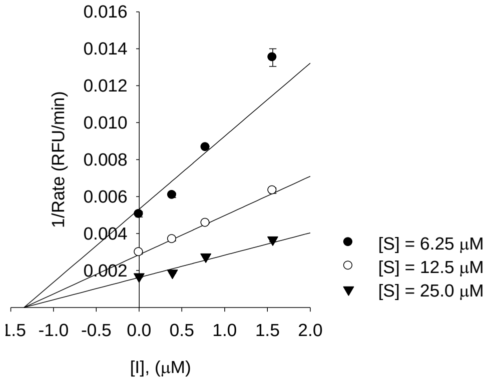

[F] **4e**

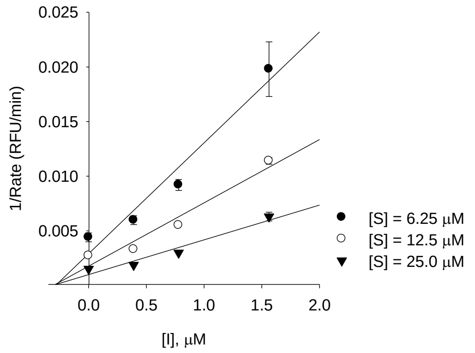

[G] **5q**

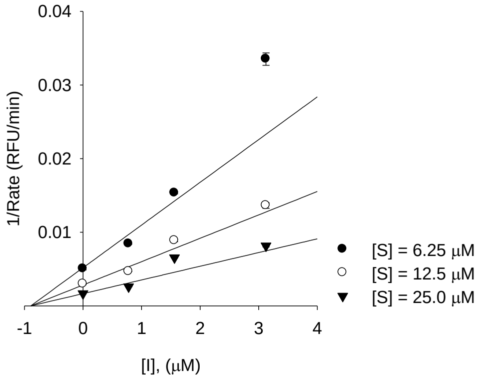

[H] **5e**

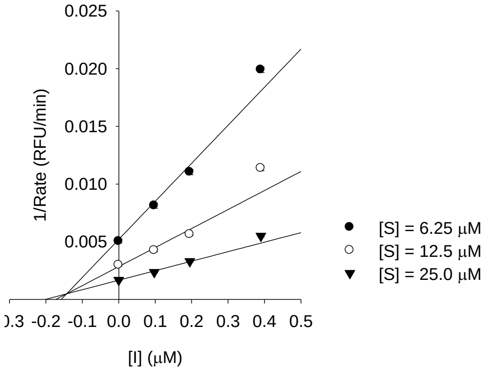

Figure S31. Graphical determination of the inhibition type for compounds **4a**, **4e**, **5q** and **5e**. Lineweaver-Burk (A-D) and Dixon (E-H) plots for the inhibitory activity of compounds **4a**, **4e**, **5q** and **5e**, respectively, against *S. pneumoniae* NanA hydrolysis activity in the presence of different substrate concentrations (continued).
